# Lymph node colonization induces tissue remodeling via immunosuppressive fibroblast-myeloid cell niches supporting metastatic tolerance

**DOI:** 10.1016/j.ccell.2026.01.003

**Published:** 2026-01-29

**Authors:** Maximilian Haist, Marc-A. Baertsch, Nathan E. Reticker-Flynn, Guolan Lu, Tim N. Kempchen, Pauline Chu, Gustavo Vazquez, Han Chen, John B. Sunwoo, Weiruo Zhang, Eyiwunmi Laseinde, Bonny Adami, Stefanie Zimmer, Justus Kaufman, Quynh Thu Le, Andrew J. Gentles, Christina S. Kong, Sylvia K. Plevritis, Yury Goltsev, John W. Hickey, Garry P. Nolan

**Affiliations:** 1 Department of Microbiology and Immunology, Stanford University School of Medicine, Stanford, CA, USA; 2 Department of Pathology, Stanford University School of Medicine, Stanford, CA, USA; 3 Department of Dermatology, University Medical Center Mainz, Mainz, Germany; 4 Department of Hematology, Oncology and Rheumatology, Heidelberg University Hospital, Heidelberg, Germany; 5 Clinical Cooperation Unit Molecular Hematology/Oncology, German Cancer Research Center, Heidelberg, Germany; 6 Department of Otolaryngology, Stanford University, Stanford, CA, USA; 7 Molecular Biosciences/Cancer Biology Program, Heidelberg University, Heidelberg, Germany; 8 German Cancer Research Center, DKFZ, Heidelberg, Germany; 9 Institute of Experimental Oncology, University Hospital Bonn, Bonn, Germany; 10 Stanford Cancer Institute, Stanford University, Stanford, CA, USA; 11 Department of Biomedical Data Science, Stanford University, Stanford, CA, USA; 12 Departments of Biological Sciences and Computer Science, Purdue University, West Lafayette, IN, USA; 13 Department of Radiation Oncology, Stanford University, Stanford, CA, USA; 14 Department of Pathology, University Medical Center Mainz, Mainz, Germany; 15 Department of Radiation Oncology and Radiotherapy, University Medical Center Mainz, Mainz, Germany; 16 Department of Medicine, Stanford University, Stanford, CA, USA; 17 Department of Radiology, Stanford University, Stanford, CA, USA; 18 Department of Biomedical Engineering, Duke University, Durham, NC, USA; 19 These authors contributed equally; 20 Lead contact

## Abstract

Lymph node (LN) colonization in cancer is linked to poor prognosis. Evidence suggests that LN colonization induces systemic immunosuppression, facilitating distant metastasis. We investigated LN-mediated immunosuppression in patients with head-and-neck cancer using spatial proteomics, spatial transcriptomics, and an *in vivo* model of melanoma LN metastasis. Both primary tumors and paired LNs of nodal-positive patients exhibit enhanced interferon-γ signaling and an enrichment of immunosuppressive myeloid cells and cancer-associated fibroblasts (CAFs). The spatial intersection of these myeloid-CAF-enriched niches with perifollicular T cell zones and LN follicles is linked to enhanced T cell dysfunction and Treg activation therein, thereby driving architectural LN remodeling. These immune suppressive changes extend to adjacent non-tumor-involved LN regions and nearby tumor-free LNs, but were not detected in LNs of non-cancer patients, reflecting a systemic effect that compromises anti-tumor immunity beyond the tumor-involved LN. Hence, our findings establish LN colonization as an active driver of systemic immunosuppression, facilitating metastatic progression.

## In brief

Haist et al. demonstrate that lymph node (LN) colonization induces systems-wide LN remodeling in patients with head-and-neck cancer using multiplex imaging, spatial transcriptomics, and a melanoma model of LN metastasis. The authors identify spatially organized immunomodulatory myeloid/CAF niches as key features of metastatic tolerance that associate with T cell dysfunction and Treg activation beyond the tumor- colonized LN.

## Graphical abstract

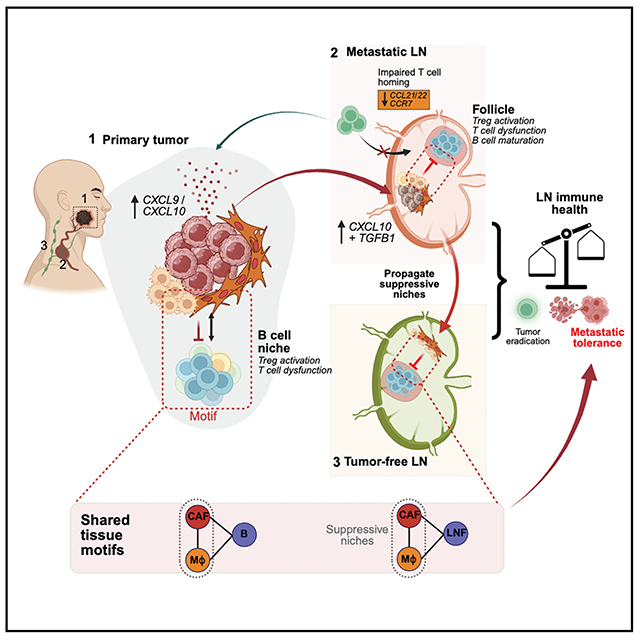

## INTRODUCTION

Cancer metastasis is a complex, multi-step process that leads to the dissemination of cancer cells from the primary tumor site to distant organs and remains the primary cause of cancer-associated mortality.^1^ While distant metastases are often preceded by the colonization of tumor-draining lymph nodes (TDLNs),^2^ the role of LN metastasis extends beyond serving as a prognostic indicator.^3^ Increasing evidence suggests that LN colonization may actively contribute to metastatic progression by modulating immune responses within and beyond the tumor-infiltrated LN.^4,5^ LNs function as key sites of antigen presentation, where dendritic cells (DCs) prime naive T cells to mount anti-tumor responses,^6^ a process essential for effective immunotherapy, such as immune checkpoint inhibitors (ICB).^7–10^ While the role of LNs as essential orchestrators of anti-tumor immunity has been extensively studied, it remains incompletely understood how LN colonization alters this immunological function to promote immunosuppression and disease progression.^11^

Recent studies from murine models of LN metastasis indicate that LN colonization does not simply serve as a conduit for tumor dissemination but may actively induce systemic immune suppression.^5,12^ In particular, LN colonization has been shown to generate antigen-specific regulatory T cells (Tregs), which promote immune tolerance and facilitate distant metastasis.^5^ Similarly, in patients with melanoma, Treg accumulation in TDLNs is associated with increased risk of recurrence within 24 months of surgery.^12^ While these findings suggest a link between LN metastases and immune dysfunction, the broader impact of LN colonization on the cellular and architectural organization of the LN microenvironment—and its consequences for systemic immunity—remains poorly defined.

Here, we investigate immunological and spatial remodeling of primary tumors (PTs) and paired TDLNs in patients with human head and neck squamous cell carcinoma (HNSCC) with or without LN metastasis using spatial multiomic profiling and *in vivo* validation in a murine model of LN metastasis. PTs and affected LNs of nodal-positive patients show pronounced interferon signaling and are enriched in spatially interacting immunosuppressive myeloid cells and cancer-associated fibroblasts (CAFs), causing widespread architectural and functional immune changes that extend to adjacent tumor-free LNs but are absent in nodal-negative and cancer-naive patients, reinforcing the role of LNs as active regulators of immune dysfunction rather than passive sites of metastasis.

## RESULTS

### Spatial profiling of the tumor microenvironments in primary human head and neck squamous cell carcinoma and paired tumor-draining lymph nodes

**Figure 1.**
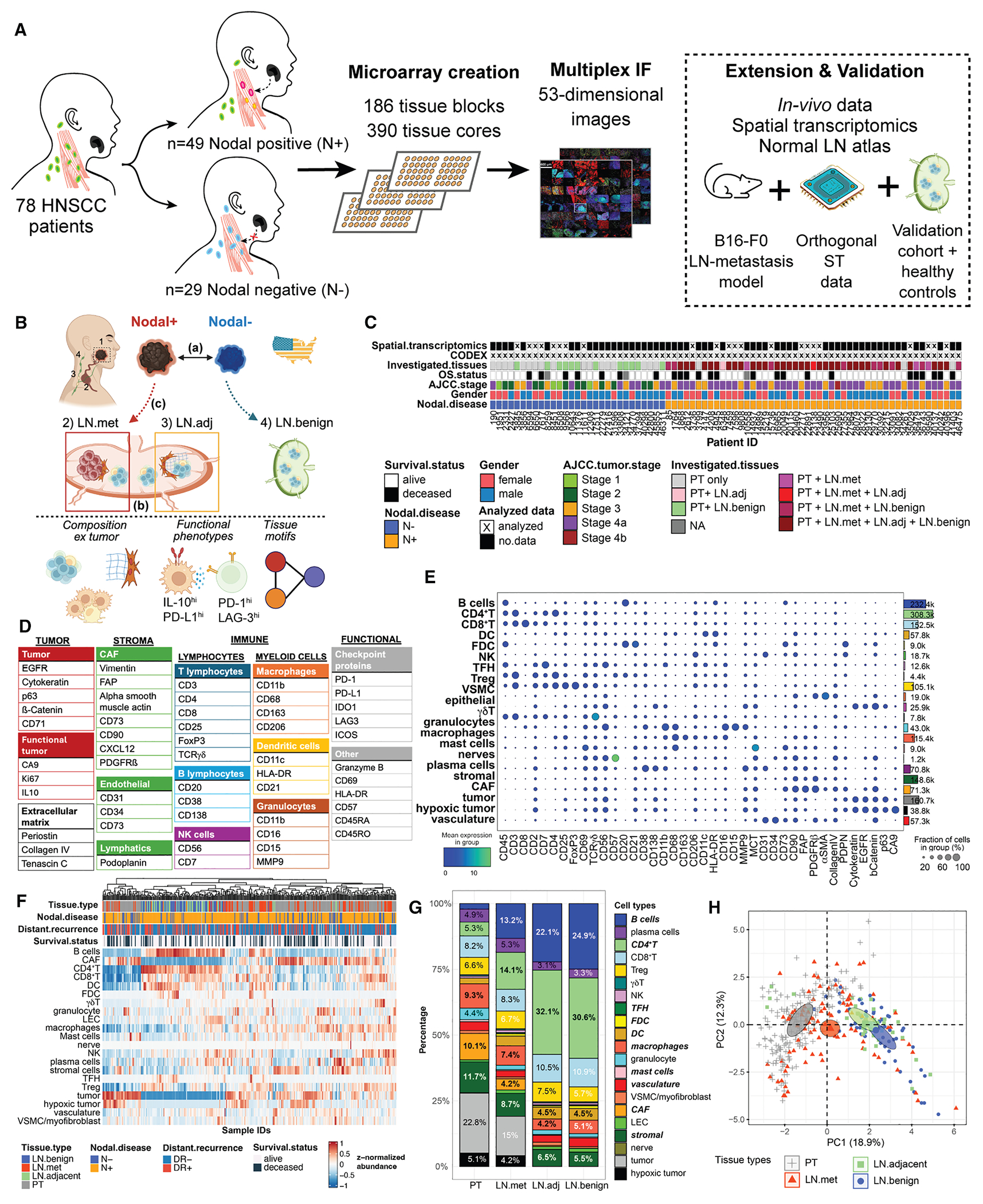
Spatial profiling of paired primary tumors and LN metastases in patients with HNSCC (A) Experimental and analytical framework. (B) Investigated tissues from the HNSCC discovery cohort (top) and extracted TME-features (bottom). Primary endpoints included the identification of correlates for nodal disease from primary tumors (PTs) (a), motifs of metastatic tolerance within TDLNs (b), and cross-tissue signatures of nodal disease (c). (C) Nodal status and clinical patient characteristics within the HNSCC cohort. (D) Antibody panel used for CODEX multiplex imaging. (E) Dotplot of z-normalized marker expression for all identified cell-types with bar charts representing corresponding cell counts. (F) Heatmap for cell proportions per tissue sample (*n* = 390) annotated for clinical features ordered by euclidian clustering. (G) Stacked barplot of aggregated cell type proportions (in %) stratified by tissue type, including PT (*n* = 189), LN.met (*n* = 95), LN.adjacent (*n* = 43) and LN.benign (*n* = 63). (H) Results from PCA using major non-tumor cell type abundances within all investigated tissue regions (*n* = 390). Individual samples are colored by tissue type. Ellipses represent 95% confidence interval for each tissue type based on multivariate normal distribution assumption. Abbreviations: N+, nodal-positive; N−, nodal-negative; IF, immunofluorescence; DR+, distant recurrence; DR-, without distant recurrence, LEC, lymphatic endothelial cells; LN.adj, adjacent LN; TFH, T follicular helper cells; VSMC, vascular smooth muscle cells. See also Figures S1 and S2.

We identified a cohort of 78 patients with HNSCC with paired PT and LN tissue samples, which we profiled using spatial proteomic imaging and orthogonal validation through spatial transcriptomics (ST). We validated and expanded our results using an independent cohort of patients with HNSCC and healthy controls, which we complemented through *in vivo* extension of our data in a murine B16-F0 melanoma model (Figure 1A). We used this experimental framework to characterize cellular and spatial features underlying altered immune responses following LN colonization (Figure 1B). Our HNSCC discovery cohort comprised 49 patients with nodal disease and 29 patients without nodal involvement at initial diagnosis. As expected, patients with nodal disease presented with a significantly shorter distant metastasis-free survival (Figures S1A and S1B), while other clinical-demographic features were comparable between both patient groups (Table 1). This observation agrees with previous findings that nodal involvement associates with a higher risk of distant recurrence (DR), which might be mediated by tumor-immune evasion at the LN-level.^5^

To reveal systems-wide immunomodulatory effects driven by LN colonization, we collected paired PT, LN metastases (LN.met), adjacent microscopically non-tumor involved regions of metastatic LNs (LN.adjacent), and non-tumor involved TDLNs (LN.benign) from each patient with nodal disease, while for nodal-negative patients, we collected paired PT and noninvolved LNs (Figures 1C and S1C). Using this unique set of paired tissue samples, we constructed 3 tissue microarrays (TMAs) from archival tumor specimens (Figures S1D and S1E) with a total of 390 tissue cores, which we profiled using a dedicated panel of 53 antibodies for the multiplex imaging technique Co-Detection-by-Indexing (CODEX) (Figure 1D). All antibodies were titrated and optimized in primary HNSCC (Figures S2A–S2C) to ensure binding specificity.^13^

Following image pre-processing^14^ and cell segmentation with lateral spillover compensation, we used Leiden-based clustering to annotate a total of 1,556,233 cells as described previously.^15^ All cell annotations were stringently inspected, and their accuracy was verified on the original CODEX multiplex images with expert pathologists. Using this approach, we classified 21 major cell types within our dataset (Figure 1E).

We observed differences in the cellular composition of the LN microenvironments between LN.met and their tumor-free counterparts (Figure 1F). Specifically, we observed an enrichment of macrophages, CAFs, and plasma cells within malignant tissues, whereas CD4^+^ T cells and B cells were dominating the microenvironment within LN.benign (Figure 1G). We further characterized these cellular differences using principal component analysis (PCA) on the table of major cell type proportions outside the tumor compartment (Figure 1H). Based on the first two principal components (PCs), we detected distinct differences between tumor-involved and non-involved LNs, as well as a gradual shift in cell type composition from PTs and LN.met. Here, PC1 and PC2 (Figure S1F) had high loadings on B cells, CAF, CD4^+^ T cells, and macrophages, indicating that those cell types might be critical for the unique functional and architectural features between those tissue types.

### Primary tumors are characterized by stromal-enriched immunosuppressive microenvironments but share spatial contexts with paired lymph node metastasis

**Figure 2.**
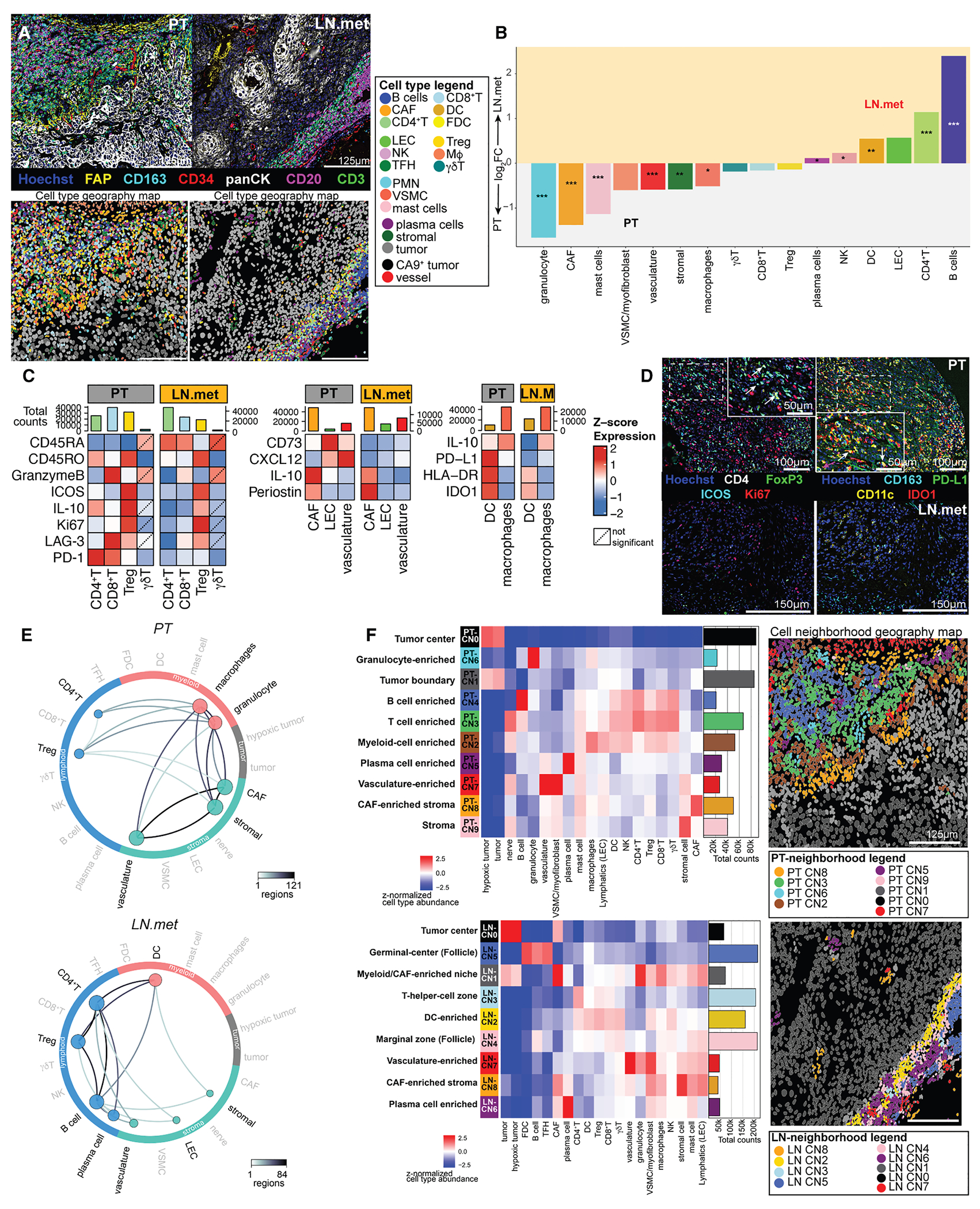
Stromal and immune remodeling in paired primary tumors and LN.met (A) Representative examples of a PT (left) and its paired LN.met (right) with corresponding cell type geography maps (bottom). Scale bar of 125 μm applies to all panels. (B) Waterfall plot shows relative cell-type enrichments between paired PT (*n* = 125) and LN.met regions (*n* = 95). Statistical significance was tested using two-sided Wilcoxon test adjusted for multiple testing, *p < 0.05, **p < 0.01, ***p < 0.001. (C) Heatmaps depict cell-type specific z-normalized expression of selected functional markers between paired PT and LN.met. Statistical comparisons between tissue types were made using Wilcoxon test adjusted for multiple testing. (D) Two representative examples of PTs (top) and LN.met (bottom) showcasing differences in Ki67 and ICOS expression among Treg (left) and PD-L1 and IDO1 expression within myeloid cells (right). (E) Circos plot depicts cellular niche networks characteristic to paired PTs (top) and LN.met (bottom). Nodes represent cell-types involved in such niche networks and edge weights correspond to the number of unique tissue regions with the corresponding interaction. Node size is proportional to connectedness, as measured by eigenvector centrality. (F) Heatmaps for recurring cellular neighborhoods (CNs) in PTs (top) and LN samples (bottom) based on the relative enrichment of the 21 cell types therein. Right panel depicts CN geography maps for the examples shown in (A). Scale bar of 125 μm applies to both panels. See also Figures S3 and S4.

We next dissected the differences between PTs of nodal-positive patients and their paired LN.met in more detail: To specifically assess immune and stromal remodeling associated with LN colonization, we conducted differential enrichment analysis for all cell types outside of the tumor compartment (Figure 2A): We found that granulocytes, CAFs, mast cells, macrophages and vessels were significantly enriched within PTs, while DCs, CD4^+^ T cells and B cells were predominantly observed within LN.met (Figures 2B and S3A). These findings highlight the stromal character of the primary tumor microenvironment (TME), whereas LN.met retain more features of a lymphoid-associated immune landscape, albeit one that is functionally impaired.

A critical feature of high-plex spatial proteomics is the ability to capture phenotypic and functional characteristics of cells, allowing insights into how cell function relates to tissue organization and cell-cell interactions within tissues.^13^ We therefore compared functional characteristics of lymphocyte, myeloid cell, and stromal compartments between paired PT and LN.met. T lymphocytes exert critical roles in anti-tumor immunity, but within an immunosuppressive TME, they can acquire a dysfunctional phenotype marked by the enhanced expression of PD-1, TIM-3, or LAG-3.^16^ Consistent with this, we detected a significant enrichment of PD-1^hi^, LAG-3^hi^ T cells, and activated IL-10^hi^ Tregs within PTs relative to paired LN.met (Figure 2C left; Figure S3B). In agreement, we observed that macrophages, DCs, and CAFs expressed higher levels of immunomodulatory molecules (Figure 2C center and right), including PD-L1 and IL-10, within PTs (Figure 2D). Critically, these tissue-specific compositional and phenotypical differences were not driven by the microscopic selection of tissue regions but were observed regardless of whether the tumor core or tumor edge of PTs or LN.met was chosen (Figures S4A–S4I).

To further characterize the spatial organization of immune and stromal compartments within these tissues, we computationally defined locally recurring niche networks in PTs and paired LN.mets.^17^ Here, we observed that PTs were characterized by a stronger interaction of stromal cells, including CAFs and vasculature, with macrophages and granulocytes (Figure 2E, top). By contrast, paired LN.met exhibited cellular niches of CD4^+^ T cells, B cells, and plasma cells with DCs (Figure 2E, bottom; Figure S4J), emphasizing the structural differences between both tissue types.

Given the pronounced differences in the cellular, phenotypical, and spatial organization of PTs and LNs, we identified cellular neighborhoods (CNs) separately for both tissue entities. Such CNs represent functionally distinct cellular units within the TME and were computed using cell-type enrichments within the k = 15 nearest neighbors of a given index cell (Figure 2F).

We identified 10 distinct CNs in PTs, including well-defined histological units, such as a vasculature-enriched niche and two tumor-associated CNs (Figure 2F, top).

By contrast, LNs were organized into 9 CNs, reflecting known LN architectural features, including LN follicles (LNFs), which contained a germinal center and marginal zone CN, as well as two tumor-associated CNs (LN-CN0 and LN-CN1). While the non-tumor CNs were conserved across both benign and malignant LN samples, the tumor center CN (LN-CN0) was exclusive to LN.met. Notably, the invasive myeloid/CAF-enriched niche CN1, characterized by the enrichment of macrophages, granulocytes, and CAF within the same spatial location, was detected not only in LN.met but also in LN.adjacent and LN.benign, suggesting that LN colonization induces immune remodeling beyond the site of direct tumor involvement.

Despite the clear differences in tissue architecture, we observed that CNs with similar cellular compositions occupied comparable spatial locations relative to the tumor compartment in PTs and LN.met. This resulted in the emergence of distinct CN-CN interaction motifs, termed spatial contexts. Spatial contexts are defined as tissue regions where distinct CNs may interact functionally.^18^ We identified two distinct spatial contexts in PTs and LN.met (Figure S4K), namely the “Tumor Interface” and “Lymphoid Hub.” We also detected a context where both motifs overlapped, which we term the “Tumor-Immune Interface.” This perturbation zone may serve as an immunoregulatory site, where signals from the Tumor Interface interact with the Lymphoid Hub. Prior studies demonstrated that interactions between these functional units can dictate immune responses within tumors.^13^ The identification of this interface suggests that LN colonization may induce similar immune perturbations within this tumor boundary zone across PTs and the TDLNs, which could contribute to metastatic tolerance.

### Cellular reorganization induced by lymph node colonization is marked by the spatial enrichment of immunomodulatory myeloid cells and cancer-associated fibroblasts

**Figure 3.**
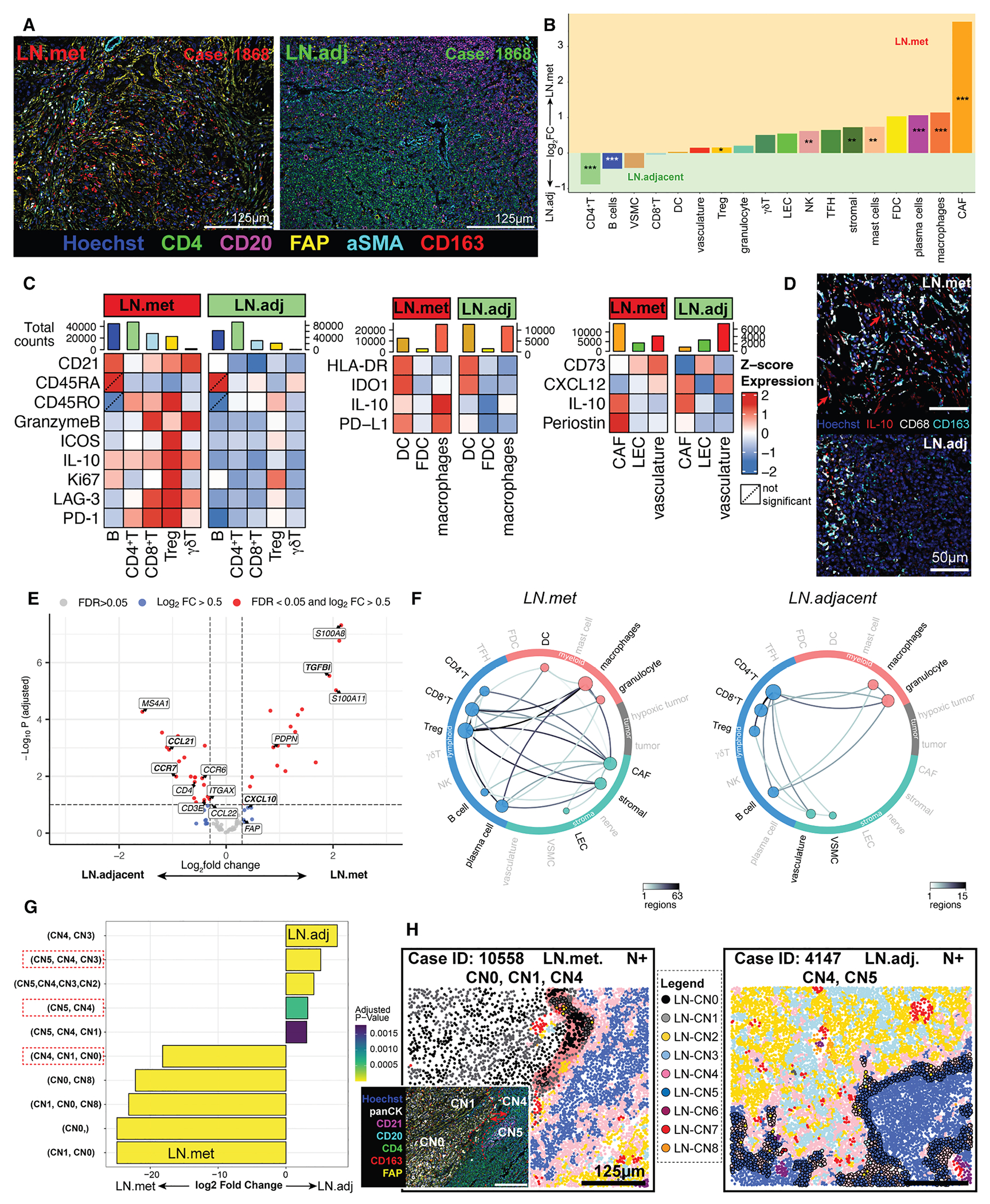
LN.met are characterized by the enrichment of immunomodulatory myeloid cells and CAFs linked to tissue reorganization (A) Representative examples of LN.met and its paired microscopically non-tumor involved adjacent LN region (LN.adj). (B) Waterfall plot highlights the enrichment of cell-types outside the tumor compartment between LN.adj (*n* = 43) and LN.met (*n* = 95). Asterixis indicates significant results from two-sided Wilcoxon test adjusted for multiple testing, *p < 0.05, **p < 0.01, ***p < 0.001. (C) Heatmaps for cell-type specific z-normalized expression of selected functional markers between paired PTs and LN.met. Statistical comparisons between tissue types were made using Wilcoxon test adjusted for multiple testing. (D) Representative examples illustrate macrophage marker expression within LN.met (top) when compared to LN.adj regions (bottom). Red arrows indicate IL-10^high^ macrophages. Scale bar of 50 μm applies to both panels. (E) Volcano-plot depicts results from pseudo-bulk analysis for differentially enriched genes between LN.met (*n* = 29) and LN.adj (*n* = 17) of spatial transcriptomics data. Selected genes implicated in immune cell re-organization within LNs are provided within the graph. (F) Circos plot depicts cellular niche networks characteristic to LN.met (*n* = 95) and LN.adj (*n* = 43). Nodes represent cell types involved in such niche networks and edge weights correspond to the number of unique tissue regions with the corresponding interaction. Node size is proportional to connectedness, as measured by eigenvector centrality. (G) Barplot summarizes log_2_-fold change of the top 10 spatial contexts in LN.met and LN.adjacent with corresponding significance levels. Statistical comparisons of differentially enriched spatial contexts were performed using DESeq2. Selected CN-CN interfaces involving LN-follicles are highlighted in red. (H) Representative examples depict CN-geography maps, where cells within the spatial context involving CN0, CN1, and CN4 are highlighted by black outline and red background layer. Corresponding CODEX image is shown in the bottom left. Scale bar of 125 μm applies to both panels. See also Figures S5–S7.

To identify cellular signatures underlying metastatic tolerance at the LN level, we compared the microenvironments of tumor-involved LN regions and their adjacent microscopically non-tumor-involved counterparts (Figure 3A). In line with prior reports,^5^ we detected a significant enrichment of CAFs, macrophages, and plasma cells within LN.met, whereas CD4^+^ T cells and B cells were more prevalent in LN.adjacent (Figure 3B). Notably, we observed similar compositional shifts in LN.adjacent compared to tumor-free LNs of nodal-positive patients (LN.benign.N+), including an enrichment of CAFs and plasma cells therein (Figures S5A–S5C). This suggests that LN colonization might affect the cellular organization of TDLNs beyond the tumor-infiltrated regions themselves (Figure S5D).

Importantly, T cells within LN.met exhibited the highest levels of T cell dysfunction and cytotoxicity, while Treg presented with elevated levels of Ki67, ICOS, and IL-10 (Figure 3C). These phenotypic changes occurred along a continuum, with T cells in LN.adjacent displaying an intermediate phenotype (Figures S5E–S5G), suggesting a progressive immunosuppressive modulation that extends beyond the tumor colonized LN. In line, we found that myeloid cells within LN.met exhibited an immunomodulatory IL-10^hi^, PD-L1^hi^ phenotype (Figure 3D), reinforcing their role in counteracting effective anti-tumor immune responses.

Similarly, CAFs within LN.met and LN.adjacent were characterized by elevated levels of IL-10 and periostin (Figures S5E and S5F), a matricellular protein linked to ECM remodeling, immune evasion, and cancer dissemination.^19^

Collectively, these findings indicate that LN colonization induces immune and stromal reorganization not only within LN.met but also in LN.adjacent and non-tumor involved LNs of nodal-positive patients, impairing anti-tumor immunity beyond its immediate vicinity. Thus, LN metastasis may represent a broader immunomodulatory event, potentially influencing systemic immune responses in metastatic progression.

### Tumor-derived TGF-β and CXCL10 associate with the spatial re-organization of lymph node microenvironments

To identify molecular drivers of the cellular and phenotypical alterations observed within the metastatic LN microenvironment, we leveraged our orthogonally collected ST data (Figures S6A–S6C). Similar to multiplex proteomic imaging results, we detected a significant enrichment of fibroblasts within LN.met, while B cells and CD4^+^ T cells were relatively depleted (Figure S6D). More importantly, we observed a significant enrichment of *TGFB1* and *CXCL10* within LN.met (Figure 3E), which has been implicated in tumor progression and metas- tasis.^20^ While *CXCL10* expression correlated with effector T cell infiltration (Figures S6E–S6H), we also found that tumor and fibroblast-derived *TGFB1* strongly correlated with the infiltration of activated ICOS^+^ Treg, indicative of an enrichment of immunomodulatory circuits in LN.met limiting T cell effector function (Figures S6E, S6F, and S6I). By contrast, myeloid cell and fibroblast-derived *CCL21* (Figure S6J), which plays a critical role in T cell homing to LNs, was significantly reduced within LN.met. This suggests that the observed reduction of CD4^+^ T cells within LN.met may be partially attributable to impaired T cell homing.^21,22^ In line we detected significant correlations of *CCL21* LN-levels with the degree of naive T cell infiltration (Figures S6E and S6F).

To better understand how these cellular phenotypes affect overall LN organization, we compared LN-specific CNs between tumor-involved and non-tumor-involved LNs. We detected significant enrichments of myeloid/CAF-enriched CNs in LN.met, while LNF-associated CNs were significantly enriched in non-tumor-infested LN regions (Figure S7A). In accordance, we identified distinct cellular niche networks in LN.met, that were characterized by a stronger interaction of macrophages, CAFs, and plasma cells when compared to LN.adjacent, which showed a stronger interaction among CD4^+^ T cells, B cells, and vasculature (Figures 3F and S7B).

Since CNs are defined as distinct functional units within tissues, each with characteristic functional properties, we reasoned that at the intersection of two CNs, those functional properties might be conveyed from one CN to another. To investigate whether CN intersections contribute to the immunosuppressive properties of LN.met, we computed the formation of such spatial contexts (Figure S7C). Unlike tumor-free LNs, where CNs associated with LNF and T cell priming zones (CN3, CN4, CN5) primarily interacted with other follicle-associated CNs, these CNs exhibited substantial intersections with tumor-associated CNs (CN0, CN1) in LN.met (Figures 3G and 3H). Given the role of perifollicular T cell zones (PFTZs) and LNF in T cell priming and B cell activation, their intersection with tumor-associated CNs in LN.met may lead to the functional impairment of these essential immune structures.

Importantly, we also identified the enrichment of such networks involving CAFs, macrophages, and plasma cells, as well as enhanced intersections between the myeloid/CAF-enriched CN1 with the LNF-associated CNs (CN4/CN5) when comparing LN.adjacent to LN.benign (Figures S7D–S7G). These observations substantiate that tumor colonization might induce cellular changes even within nearby non-affected regions, resulting in architectural re-organization in proximity to LNFs.

### LN reorganization is linked to enhanced T cell dysfunction within perifollicular T cell zones and lymph node follicles

**Figure 4.**
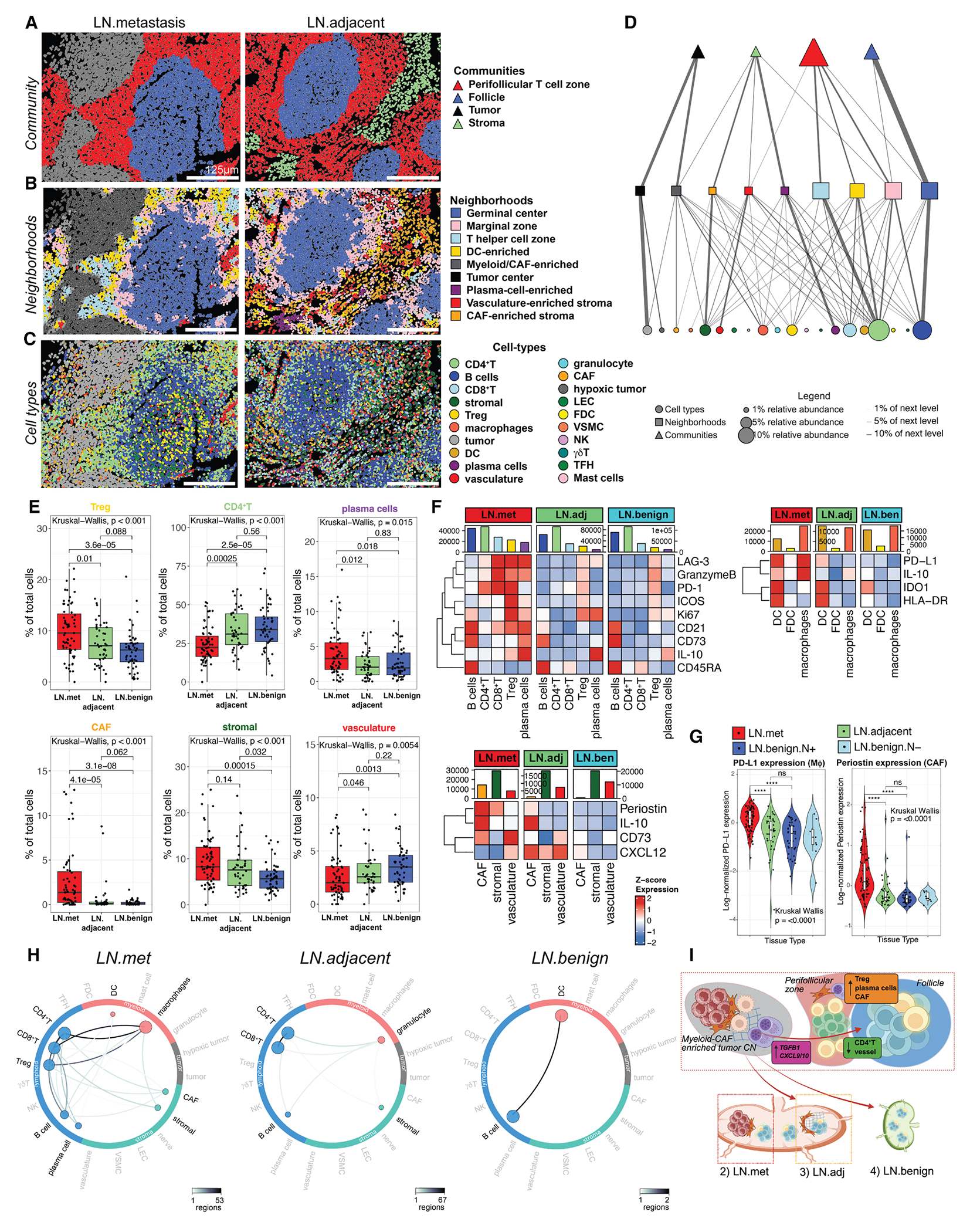
Cellular, organizational and phenotypical changes within perifollicular T cell zones of tumor-involved and non-tumor involved LN regions (A–C) Representation of multiple levels of hierarchical description: community (A) multicellular neighborhood (B) and cell-types (C) between LN.met (left) and LN.adj (right). Scale bar of 125 μm applies to all panels. (D) LN hierarchy graph depicts the multilevel network of the tissue comprised of the different structures. Shapes correspond to structural level; colors represent individual categories as indicated in A-C; size of shapes represents the percentage of tissue; and the size of connected lines represents the overall contribution to the next level of the structure when moving down the graph in increasing tissue structural hierarchy. (E) Boxplots compare the mean abundance of selected cell types within PFTZs of the investigated LN regions (LN.met, *n* = 95; LN.adjacent, *n* = 43; LN.benign, *n* = 49). Center line of boxplots depicts median and box-limits the 25 and 75 percentiles of each condition. Whiskers are defined as 1.5× interquartile range with jitter dots depicting data from each individual region. Statistical comparisons were conducted using two-sided Wilcoxon test corrected for multiple hypothesis testing. Kruskal Wallis test was used for cross-tissue comparisons. (F) Heatmaps compare the mean z-normalized expression of selected functional markers in lymphocytes (left), myeloid cells (top right) and the stromal compartment (bottom right) located within PFTZs between LN.met, LN.adjacent and LN.benign. (G) Combined violin and boxplots compare the mean expression of PD-L1 in macrophages and periostin in CAFs in each LN region (LN.met, *n* = 95; LN.adjacent, *n* = 43; LN.benign.N+, *n* = 49; LN.benign.N−, *n* = 13). Center line of boxplots depicts median and box-limits the 25 and 75 percentiles of each condition. Whiskers are defined as 1.5× interquartile range. Violin width represents the density of observations at a given y-value. Statistical significance was determined using pairwise Wilcoxon test adjusted for multiple-hypothesis testing and Kruskal Wallis test. (H) Circos plots depict the differentially enriched cellular niche networks in LN.met compared to LN.adjacent (left, center) and in LN.benign.N+ (right, center). Size of the nodes indicates the relative contribution of these cell types to the total cell-cell interactions. Thickness of edges represents the abundance of these interactions across all investigated regions. Node size is proportional to connectedness, as measured by eigenvector centrality. (I) Schematic outlining cellular and spatial features of immunomodulation linked to metastatic tolerance (top) across different anatomical LN regions (bottom). See also Figure S8.

To better characterize how the intersection of the myeloid/CAFenriched CN modulates cellular behavior within PFTZs and within LNFs, we identified spatial communities across LN samples.^23^ Such communities are larger organizational units of CNs corresponding to histological LN structures, such as LNF (Figures 4A–4C).

To relate the various levels of spatial organization, we created a hierarchical structure network graph (Figure 4D). Each level of this graph is connected to the next by its major contributors. Using this intuitive formalism, we were able to recapitulate the cellular and organizational patterns and multilevel relationships across LNs. Through this graph structure, we can also observe multilevel relationships between the structures. For example, CAFs were a rare cell subset primarily enriched within the CAF/myeloid-enriched CN that belongs to the larger tumor community. Despite its relative scarcity, CAFs were involved in various cellular and larger level interactions through their contribution to the CAF/myeloid-enriched CN that shows a high diversity of contributing cell types (Figure 4D).

To characterize compositional and phenotypic features of PFTZs and LNFs, we extracted the corresponding community masks and identified all cell types within these masks (Figure S8A), as well as the absolute abundance of LNF communities within the investigated cohort (Figures S8B and S8C). While both PFTZs and LNFs presented with an enrichment of CD4^+^ T cells and vasculature in non-tumor infested LN regions, we detected a relative predominance of Tregs and CAFs within these communities in LN.met (Figures 4E and S8D). In agreement, we detected a gradual enrichment of CD4^+^ and CD8^+^ T cells exhibiting elevated levels of dysfunction, as well as ICOS^hi^ Treg from non-tumor-involved LN regions to LN.met. Similarly, macrophages and DCs presented with a PD-L1^hi^ phenotype within these T and B cell enriched communities of LN.met (Figures 4F, 4G, and S8E). Of note, we also observed enhanced abundances of plasma cells and Ki67^hi^, HLA-DR^hi^ B cells within LNFs of LN.met, indicative of an ongoing germinal center reaction^24^ (Figures S8D and S8E).

These differences in functional marker expression might be derived from the interaction with cells and soluble mediators in the LNF vicinity. Therefore, we computed cell type enrichments as a function of the distance from LNFs. We identified a significant enrichment of CAFs and tumor cells in the vicinity of LNFs of LN.met (Figure S8F). In line, we detected significantly stronger interactions among CAFs, macrophages, and Treg in PFTZs of LN.met (Figure 4H).

These results demonstrate that the intersection of the myeloid/ CAF-enriched CN is linked to cellular, phenotypical, and spatial remodeling of PFTZ and LNFs. These changes not only reflect global tissue average changes but also highlight the spatially defined modulation of cellular behavior and phenotypes within tissue units critical for T cell activation and humoral immune responses (Figure 4I).

This led us to propose a model of gradual immunosuppression as a distance-driven function from tumor-colonized LNs (Figure S8G), where LN.met regions exhibit the most profound compositional changes involving the enrichment of immunosuppressive myeloid cells, CAFs, and activated Tregs, gradually decreasing from LN.adjacent to LN.benign. Importantly, on a tissue architecture level, LN.met show a strong intersection between myeloid/CAF-enriched niches and LNFs, resulting in the accumulation of regulatory myeloid cells that associate with enhanced T cell dysfunction, Treg activation, and B cell maturation. Here, our orthogonal ST data identified tumor-derived *TGFB1* and *CXCL10* as mediators linked to immunological re-organization locally within tumor-involved and regionally within non-tumor involved LNs.

### Spatial co-enrichment of myeloid cells and cancer-associated fibroblasts is specific to nodal involvement in an independent human head and neck squamous cell carcinoma cohort

**Figure 5.**
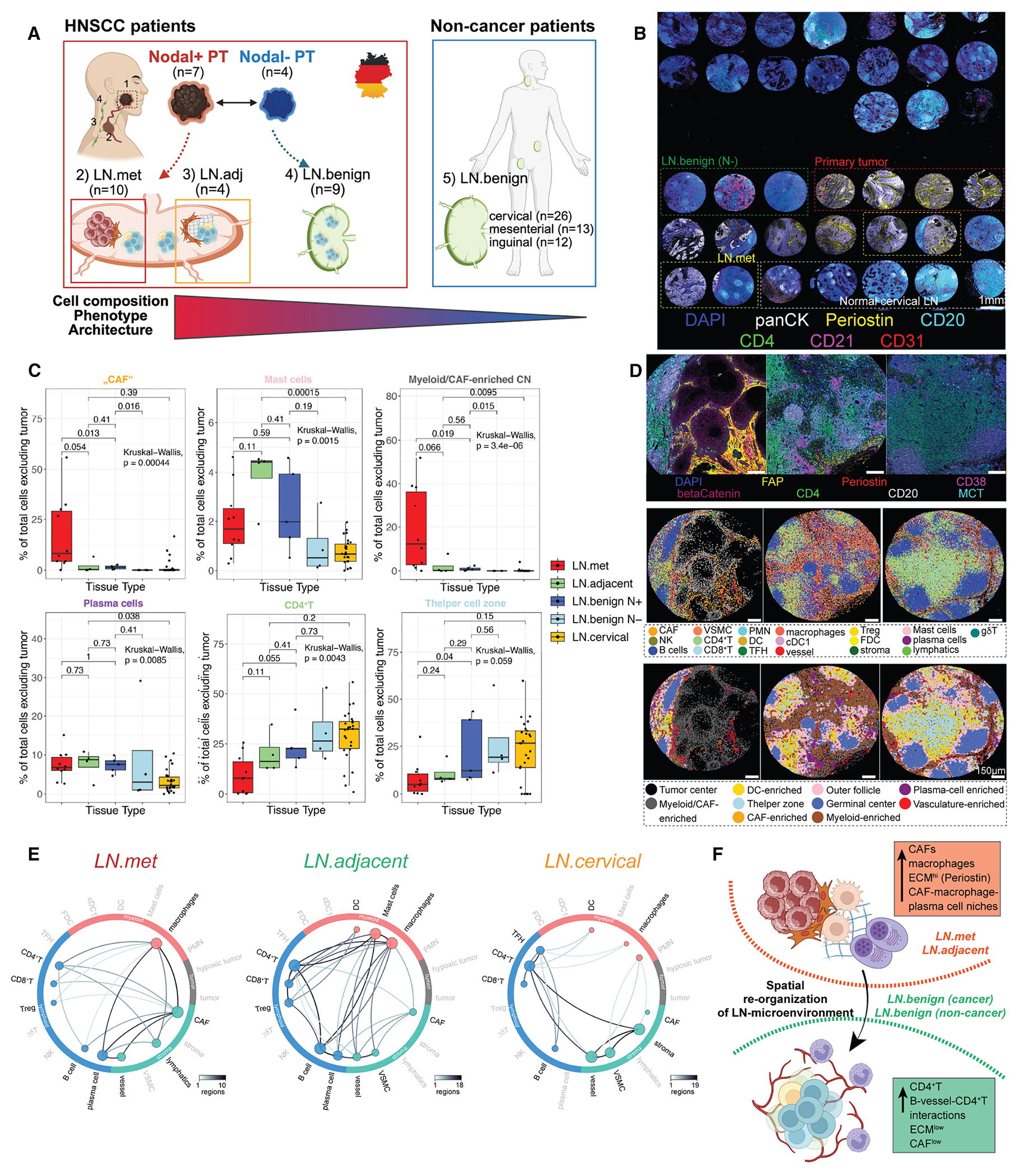
Spatially coordinated interaction of CAFs with myeloid cells within tumor-involved LNs is linked to the reorganization of the LN-microenvironment absent in cervical LNs of non-cancer patients (A) Experimental setup for validation experiments. (B) Overview of the assembled TMA for which representative regions from paired PT and TDLNs were used together with LNs from non-cancer patients. (C) Boxplots compare the relative abundances of major cell types and CNs stratified by the different LN-types (LN.met, *n* = 10; LN.adjacent, *n* = 4; LN.benign.N+, *n* = 5; LN.benign.N−, *n* = 4; LN.cervical, *n* = 26). Center line of boxplots depicts median and box-limits the 25 and 75 percentiles of each condition. Whiskers are defined as 1.5× interquartile range with jitter dots depicting data from each individual region. Statistical significance was determined using Wilcoxon test corrected for multiple hypothesis testing. (D) Representative examples from paired LN.met (left), LN.adjacent (center) and LN.cervical (right). Top row depicts raw immunofluorescence images. Center and bottom rows depict cell-type geography and CN-geography maps with corresponding color-codes. Scale bar of 150 μm applies to all panels. (E) Circos plot depicts cellular niche networks of LN.met regions (left), LN.adjacent (center), and benign LNs of non-cancer patients (right). Nodes represent cell types involved in these characteristic niche networks enriched in the corresponding LN tissues and edge weights correspond to the number of unique tissue regions with the corresponding interaction. Node size is proportional to connectedness, as measured by eigenvector centrality. (F) Schematic summarizes compositional, phenotypical and spatial features of different LN-microenvironments. See also Figures S9 and S10.

To cross-validate our observations, we identified an independent HNSCC cohort comprising paired PTs and TDLNs (Figure 5A; Table S1). We further collected cervical, inguinal, and mesenterial LNs of 27 non-cancer patients (Figure 5A) to compare these spatial signatures to LN organization in healthy individuals. Similar to our discovery cohort, we assembled representative regions of LN.met, LN.adjacent, benign LNs of nodal-positive patients (LN.benign.N+) and nodal-negative patients (LN.benign.N-), and benign LNs from non-cancer patients to construct a dedicated TMA for CODEX multiplex imaging (Figure 5B). The resulting dataset encompassed 2,026,167 cells, which we stringently annotated (Figure S9A).

Comparing the cellular composition of the different LN regions, we found significant enrichments of CAFs and plasma cells in LN.met (Figure 5C), while CD4^+^ T cells were significantly reduced in these LNs (Figure S9B). Aligning with our previous findings, we also observed a stronger periostin expression among these CAFs (Figure S9C).

In accordance with our discovery cohort, we detected 10 distinct LN-CNs, including the previously detected Follicle-CNs, T helper cell zone, and the myeloid/CAF-enriched niche (Figure S9D). Similarly, the myeloid/CAF-enriched CN was predominantly found in LN regions of nodal-positive patients, while T helper cell zones were more frequently detected in LNs of non-cancer patients (Figures 5C, 5D, S9E, S10A, and S10B).

More importantly, differential abundance testing of cellular niche networks highlighted a stronger interaction of CAFs, macrophages, and plasma cells in LN.met, corroborating our previous observations (Figures 5D, 5E, S10C, and S10D). By contrast, cervical LNs from non-cancer patients were devoid of these CAF-macrophage interactions and instead showed enhanced vasculature-CD4^+^ T cell interactions (Figures 5E, S10D, and S10E).

These architectural differences were also detected on the level of CN-CN interfaces: Specifically, we found a significant intersection of the CAF/myeloid-enriched CN with other immune-CNs, including the plasma cell-enriched CN6 and the myeloid cell-enriched CN9 in LNs of nodal-positive patients (Figures S10F–S10H).

Collectively, the tissue atlases of benign and malignant LNs from two HNSCC cohorts and non-cancer patients support a model in which LN.met are characterized by the enrichment and spatial interaction of CAFs, macrophages, and plasma cells. Those cellular interactions occur in a dedicated spatial niche that extends to non-tumor involved LNs of nodal-positive patients but is absent in benign LNs of nodal-negative patients and non-cancer patients. Meanwhile, the intersection of this niche with LNFs and T cell activation zones associates with the enrichment of dysfunctional T cells, activated Treg, and plasma cells, which is accompanied by a reduction of CD4^+^ T cells therein (Figure 5F).

### Metastatic tolerance is linked to spatial and phenotypical macrophage re-organization in an *in-vivo* model of lymph node metastasis

**Figure 6.**
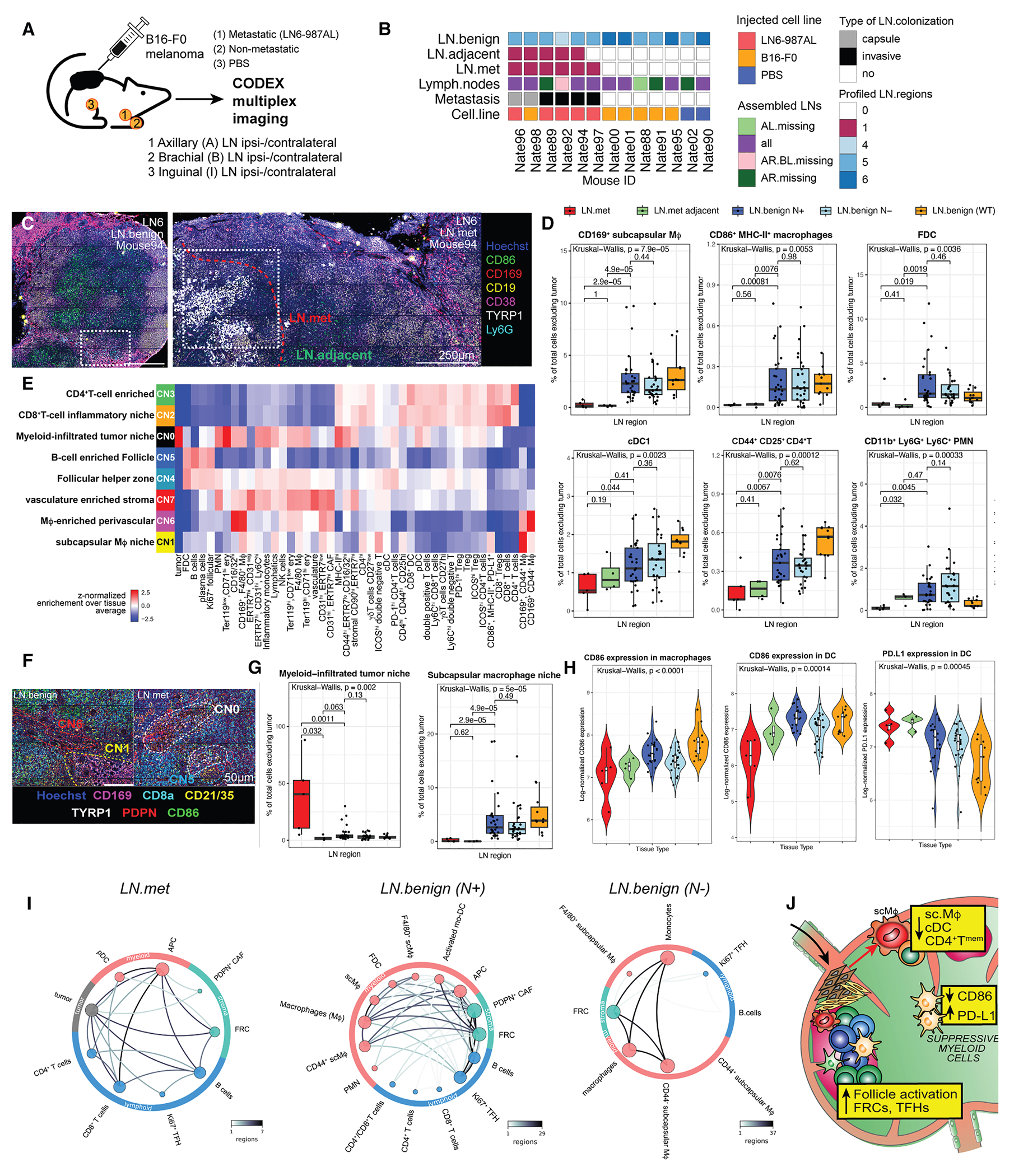
Whole LN profiling in a model of melanoma LN-metastasis identifies a selective reduction of subcapsular macrophages in tumor-infested LNs (A) Experimental approach to study organizational differences of LN architecture in a murine LN metastasis model. (B) Main characteristics of the murine cohort. (C) Representative multiplex images of paired LN.benign (left) or LN.met (right) from mice inoculated with LN6-987AL. Scale bar of 250 μm applies to both panels. (D) Boxplots depict the mean abundances of selected cell types between LN.met (*n* = 5), LN.adjacent (*n* = 4) and LN.benign.N+ (*n* = 29), LN.benign.N− (*n* = 28) and LNs from wildtype mice injected with PBS (*n* = 11). Center line of boxplots depicts median and box-limits the 25 and 75 percentiles of each condition. Whiskers are defined as 1.5× interquartile range with jitter dots depicting data from each individual region. Statistical significance was determined using paired Wilcoxon test adjusted for multiple hypothesis testing and Kruskal Wallis test for cross-tissue comparisons. (E) Heatmap for recurring CNs across all LN-samples with corresponding relative cell type enrichments. (F) Representative examples of magnified regions selected from (C) highlighting phenotypical and spatial features of LN.benign.N+ (left) and LN.met (right). Scale bar of 50 μm applies to both panels. (G) Boxplots depict the mean abundances of the most differentially enriched CNs between the different LN-regions and mouse groups. Center line of boxplots depicts median and box-limits the 25 and 75 percentiles of each condition. Whiskers are defined as 1.5× interquartile range with jitter dots depicting data from each individual region. Statistical significance was determined using paired Wilcoxon test adjusted for multiple hypothesis testing (BH) and Kruskal Wallis test for cross-tissue comparisons. (H) Combined Violin and boxplots highlight differences in the mean expression of CD86 and PD-L1 in selected cell types per investigated LN-region between predefined LN groups. Center line of boxplots depicts median and box-limits the 25 and 75 percentiles of each condition. Whiskers are defined as 1.5× interquartile range. Violin width represents the density of observations at a given yvalue. Statistical significance was determined using Kruskal Wallis test. (I) Circos plot depicts cellular niche networks of LN.met (left), LN.benign.N+ (center) and LNs of mice without LN-metastasis (right). Nodes represent cell types within the corresponding LN-regions and edge weights correspond to the number of unique tissue regions with the corresponding interaction. Node size is proportional to connectedness, as measured by eigenvector centrality. (J) Schematic summarizes key cellular and spatial alterations following LN colonization in B16-F0-melanoma. Abbreviations: scMϕ, subcapsular CD169^+^ macrophages; cDC, conventional dendritic cells; AR, axillary right LN; AL, axillary left LN; BL, brachial left LN. See also Figure S11.

We next sought to dissect whether the phenotypical and organizational changes found in patients with HNSCC would be recapitulated in a controlled system of genetically identical mice. Therefore, we employed an established syngeneic mouse model of melanoma LN metastasis,^5^ where we injected mice with the B16-F0-LN cell line (LN6-987AL), the parental minimally metastatic B16-F0 cell line, or PBS (Figure 6A). While B16-F0 melanoma cells present with faster tumor growth kinetics, the LN6-987AL cell line is characterized by a high propensity to metastasize into LNs^5,25^ (Figure S11A) and increased IFN-responsiveness. This allowed us to study the impact of tumor colonization on spatial immunomodulation within TDLNs (Figure 6B).

We profiled LN microenvironments using a 49-marker CODEX panel on whole-mouse LN sections to spatially resolve all histological LN compartments (Figure S11B). Following preprocessing and cell segmentation, we clustered the resulting 3,425,185 cells to identify 24 major cell types (Figure S11C). As expected, we detected major compositional, phenotypical, and architectural differences between LNs of LN6-987AL, B16F0, and control mice (Figures S11D–S11L).

To study the impact of tumor-seeding in more detail, we further categorized LNs into tumor-infested regions (LN.met), adjacent microscopically tumor-free regions (LN.adjacent), tumor-free LNs from mice with nodal metastasis (LN.benign.N+), and tumor-free LNs from mice without nodal metastasis (LN.benign.N-) compared to LNs from control mice (Figure 6C). Here, we observed a reduction of cDC1 and memory CD4^+^ T cells in tumor-involved LNs of LN6-mice (Figure 6D). Furthermore, we detected that LN.met were particularly devoid of CD169^+^ macrophages, MHC-II^+^ macrophages, and FDCs, which were predominantly located in the subcapsular sinus regions. Subcapsular macrophages are essential for efficient capture of tumor-derived antigens via CD169 that are then relayed to cDC1 for priming of T cells^26,27^ or to B cells and FDCs in LNFs to support humoral anti-tumor responses.^28^

To spatially contextualize our findings, we computed spatially recurring CNs (Figure 6E). Thereby, we identified 7 CNs reflecting murine LN architecture, including LNF-CNs, subcapsular macrophage niches, and T cell rich CNs. In line with the previous observations from patients with HNSCC, we identified a neighborhood characterized by the infiltration of myeloid cells, CD90^+^ CAFs, and tumor cells. This myeloid-infiltrated tumor niche was predominantly found in LN.met but mostly absent in LNs of nodal-negative mice (Figures 6F and 6G). In addition, we detected a striking reduction of subcapsular macrophage niches in LN.met, suggesting a subversion of myeloid-driven antigen priming processes to support anti-tumor immunity toward a tumor supporting myeloid environment (Figure 6G). In accordance, macrophages and DC from LN.met presented with an anti-inflammatory phenotype, including lower levels of CD86 and MHC-II, but enhanced PD-L1 expression (Figures 6F–6H).

More importantly, we observed distinct niche networks between the different LN regions reflective of the spatial immunomodulation during metastatic tolerance: While LN.met showed significant interactions of myeloid antigen-presenting cells (APCs) with CD8^+^ and CD4^+^ T cells, as well as PDPN^+^ CAFs (Figure 6I, left), this interaction was not found in LN.benign.N- (Figure 6I, right). On the other hand, we identified a strong interaction between myeloid APCs, CAFs, subcapsular macrophages, follicular T helper cells (TFH), and B cells in LN.benign.N+, implicating higher levels of immune activation within these LNs with early signs of immune dysfunction (Figure 6I, center). Those interactions were reflected in higher order spatial contexts, where LN.met showed intersections of Follicle-associated CNs with the myeloid-enriched tumor niche CN0 (Figures S11L and S11M, left), while this intersection was not found in other LN regions. Instead, we observed that CN0 intersected with the CD4^+^ T-helper zone and CD8^+^ T cell activation zone in LN.benign.N+, indicating an early impairment of these critical niches in tumor-free LNs (Figure S11M, center left). Critically, this spatial context was neither found amongst LNs of tumor-free mice nor LN.benign.N- (Figure S11M, right).

Collectively, these data suggest that LN colonization initiates a sequence of events that includes the displacement of subcapsular macrophages and cDC1, the subversion of myeloid cells to acquire an immunoregulatory PD-L1^hi^, CD86^low^ phenotype, and the formation of a myeloid-CAF enriched tumor neighborhood. The formation of this distinct niche is in turn linked to characteristic niche networks that – like for human HNSCC – subvert T and B cell enriched niches critical for anti-tumor immune responses (Figure 6J).

### Enhanced tumor-immune interactions in primary tumors predict lymph node colonization

**Figure 7.**
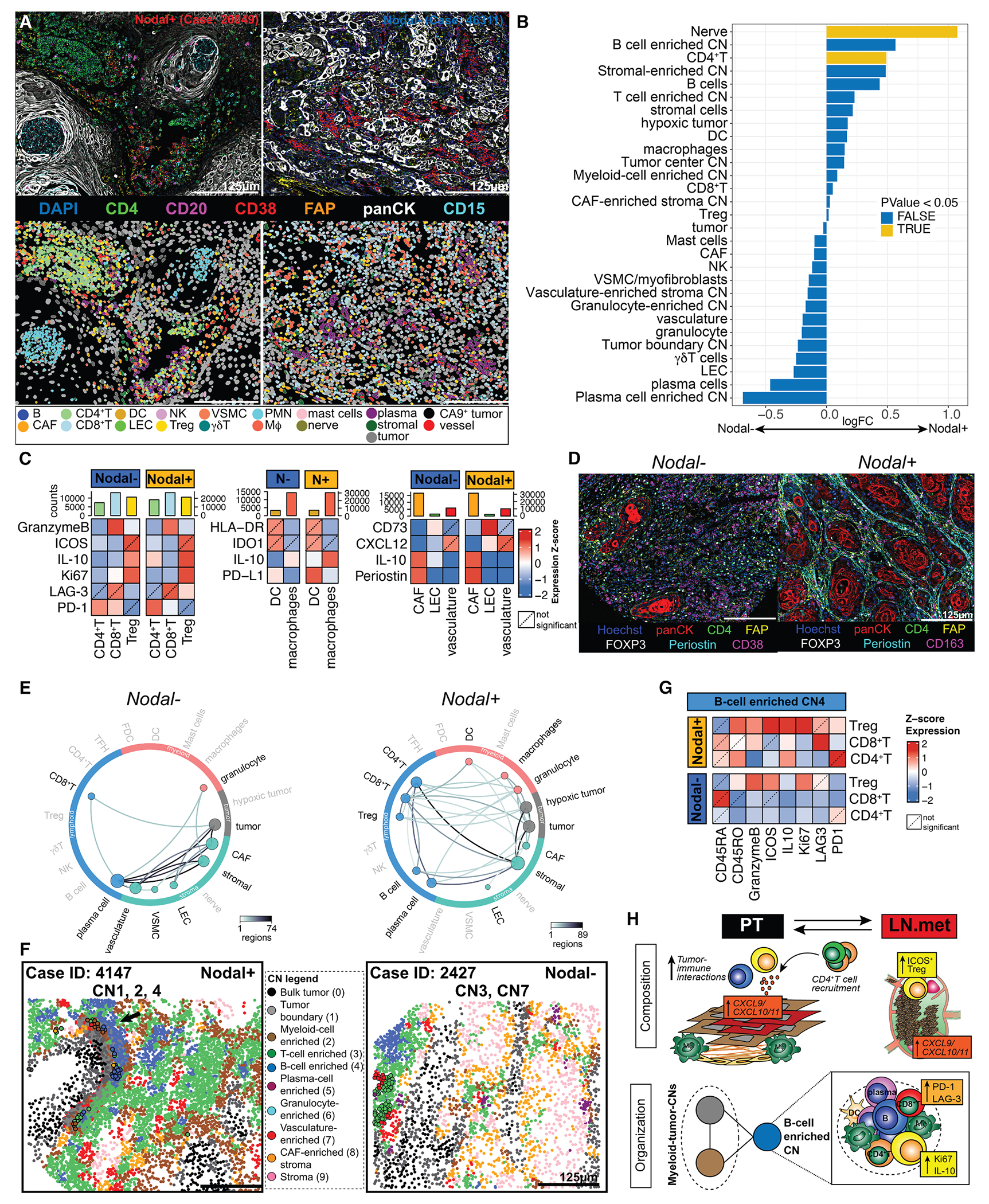
Enhanced tumor-immune interactions are reflective of nodal disease in primary HNSCC (A) Compositional features of nodal-positive and nodal-negative PTs (top) with corresponding cell type geography maps (bottom). Scale bar of 125 μm applies to all panels. (B) Waterfall plot highlights relative enrichments of all identified cell-types and CNs between nodal-positive (*n* = 125) and nodal-negative PT regions (*n* = 62). (C) Heatmaps compare the mean z-normalized expression of selected functional markers stratified by tissue compartment between nodal-negative and nodal-positive PT-samples. Comparisons of marker expression levels within selected cell types between tissues were performed using the Wilcoxon test adjusted for multiple hypothesis testing. (D) Multiplex imaging highlights key phenotypical features of nodal-negative and nodal-positive PTs. Scale bar of 125 μm applies to both panels. (E) Circos plot depicts characteristic cell-cell interactions in PTs with (right) or without (left) nodal disease. Nodes represent cell types within cancer border-associated niches, and edge weights correspond to the number of unique regions with the corresponding interaction. Node size is proportional to connectedness, as measured by eigenvector centrality. (F) CN geography map, with cells involved in the intersection of CN1, CN2, and CN4 (left) highlighted with a black outline (left). Representative CN geography map, where cells involved in the intersection of CN3 and CN7 are highlighted by a black outline (right). Scale bar of 125 μm applies to both panels. (G) Heatmap highlights the z-normalized expression of selected functional markers across T cells within the B cell enriched CN4 between nodal-positive and nodal-negative cases. Comparisons of marker expression levels between nodal status were performed using the Wilcoxon test adjusted for multiple hypothesis testing. (H) Schematic summarizes key cellular and architectural features of nodal-positive PTs and their relation to paired LN-metastases. See also Figures S12–S15.

We finally investigated spatial signatures at the PT-level indicative of LN colonization. To this end, we stratified all PTs from our HNSCC discovery cohort by nodal disease (Figure 7A). We observed a significant enrichment of CD4^+^ T cells within nodal-positive patients, whereas nodal-negative patients were enriched in plasma cell niches (Figure 7B). We also observed higher levels of PD-1 expression within T cells, while Treg exhibited higher levels of Ki67 and IL-10 expression in nodal-positive PTs. In line, we detected elevated IL-10 and PD-L1 levels within DC and macrophages of nodal-positive tumors, while CAFs exhibited higher expression of IL-10 and periostin (Figures 7C and 7D). These compositional and phenotypical changes were observed within the entire cohort and after adjusting for tumor stage (Figures S12A–S12D). More notably, PTs of nodal-positive patients were enriched in niche networks involving the interaction of hypoxic tumor cells with macrophages, Treg, and CD8^+^ T cells (Figure 7E), whereas in nodal-negative PTs we found a stronger interaction among stromal cells and plasma cells (Figures 7E, S12E, and S12F).

To delineate molecular cues involved in the establishment of these tumor-immune networks, we employed our ST data (Figure S13A). Similar to our CODEX dataset, we detected an enhanced plasma cell infiltration in PTs of nodal-negative patients (Figures S13B and S13C). More importantly, we observed an enrichment of interferon-γ-related chemokines, including epithelial-cell derived *CXCL9-CXCL11* and *VEGFC* (Figures S13D–S13G) within nodal-positive tumors. Expression of *VEGFC* and *CXCL10* has previously been linked to a higher propensity for metastasis in solid cancers.^20,29^ Furthermore, CXCL9/10 signaling has been implicated in the recruitment of activated effector T cells.^30–32^ Given the synergistic nature of cytokine signaling circuits, we identified niches of cytokines that might act in concert to orchestrate T cell behavior (Figure S13H). We found that *IL-10*-rich cytokine niches were located predominantly in stromal areas, while *CXCL9-11* were enriched in tumor cell proximity (Figure S13I), indicative of T cell recruitment toward tumor nests, supporting the enhanced interaction of Treg, CD8^+^ T cells, macrophages, and tumor cells in nodal-positive patients.

These cellular changes were accompanied by distinct spatial rules underlying PT architecture: Specifically, we found a 2-chain motif including the interaction of the B cell enriched CN4 with the tumor boundary CN1 among nodal-positive PTs (Figure S13J), which was recapitulated on the level of spatial contexts through an enhanced intersection of CN1 and CN4 (Figures 7F and S13K). By contrast, nodal-negative PTs were characterized by higher-order motifs involving instances of CN4 with the stroma-CN9 (Figure S13K).

We reasoned that the intersection of CN1 with CN4 might modulate T cell phenotypes within CN4 of nodal-positive patients. In line, we observed higher expression of PD-1, LAG-3, and Granzyme B among CD8^+^ T cells, while Treg exhibited enhanced expression of Ki67 and ICOS within CN4 of nodal-positive PTs (Figure 7G).

Collectively, these data demonstrate that PTs of nodal-positive patients exhibit enhanced tumor-immune interactions. These enhanced tumor-immune interactions are linked to elevated *CXCL9/10* levels that associate with a stronger intersection of the tumor boundary CN1 with the B cell enriched CN4. Similar to LN.met this spatial context correlated with T cell dysfunction and Treg activation within B-cell enriched CNs, implicating a shared spatial context across PTs and LNs linked to metastatic tolerance (Figure 7H).

Notably, the compositional and spatial signatures associated with nodal disease were distinct from signatures linked to the risk for DR which is considered a critical feature of adverse patient outcomes (Figures S14A and S14B): Specifically, we observed that the abundance of CD8^+^ T cells, plasma cell enriched CNs, and B cell enriched neighborhoods in PTs associated with the absence of DR, whereas the enrichment of stromal neighborhoods and granulocytes was linked to DR (Figures S14C–S14G). In accordance, we found that spatial contexts involving the intersection of T and B cell enriched CNs, indicative of local TLS formation, were enriched in patients without DR (Figures S14H–S14K), which is in line with previous data demonstrating the favorable prognostic role of TLS (Figures S15A–S15I) in advanced solid cancers.^33–35^

## DISCUSSION

LN colonization is a critical driver of metastatic progression,^11^ inducing tumor-specific immune tolerance that facilitates metastatic spread.^5^ While previous studies highlighted the role of tumor-specific Tregs in this process, our multi-modal spatial analysis reveals a more complex, coordinated reorganization of the LN microenvironment.

We demonstrate that LN metastasis is not a passive event but an active remodeling process characterized by the enrichment of macrophages and CAFs with an immunomodulatory phenotype that might be acquired as a result of elevated *TGFB1* and *CXCL10* signaling within the tumor compartment. *CXCL10* signaling is classically associated with beneficial outcomes, but it has also been correlated with the expression of immunoregulatory molecules such as PD-L1 and IDO1.^36–38^ This suggests that LN colonization does not simply reflect passive tumor spread but represents an active immunosuppressive process that modifies the surrounding TME to facilitate immune evasion. In line, fibroblast-derived TGF-β has been implicated in macrophage polarization toward an anti-inflammatory phenotype, supporting the formation of an immunosuppressive LN-microenvironment.^39,40^ Notably, elevated *TGFB1* levels also correlated with enhanced infiltration by ICOS^+^ Tregs, a specialized Treg subset^41^ that were previously shown to impair cDC1 in a spatially coordinated manner within TDLNs, resulting in impaired CD8^+^ T cell responses.^42,43^

We further show that immunomodulatory macrophages and CAFs formed a distinct niche within LN microenvironments that spatially intersects with PFTZs and LNFs, which is linked to T cell dysfunction therein. CAFs can actively recruit immunosuppressive myeloid cells via the secretion of soluble mediators such as TGF-β or IL-1β,^44^ supporting the concept that the LN microenvironment is not only permissive but actively remodeled to favor immunosuppression. Here, therapeutic targeting of TGF-β might reverse the detrimental interaction of myeloid/CAF niches and ICOS^+^ Treg. Previous reports showed that the combination of PD-L1 and TGF-β targeting therapies is capable of reversing T cell exclusion and re-instating effective anti-tumor immune responses.^40,45,46^ As a result, our observations emphasize the potential of neoadjuvant regimens targeting the PD-1/PD-L1 axis in combination with immunomodulatory agents to reverse myeloid cell repolarization and CAF-mediated immune evasion within TDLNs, thereby re-establishing an effective immune response within tumor-involved and tumor-free LNs.

Our murine LN metastasis model complements these findings by showing that metastatic tolerance involves both the recruitment of suppressive niches and the active dismantling of stimulatory compartments, such as subcapsular macrophages and cDC1s that are involved in antigen-cross presentation to CD8^+^ T cells.^47,48^ This dual mechanism - depletion of APCs alongside the enrichment of inhibitory myeloid-CAF interactions - suggests a systemic propagation of tolerance.

The striking similarity in cellular organization between PTs and paired TDLNs with enhanced intersections of myeloid/CAF niches with nearby B cell niches further supports the concept of a coordinated, tumor-induced remodeling process spanning multiple anatomic sites. We speculate that the spatial intersection of the B cell-enriched-CN with myeloid/CAF niches might be attributable to enhanced *CXCL9/10* signaling. The CXCL9-11/CXCR3-axis is involved in T cell recruitment to the tumor site.^31,49,50^ However, our findings suggest that in nodal-positive tumors, CXCL9/10-mediated recruitment does not equate to functional anti-tumor immunity. In line, oncogenic CXCL10 signaling has been implicated in the risk of metastasis formation by promoting tumor cell migration,^20^ emphasizing its role in reshaping the TME for metastatic tolerance.

Collectively, our study identified key spatial signatures of immunomodulation within PTs and paired TDLNs that underly metastatic tolerance. By demonstrating that LN colonization is not simply a passive indicator of metastasis but an active site of immunosuppression, our findings emphasize the role of TDLN as therapeutic targets. These results provide a foundation for immune-monitoring strategies and immunotherapeutic interventions aimed at reversing metastatic tolerance in solid cancers.

### Limitations of the study

One limitation of our study is that it is based on spatial associations and phenotypic characterization, which provide strong evidence for immune remodeling but do not establish direct mechanistic links. Our data support that targeting TGF-β and PD-L1 may reverse immunosuppression, but no direct evidence is provided that the inhibition of these pathways restores immune function in LN metastasis, which requires experimental validation in patient-derived or preclinical models. Our data indicate an increased activation of TFH and B cells within the LNF of LN metastases. Whether this represents a compensatory mechanism or an ineffective anti-tumor reaction remains an open question.

The use of TMAs for spatial multiplex imaging techniques introduces potential bias, as these 1-1.5mm cores are enriched for invasive tumor areas and may not fully capture broader LN architecture. Limited access to LN.benign samples in nodalnegative patients for spatial transcriptomic imaging constrained statistical comparisons of these LNs between patient groups, leaving open questions about whether certain immune changes predate metastasis.

A key unresolved question is whether LN.met actively initiates systemic immunosuppression or whether pre-existing immune dysfunction predisposes certain patients to nodal involvement. Our study captures immune remodeling at a single time point, making it difficult to determine whether T cell dysfunction and myeloid repolarization precede metastasis or develop as a consequence. Addressing this issue would require longitudinal LN sampling before and after tumor spread, for which the current results are predictive justification.

## RESOURCE AVAILABILITY

### Lead contact

Further information and requests for resources should be directed to and will be fulfilled by the lead contact, Garry P. Nolan (gnolan@stanford.edu).

### Materials availability

All materials used in this study are commercially available, as specified in the key resources table and Table S2.

## STAR★METHODS

### EXPERIMENTAL MODEL AND STUDY PARTICIPANT DETAILS

#### Study design for HNSCC discovery cohort

For the HNSCC discovery cohort formalin-fixed paraffin-embedded (FFPE) tissue blocks from 78 patients diagnosed with head and neck squamous cell carcinoma (HNSCC) were collected by the Stanford Department of Pathology. Each patient without evidence of LN metastasis (pathological N stage of N0), underwent elective LN dissection, from which all those lymph nodes that likely constituted TDLNs were collected for construction of the TME. For each patient with LN metastases (pathological N stage of N1-N3) tissue was collected from metastatic LNs, adjacent microscopically tumor-free regions of those LNs and benign LNs. Malignancy diagnosis was confirmed by a board-certified surgical pathologist (C.K.). Human HNSCC tissue collection was approved by the Stanford Research Compliance Office and was performed according to institutional guidelines under Stanford IRB protocol 38502, “Modeling the Role of Lymph Node Metastases in Tumor-Mediated Immunosuppression”. Analysis of the anonymized patient data was conducted in accordance with the guidelines of the Declaration of Helsinki. Clinical metadata for the HNSCC discovery cohort can be found in Table S3.

#### Study design for HNSCC validation cohort

For the HNSCC validation cohort formalin-fixed paraffin-embedded (FFPE) tissue blocks from 6 patients diagnosed with head and neck squamous cell carcinoma (HNSCC) were collected from the Institute of Pathology of the University Medical Center Mainz. Similar to our HNSCC discovery cohort, we included patients diagnosed with pT3 oropharyngeal squamous cell carcinoma that did (n=4) or did not have (n=2) nodal-disease at the time of initial diagnosis. These n=6 patients were treated at the University Medical Center Mainz between 2011 and 2023 and received either adjuvant radiotherapy or combined adjuvant radiochemotherapy following initial neck dissection. Primary tumors, LN metastasis and non-tumor involved LNs were collected as part of initial surgery and neck dissection that was performed prior to any systemic or radiotherapeutic interventions (Table S4).

#### Expansion cohort of benign lymph nodes from non-cancer patients

We identified a cohort of 27 patients that were diagnosed with conditions other than lymphoproliferative, hematological or neoplastic disease and underwent elective surgery at the University Medical Center Mainz between 2015 and 2020 during which LNs were incidentally removed. Such surgeries included thyroidectomy for struma nodosa, colectomy for diverticulitis, or herniotomy for inguinal hernias. Critically, patients were not diagnosed with any neoplastic disease 2 years prior or after surgery. Of these 27 patients we collected 16 cervical lymph nodes (LN.cervical), 5 inguinal lymph nodes and 6 mesenterial lymph nodes, which served as a healthy control in our experiments (Table S5).

Human HNSCC validation cohort and LN expansion cohort tissue collection was reviewed and approved by local ethics committee (Ethik-Kommission der Landesärztekammer Rheinland-Pfalz, No: 837.150.14 (9389-F)). The patients/participants provided their written informed consent to participate in this study. Analysis of the anonymized patient data was conducted in accordance with the guidelines of the Declaration of Helsinki.

#### Mice

C57BL/6J (Stock # 000664) mice were acquired from Jackson Labs (JAX) and housed in the facility at Stanford University. Genetic mice were also bred within this facility. All animal studies were performed in accordance with the Stanford University Institutional Animal Care and Use Committee under protocol APLAC-17466. All mice were housed in an American Association for the Accreditation of Laboratory Animal Care–accredited animal facility and maintained in specific pathogen-free conditions. All studies were performed in animals between 8 to 10 weeks of age, unless otherwise specified. All tumor transplant studies were performed in female mice, unless otherwise specified. Co-housing of different conditions was performed for all experiments.

#### B16-F0 melanoma cell lines

The B16-F0 tumor lines were acquired form ATCC (CRL-6322 and CRL-6475) and grown in Dulbecco’s Modified Eagle Medium (DMEM) supplemented with 8mM L-glutamine, 10% Fetal Bovine Serum (FBS), and 1% Penicillin Streptomycin. Generation of melanoma LN cell lines has been described previously.^5^ Briefly, tumor cells were washed with phosphate buffered saline (PBS) and dissociated from tissue culture plastic with StemPro Accutase (Thermo, A1110501). Cell suspensions of 2×10^5^ cells in phenol red free DMEM were injected into the subcutaneous region of the left flank of seven- to nine-week-old C57BL/6J female mice (Jackson, 000664) following removal of fur with surgical clippers. Tumors were allowed to grow for 33 days or until mice were moribund, at which point mice were euthanized. Inguinal, brachial, and axillary LNs were harvested and mechanically dissociated on 100μm cell strainers. Strainers were washed with DMEM, and cells were resuspended and plated following centrifugation. Tumor cells were expanded *ex vivo* into cell lines and cultured for three to six passages prior to injection into naïve recipients. We selected a cell-line with a high incidence of lymph node metastasis (LN6-987)^5^ based on gross presence or absence of metastasis in inguinal, brachial and axillary lymph nodes.

### METHOD DETAILS

#### Construction of tissue microarrays for the HNSCC discovery cohort

Tissue microarrays (TMAs) with 1mm diameter cores were assembled and digitized using a Leica Aperio whole slide scanner at 40x magnification. The TMA was sectioned at 3 μm thickness onto either SuperFrost Plus microscopy slides (for spatial transcriptomics) or 25x25mm square glass coverslips (for CODEX multiplex imaging). Square glass coverslips (Electron Microscopy Sciences) were pre-treated with Vectabond (Vector Labs) according to the manufacturer’s instructions. Briefly, coverslips were immersed in 100% acetone for 5 min and then incubated in a solution of 2.5 mL Vectabond and 125 mL 100% acetone in a glass beaker for 30 min. Coverslips were subsequently washed in 100% acetone for 30 s and air-dried, baked at 70° C for 1 h, and stored at room temperature until further processing.

#### Generation of CODEX DNA-conjugated antibodies

Buffers and solutions for CODEX antibody conjugation and multiplex imaging were prepared as previously described.^51^ For CODEX antibody conjugation we used a previously described protocol.^13^ Maleimide-modified short DNA oligonucleotides were purchased from Biomers.net. Conjugations were performed at a 2:1 weight/weight ratio of oligonucleotide to antibody with at least 50 μg of antibody per reaction. All centrifugation steps were at 12,000 g for 8 min, unless otherwise specified. Purified, carrier-free antibodies (for details on clones and manufacturers see Table S2) were concentrated on 50-kDa filters, and sulfhydryl groups were activated using a mixture of 2.5 mM TCEP and 2.5 mM EDTA in PBS, pH 7.0, for 30 min at room temperature. After washing the antibody with buffer C, activated oligonucleotides were resuspended in buffer C containing NaCl at a final concentration of 400 mM. Oligonucleotides were then added to the antibody and incubated for 2 h at room temperature. The conjugated antibody was washed by resuspending and spinning down three times in PBS containing 900 mM NaCl. It was then eluted by centrifugation at 3,000 g for 2 min in PBS-based antibody stabilizer (Thermo Fisher) containing 0.5 M NaCl, 5 mM EDTA, and 0.02% w/v NaN_3_ (Sigma), and stored at 4° C.

#### CODEX antibody screening, validation and titration

Conjugated CODEX antibodies were tested and titrated in low-plex fluorescence assays and the resulting expression patterns, and signal-to-noise-ratios were cross-verified with manual immunohistochemistry (IHC) results (Figure S2). Antibody-oligonucleotide conjugates were tested together in a single CODEX multicycle and the optimal dilution, exposure time and appropriate imaging cycle was determined for each conjugate (Table S2A specifies CODEX multi-cycle reaction and image acquisition details). All validation steps were performed under the supervision of a pathologist (C.K.) and confirmed using online databases, including The Human Protein Atlas and Pathology Outlines, as well as relevant published literature.

#### CODEX FFPE tissue staining and fixation

Coverslips and or microscope slides were handled using Dumont coverslip forceps (Fine Science Tools). For deparaffinization, coverslips were baked at 70° C for at least 1 hour, followed by immersion in fresh xylene for 30 minutes. Sections were rehydrated through descending ethanol concentrations (100% twice, 95% twice, 80%, 70%, and doubly distilled water (ddH_2_O) twice; each step for 3 minutes). Heat-induced epitope retrieval was performed in a Lab Vision PT module (Thermo Fisher) using target retrieval solution at either pH 9 (DAKO) or pH 6 (Akoya Biosciences) at 97° C for 10 minutes. After cooling to room temperature for 30 minutes, coverslips were washed for 10 minutes in 1x TBS IHC wash buffer with Tween 20 (Cell Marque).

To minimize non-specific binding, coverslips were incubated with 100 μL of blocking buffer containing S2 buffer supplemented with B1 (1:20), B2 (1:20), B3 (1:20), and BC4 (1:15) for 1 hour at room temperature, as previously described.^13^ For each coverslip, DNA-conjugated antibodies were added to 50 μL of blocking buffer on a 50-kDa filter unit, concentrated by centrifugation at 12,000 g for 8 minutes, and resuspended in blocking buffer to a final volume of 100 μl. This antibody cocktail was then added to the coverslip and staining was performed in a sealed humidity chamber overnight at 4° C on a shaker. After staining, coverslips were washed for 4 minutes in S2, then fixed in S4 buffer containing 1.6% paraformaldehyde for 10 minutes, followed by three washes in PBS. The coverslips were subsequently incubated in 100% methanol on ice for 5 minutes, followed by three additional PBS washes.

Fresh BS3 fixative was prepared immediately before final fixation by thawing and diluting a 15 μL aliquot of BS3 in 1 mL PBS. Final fixation was performed at room temperature for 20 minutes, followed by three washes in PBS. Coverslips or microscope slides were then stored in S4 buffer at 4° C for up to two weeks or processed immediately for imaging.

#### CODEX multi-cycle reaction and image acquisition

Following CODEX staining coverslips were removed from storage buffer S4, rinsed in ddH_2_O to remove salt residues and mounted onto custom-made CODEX acrylic plates (Bayview Plastic Solutions) using coverslip mounting gaskets (Qintay), creating a well in the acrylic plate above the tissue section for liquid storage and exchange and a total imaging area of 24x24mm. Tissue was stained with Hoechst nuclear stain at a dilution of 1:600 in H2 buffer for 1 min, followed by three washes with H2 buffer. The CODEX acrylic plate was mounted onto a custom-designed plate holder and secured onto the stage of a BZ-X710 inverted fluorescence microscope with a CFI Plan Apo λ 20×/0.75 objective (Nikon) using custom adapters. For each core an area of 1x1 fields of view (0.6x0.6mm) and an adequate number of *z* planes (10–14) required to capture the best focal plane across the imaging area were selected. Fluorescent oligonucleotides (concentration: 200 nM) were aliquoted into Corning black 96-well plates in 250 μL H2 buffer containing Hoechst nuclear stain (1:600) and 0.5 mg/ml sheared salmon sperm DNA. Black plates were sealed with aluminum sealing film (VWR Scientific) and kept at room temperature during the multi-cycle reaction. Multicycle imaging was performed using a CODEX microfluidics device and CODEX driver software v1.29.0.1 (Akoya Biosciences). Exposure times and assignment of markers to imaging cycles and fluorescent channels are provided in Table S2. After completion of multicycle imaging, coverslips were stained with hematoxylin–eosin, and the same areas were imaged in brightfield mode.

#### Image processing

Raw imaging data were processed using the RAPID uploader for image stitching, drift compensation, deconvolution, and cycle concatenation.^14^ After deconvolution, best focal plane selection, lateral drift compensation, stitching of individual images and background subtraction, processed images were concatenated to hyperstacks. Individual nuclei were segmented on the basis of the Hoechst stain (cycle 1), derived nuclear masks were dilated, and cellular marker expression levels were quantified using a modified version of the Mask region-convolutional neural network-based CellSeg,^52^ that incorporates lateral marker spillover compensation. This resulted in the segmentation of a total of 1,810,130 cells for the HNSCC discovery cohort and 2,186,167 cells for the combined HNSCC validation and normal LN cohorts. Both the RAPIDS image processing software and Segmenter software can be downloaded from our GitHub site (https://github.com/nolanlab/CODEX), and the CellVisionSegmenter software can be accessed at https://github.com/michaellee1/CellSeg. After the upload, images were evaluated for specific signal. Any markers that produced an untenable pattern or a low signal-to-noise ratio were excluded from the ensuing analysis. Uploaded images were visualized in ImageJ (https://imagej.nih.gov/ij/).

#### CODEX imaging data analysis

##### Cell-type labels and cell type identification

Cell type identification was performed following previously established methods.^23,51^ Briefly, non-nucleated cells were removed by thresholding the intensity of the nuclear markers Hoechst and DRAQ5, and cells from tissue areas of low image quality were excluded. We also removed tissues that were not classified as primary tumors, lymph node metastases, benign lymph nodes, or adjacent lymph node regions, resulting in a final dataset comprising 1,556,569 cells for the HNSCC discovery cohort and 2,026,167 cells for the combined HNSCC validation and normal LN cohort. Marker expression levels were compensated for lateral spillover and subsequently z-normalized per imaging run for further analysis.

To define cell populations, the data were over-clustered using a Leiden-based clustering approach in the *scanpy* Python package. Cell types were assigned to each cluster based on average cluster protein expression and spatial location within the tissue. Clusters were mapped onto the tissue for manual verification, and impure clusters were either split or re-clustered, with assignments cross-checked against original fluorescent images. Following the identification of major cell types, cell subtypes were further defined by re-clustering within the corresponding major cell type clusters, using phenotype-specific markers: These included CD45, CD3, CD4, CD8, CD25, FOXP3, TCRγδ, CD56, CD57, PD1 for T-cell lineage subsets (CD4T, CD8T, Treg, γδT, NK, TFH); CD45, CD20, CD21, CD38, CD138 for B-cell lineage subsets (B, plasma cells); CD45, CD11b, CD16, CD68, CD163, CD206, CD15, MMP9, CD11c, CD1, HLA-DR, MCT for myeloid cell subsets (macrophages, DC, FDC, granulocytes, mast cells); CD73, CD90, Vimentin, CollagenIV, PDGFRB, FAP, αSMA, CD31, CD34 and Podoplanin for stromal cell types (vasculature, CAF, VSMC, LEC and other structural stromal cells), as well as Cytokeratin, EGFR and CA9 for epithelial lineage (tumor, hypoxic tumor).

##### Cellular neighborhood identification

Neighborhood analysis was performed as previously described.^13^ A window size of 15 nearest neighbors was taken across the tissue cell-type maps. Given the biological differences between LNs and primary tumor samples we generated cellular neighborhoods (CNs) separately for primary tumors and all lymph node tissues. Cells of each type were quantified within each window, and then these vectors were clustered into commonly composed neighborhoods.

To determine the optimal number of cellular neighborhoods, we used k-means clustering with an elbow plot strategy and over-clustering. For over-clustering, 20 clusters were initially generated, which were then mapped back to the tissue for evaluation of cell-type enrichments and overall structural organization. Investigators (M.H., M.A.B.) were blinded to clinical metadata information during single-cell spatial data analysis to avoid classification bias.

##### Cell-type specific marker expression

To assess cell-type-specific marker expression, we filtered the dataset for the cell types of interest and used z-normalized marker expression to compare mean expression between conditions. For example, we compared CD8^+^ T cell expression between nodal-positive and nodal-negative primary tumor samples using a Wilcoxon rank-sum test, corrected for multiple hypothesis testing using the Benjamini-Hochberg method. The results were visualized in heatmaps, where mean marker expression values were z-normalized across all expression values of interest.

##### Differential cell-type enrichment within CNp

Linear models *Y_n,c_* = β_0_ + β_1_*X* + β^3^*Y*_c_ + e were estimated, where *Y*_c_ is the log overall frequency of cell type c, *X* is an indicator variable for patient group, *Y_n,c_* is the log frequency of cell type c in CN n, β_i_ are coefficients, and e is mean zero Gaussian noise. A pseudo-count of 1e^−3^ was added prior to taking logs. These were estimated using the *statsmodels* Python package.^53^ The coefficient estimates and p-values for β_1_ were extracted and visualized using ComplexHeatmap.

##### Cell-cell interaction analysis

Briefly, Delaunay triangulation for each cell is determined based on x, y positions within each field of view using the default settings from the SciPy package. To identify interacting cells and their coordinates, relevant information is extracted automatically from the output. Subsequently, the distances between cells connected by the edges of a Delaunay triangle in the two-dimensional space are computed using the following formula: $Distance=\sqrt{{\left(x_{1}-x_{2}\right)}^{2}+{\left(y_{1}-y_{2}\right)}^{2}}$.

Cell-to-cell interactions within 265 pixels (=132.5μm) are identified. To establish a reference distribution of distances, 100 iterations of the triangulation calculation are conducted. In each iteration, the cells and their neighborhoods within each field of view are randomly reassigned to existing x, y positions. The average distances of cell-cell interactions for each field of view in each permutation are calculated and compared to the observed distances using a Mann-Whitney U test. The fold enrichments of distances between the observed data and the mean distances derived from the permutation test are determined. The log fold changes of distance for each pair of interactions with p-values less than 0.05 are plotted.

Furthermore, we employed QUICHE^17^ to first define local cellular niches as the proportion of cell types within a *k*-hop spatial neighborhood bounded by a fixed pixel radius, *s*, around each cell (here: 100px = 50 μm). To test for differential spatial organization of cell types across conditions, we used the approach previously described for QUICHE using graph neighborhoods. Specifically, the similarity of niches across tissue samples was modeled using a *k*-nearest neighbor graph, *𝒢* = (*𝒱*, *ε*), where the nodes *𝒱* represent niches and the edges are defined according to pairwise Euclidean distances of cell type frequency vectors. Hypothesis testing was done using a F-statistic with a weighted FDR multiple hypothesis testing correction procedure. This approach accounts for overlapping neighborhoods by adjusting *p*-values according to the connectivity of the graph. Therefore, the output of our approach is a set of spatially reoccurring cellular niches that are statistically associated with different LN tissues and patient outcomes, *g*.

##### Community and tissue unit identification analysis

Communities were determined similarly to how multicellular neighborhoods were determined with some small differences as described previously.^23^ In brief, the cells in the neighborhood tissue maps were taken with a larger window size of 100 nearest neighbors. These windows were taken across the entirety of the tissue and the vectors then clustered with *k*-means clustering and over-clustered with 10 total clusters. These clusters were mapped back to the tissue and evaluated for neighborhood composition and enrichment to determine overall community type.

##### Hierarchical LN structural graphs

Each hierarchical level was connected to the next by either what it contributed to the largest in the next level, or what made up at least 15% of this next hierarchy. The percentage of each feature in the level was represented by size of the shape. The shape and color combination correspond to the level and feature respectively. The size of the connecting line represents the amount that a feature contributes to the next feature.

##### Spatial context maps

Spatial context maps were created as previously described.^18^ First, a large window size (100 nearest neighbors) was used for each cell across the tissue neighborhood labels to create composition vectors. Second, instead of clustering the vectors of neighbor compositions, the combination that included the fewest neighborhoods making up more than 85% of the cells within that window was determined. This combination provides information about prominent associations of neighborhoods in the window, a feature termed spatial context. Third, each combination was counted, and the most prevalent combinations were connected to form a spatial context map. DESeq2 was used to calculate spatial contexts that were differentially enriched between metastatic lymph node samples from nodal-positive patients and their non-metastatic counterparts. Similarly, the DESeq2 package was used to calculate differentially enriched spatial contexts between metastatic and non-metastatic B16-F0 melanoma cell lines. Results were visualized as either bar plots or volcano plots using the EnhancedVolcano package.

##### Tissue graphs for HNSCC data

A graph for each region was built with nodes corresponding to instances of each CN and edges corresponding to pairs of instances that spatially intersected (i.e., had at least one pixel in common) in the binary images. Graphs were represented using the Python networkx library as previously described.^18^

##### Identification of higher-order 2-chains in nodal positive and nodal negative HNSCC patients

Motifs were considered higher-order if the assembly of CNs followed specific rules that significantly deviated from the maximum entropy null distribution after Bonferroni correction.

If CN-CN instances are less frequent than expected under the maximum entropy null distribution,^18^ this can be interpreted as evidence that active tissue processes prevent contact between these two CNs. By contrast, if two-chains are enriched within a group of patients this indicates that its assembly into the TME is actively specified, and thus adjacencies between its instances could play a role in anti-tumor immunity.

CN-CN assembly rules were visualized for both nodal-positive and nodal-negative patient groups among primary tumor samples. The Level-0 null set was defined as follows: The level-0 null set was equivalent to the set of CN assignments obtainable by arbitrary permutation of the assignments within each TMA core, as well as the set of CN assignments that maintained the number of vertices in each core assigned to each CN. The maximum entropy null distribution, given by the uniform distribution on this set, was sampled directly using random permutations from the numpy.random library as previously described.^18^

Only 2-chains with at least five instances were considered as candidate higher-order structures. One-sided hypothesis tests were conducted for these 2-chains against the hypothesis that the observed count arose from the count of that 2-chain as a sample assignment from the null distribution. The null hypothesis rejection p-value was estimated as the smaller of: (a) the proportion of permutations for which the observed count was greater than the count in the permuted assignment and (b) the proportion of permutations for which the observed count was less than the count in the permuted assignment. To identify significant 2-chains, p-values were Bonferroni corrected by multiplying by twice the number of tests conducted in each patient group.

##### Follicle extraction and patch-proximity analysis

Lymph node follicles from CODEX data of the HNSCC discovery cohort were manually annotated. Masks and x/y coordinates were extracted using a custom Python script and used for subsequent identification of cellular composition, marker expression, and cell-cell interactions within lymph node follicles. To analyze the composition of cells in spatial proximity to patches, LNFs were manually annotated, and those patches were detected in spatial single-cell data by running HDBSCAN clustering over all centroids. The resulting clusters were used as patches, and cells not part of a patch were ignored. The outermost cells of each patch were selected by constructing a concave hull and selecting the spanning points. To include cells that were close to the outlining border of the patch and to correct for distant cells, the three nearest neighbors for each edge cell were included, resulting in a group of cells surrounding each patch. In the subsequent step, a user-defined radius was drawn around each anchor cell to identify cells outside the patch within the defined radius. Here, the radius was set to 200 μm (400 px). In the final step, a custom script was used to compare the composition within the spatial proximity of follicles to the rest of the dataset to display the log2 fold enrichment. In this manuscript, cells and CNs within proximal distances of 10 to 50 μm from the border were examined. The method is described in detail in.^54^

#### Spatial transcriptomics

Tissue microarrays were sent to Akoya Biosciences for spatial transcriptomics data generation. The resulting imaging data was pre-processed (see below) and normalized prior to downstream computational analysis:

##### Processing

Spatial transcriptomics data were processed using a custom MATLAB script to identify and quantify cellular features from multiplexed imaging datasets. The workflow included the following steps: (1) dilation of nuclei masks to define cellular boundaries, (2) calculation of cellular centroids and areas using region-based segmentation, (3) extraction of intensity profiles from multiplexed imaging channels, and (4) detection and quantification of RNA dots within cells. The script allowed for interactive threshold selection for dot detection and integrated intensity computation. Outputs were organized into tables containing spatial and quantitative metrics for each cell, including x-y coordinates, cell area, RNA dot counts, and average intensity values for each channel. Results were saved in CSV format for downstream analysis.

##### Cell clustering

RNA spatial clustering analysis was conducted using a custom Python-based Jupyter Notebook. This workflow involved integrating RNA spot data from multiple regions by reading and concatenating CSV files containing spatial and expression data. Data preprocessing included removing low-quality cells, standardization and normalization of channels using z-score normalization. Dimensionality reduction and clustering were performed to identify specific cell types that were validated using gene expression vectors with heatmaps, scatter plots, and overlays to identify specific cell types.

##### Neighborhood analysis

Neighborhood-level spatial transcriptomics analysis was performed similar to the CODEX data (STAR Methods).

##### Cytokine neighborhood analysis

First, spatial coordinates of RNA spots were extracted and used to construct neighbor vectors using nearest-neighbor algorithms such as NearestNeighbors. Each vector contained the spatial indices of the k-nearest RNA spots, with 100 nearest RNA spots to capture immediate neighborhoods. These neighbors’ values were summed for all cytokines to the centroid spot. These new vectors were then clustered into specific cytokine neighborhoods validated with cytokine expression and spatial expression on tissues.

#### Construction of tissue microarray for the HNSCC validation and LN expansion cohorts

Similar to the initial discovery cohort we identified both for primary tumors and LN-metastasis each 2 regions from the tumor core and tumor edge for 1.5mm core diameter punch biopsies, whereas for adjacent non-tumor involved LN regions and for non-involved lymph nodes we only collected 1 representative 1.5mm region.

These 1.5mm core regions from the HNSCC validation cohort and healthy LN expansion cohort (see below) were assembled as a tissue microarray, that was sectioned at 3μm thickness onto SuperFrost PLUS slides and processed in the same way as for the initial discovery cohort for CODEX multiplex imaging.

From the LN expansion cohort, we identified each 2 representative regions of these LNs with a diameter of 1.5mm for TMA construction to accurately represent LN architecture of the LN microenvironment.

We stained this Validation TMA with a 56-marker panel including most of the markers used in the discovery cohort (for details see Table S2). Following antibody staining we prepared reporter plates and imaged the TMA on CODEX Phenocycler Fusion (Akoya Biosciences) as described previously.^55,56^

Cell segmentation and quality control were performed as described above where tissue cores with less than 3000 cells were excluded from CN analysis and further downstream analysis, thereby resulting in a dataset with 11 primary tumor regions, 10 LN-metastasis regions, 4 LN.adjacent regions, 5 LN.benign.N+ regions and 4 LN.benign.N− regions, while for the LN expansion cohort we included a final of 26 cervical LN-regions, 12 inguinal LN-regions and 13 mesenterial LN-regions into our dataset. Cell type annotations were conducted using leiden-based clustering for the major cell lineage markers as described above. Similar to our discovery cohort we identified CNs separately for primary tumor regions and LN tissues using k=15 nearest neighbors (see above).

##### *In-vivo* B16-F0 model

Cell suspensions of 2x10^5^ cells of from the previously described LN6-987AL cell line^5^ (referred to as met.CL), or the parental minimally metastatic B16-F0 melanoma cell line or PBS in phenol red free DMEM were injected into subcutaneous region of the left flank of eight-week old C57-BL/6J female mice (Jackson, 000664) following removal of fur with surgical clippers. In total 5 mice received the metastatic cell line, 7 mice were injected the minimally metastatic parental cell line, and two control mice were injected PBS only. Tumors were allowed to grow for 26 to 30 days or until mice were moribund, at which point mice were euthanized. Inguinal, brachial and axillary LNs were harvested. Tissues were isolated and fixed in 4% PFA, left in 30% w/v sucrose at 4° C overnight and embedded in optimal cutting temperature (OCT) compound (Tissue-Tek) the following day. Embedded tissues were frozen in isopentane cooled by liquid nitrogen.

#### Generation of tissue macroarrays and CODEX multiplex imaging of the murine arrays

For each of the 13 mice, 4-6 different lymph nodes were available, which were arranged in a tissue microarray and embedded in OCT. Macro-arrays were sectioned at 10 μm thickness onto 22x22 mm coverslips treated with Poly-L-Lysine and stored at −80°C until further processing.

CODEX staining was executed as previously described^51^: Briefly, coverslips were retrieved from the −80°C freezer, placed on DRIERITE for 2 minutes, followed by 10 minutes incubation in ethanol before being hydrated using low-salt S1 buffer and 1.6% PFA solution. Subsequently, sections were incubated for 20 minutes in blocking buffer, followed by 3-hour incubation with a mixture of validated antibody-oligo conjugates (detailed panel information can be found in Table S2). Following antibody incubation, sections were washed and finally fixed for 20 minutes using final-fixative BC3. The stained sections were stored in S4 at 4°C until imaging.

Following antibody staining, coverslips were handled as described in STAR Methods section: CODEX multi-cycle reaction and image acquisition. Similar to antibody-oligo conjugates optimized for FFPE tissue staining, murine antibody-conjugates were previously tested within positive control tissues of tumors or mouse spleens to validate the observed staining patterns. Each macro-array underwent CODEX multiplexed imaging; metadata from all CODEX runs can be found in Table S2.

##### Image processing and image analysis for the B16-F0 melanoma dataset

Stained coverslips were mounted onto custom acrylic plates with mounting gaskets, thereby creating a flow cell with a surface area of 12 × 15 mm above the tissue for fluid exchange. Acrylic plates were inserted into a Keyence BZ-X710 inverted fluorescence microscope equipped with a CFI Plan Apo λ 20×/0.75 objective (Nikon) using custom adapters. Coverslips corresponded to 5–6 LNs collected from a single mouse in the form of a macroarray. For each LN an imaging area sufficient to cover the complete cross-section was collected, except for LNs covering larger than 7x9 fields of view. Multicycle imaging was performed using a CODEX microfluidics device and CODEX driver software v1.29.0.1 (Akoya Biosciences).

For each imaging field analyzed by CODEX multidimensional staining multi-color z stacks collected during individual cycles were aligned against a reference channel (Hoechst 3342) by 3D drift compensation.^57^ Fields covering large tiled areas were “stitched” using dedicated ImageJ plugin.^58^ For the 49-channel CODEX experiment, images corresponding to the best focal plane of vertical image stacks collected at each acquisition step of CODEX were chosen for quantification. Segmentation was performed on the whole image stack using a volumetric segmentation algorithm as described previously.^59^

##### Spatial spillover compensation

Accurate segmentation alone is not sufficient to obtain high-quality estimates of single-cell expression from images. This is because, in lymphoid tissue, cells are tightly adjacent, leading to membrane signals partially overlapping and resulting in signal blending between neighboring cells, a phenomenon termed spatial spillover. A previously described method was used to reduce spatial signal spillover and preprocess the data for the removal of debris, autofluorescent objects, and doublets.^59^

The resulting segmented B16-F0 dataset underwent segmentation, quantification, compensation, and cleanup gating, yielding a total of 3,425,185 cells, each with 49 single-cell protein marker expression profiles. The CODEX data was used for automated phenotype mapping using an X-shift algorithm that was previously developed and validated on CyTOF data.^60^ Phenotypic clusters inferred by X-shift clustering were manually annotated based on the 49-color marker expression profile and thorough visual inspection of the representative image samples. Each cluster was assigned to one of 48 broadly defined single-cell phenotypic groups (cell types), which in some cases could be clearly matched to major immune cell types, while in others, they were named according to the expression of distinguishing surface markers. To allow for comparisons of the human and murine datasets, we manually annotated tumor-involved LN-regions and adjacent tumor-free LN regions. We exported these masks to label cell types within these microscopically distinct regions. This allowed us to separate tumor-involved LN regions (LN.met), adjacent tumor-free regions (LN.adjacent), tumor-free lymph nodes from mice that developed distant metastasis (that were exclusively from the metastatic LN6 cell line; LN.benign.N+) and lymph nodes from mice that did not develop LN-metastasis that were derived from the non-metastatic B16F0 melanoma cell line and wildtype non-tumor control animals (LN.benign.N−).

#### Clinical data analysis

Descriptive statistics were used to analyze the baseline characteristics of the study population. Chi-square test was used to assess the association between the compared groups. The Clopper–Pearson method was used to calculate 95% confidence intervals for the categorical variables. As time-to-event endpoints, this retrospective cohort study used DMFS, which was estimated using the Kaplan–Meier product-limit method and log-rank statistics using the *survminer* package in R. Independent prognostic values of clinical patients’ characteristics were estimated using univariate and multivariable Cox proportional hazard models. Multivariable analysis was calculated for the significant variables by the univariate test or *a priori* selection for biological relevance to evaluate their conjoint, independent effects on DMFS. In all cases, two-tailed p-values were calculated and considered significant with *p* < 0.05.

### QUANTIFICATION AND STATISTICAL ANALYSIS

Statistical analyses were performed with RStudio (version 2023.06.1) using packages *ggpubr* and *stats* if not specified otherwise and Python (version 3.8.11) packages *scipy* or *statsmodels* if not specified otherwise. Results with p < 0.05 were considered significant unless otherwise stated. No statistical methods were used to predetermine sample size. For differences across the tested groups, including comparisons of nodal-positive and nodal-negative HNSCC patients, the significance was tested using a two-tailed Wilcoxon-rank sum test corrected for multiple hypothesis testing (Benjamini-Hochberg; short: BH). Tests involving comparisons between more than two groups were conducted using the Kruskal-Wallis test. If boxplots were used to illustrate group differences, boxplots characteristics are defined as follows: center line of boxplots depicts median and box-limits the 25 and 75 percentiles of each condition. Whiskers are defined as 1.5x interquartile range with jitter dots depicting data from each individual region. Tests involving comparison of groups with categorical values were performed using two-sided Fisher’s exact tests. All statistical tests were two-sided unless otherwise specified. Correlations were reported by the Spearman rank correlation and adjusted using the Benjamini-Hochberg correction. Hazard ratios and results from log-rank tests for survival analysis were calculated using the survival package in R. For differential gene expression between patient groups in spatial transcriptomics data, we employed the R-package DESeq2. The following abbreviations apply if not specified otherwise: *p < 0.05, **p < 0.01, ***p < 0.001, ****p < 0.0001.

## Supplementary Material

Document S1

Table S4

Table S5

Table S3

Table S2

Supplemental information can be found online at https://doi.org/10.1016/j.ccell.2026.01.003.

## Figures and Tables

**Table 1.** Patient characteristics of the HNSCC discovery cohort

Clinicopathological features	Number of patients (%)	Nodal negative patients	Nodal positive patients	*p*-value
Total number of patients	78	29	49	–
Baseline patient and tumor characteristics				
Median age at diagnosis (range)	57.0 years (15–86)	55.0 years (33–79)	57.0 years (15–86)	0.839
Sex				0.359
Female	35 (44.9%)	11 (37.9%)	24 (49.0%)	
Male	43 (55.1%)	18 (62.1%)	25 (51.0%)	
Primary tumor location				0.701
Oral tongue	55 (70.5%)	19 (65.5%)	36 (73.5%)	
Retromolar trigonum	4 (5.1%)	1 (3.4%)	3 (6.1%)	
Buccal mucosa	4 (5.1%)	1 (3.4%)	3 (6.1%)	
Alveolar ridge	3 (3.8%)	2 (6.9%)	1 (2%)	
Lip	1 (1.3%)	0	1 (2%)	
Hard palate	2 (2.6%)	1 (3.4%)	1 (2%)	
Floor of mouth	9 (11.5%)	5 (17.2%)	4 (8.2%)	
p16-status^a^				–
Negative	60	21	39	
Positive	17	7	10	
HPV-status of p16-positive^a^				–
Positive	0			
Negative	15/17 (100%)	5	10	
T-stage				0.350
T1	6 (7.7%)	4 (13.8%)	2 (4.1%)	
T2	37 (47.4%)	11 (37.9%)	26 (53.1%)	
T3	19 (24.4%)	7 (24.1%)	12 (24.5%)	
T4a	16 (20.5%)	7 (24.1%)	9 (18.4%)	
N-stage				–
N0	29 (37.2%)	29	0	
N1	15 (19.2%)	0	15	
N2a	1 (1.3%)	0	1	
N2b	23 (29.5%)	0	23	
N2c	9 (11.5%)	0	9	
N3	1 (1.3%)	0	1	
AJCC 7 staging system				<**0.001**
I	4 (5.1%)	4 (13.8%)	0	
II	11 (14.1%)	11 (37.9%)	0	
III	21 (26.9%)	7 (24.1%)	14 (28.6%)	
IVA	41 (52.6%)	7 (24.1%)	34 (69.4%)	
IVB	1 (1.3%)	0	1 (2.0%)	
IVC	0	0	0	
Median Tumor size (mm)	32.5 (7–85)	32.5 (7–54)	33 (7–85)	0.175
Median tumor depth (mm)	13.5 (3–39)	11 (3–30)	14 (4–39)	0.056
Perineural invasion	55/78 (70.5%)	21 (72.4%)	34 (69.4%)	1.0
Former smoker	26/78 (33.8%)	11 (37.9%)	15 (31.3%)	0.662
Current smoker	12/78 (15.4%)	3 (10.3%)	9 (18.4%)	0.518
Treatments and outcomes				
Surgical removal	78/78 (100%)	29	49	–
Neck dissection	57/78 (73.1%)	11 (37.9%)	46 (93.9%)	<**0.001**
Adjuvant RTx^b^	71/78 (88.8%)	25	46	–
Concurrent adjuvant CTx and RTx	33/54^c^ (61.1%)	7	26	**0.010**
Recurrence^d^	34/72 (47.2%)	10 (38.5%)	24 (52.2%)	0.329
Local	19/71 (26.8%)	8 (38.4%)	11 (45.8%)	0.128
Locoregional	21/71 (29.6%)	6 (54.5%)	15 (62.5%)	0.709
Distant	21/71 (29.6%)	3 (11.5%)	18 (40.0%)	**0.015**
Median relapse-free survival (95% CI)	88.0 months (NR)	NR (51.0-NR)	15.0 months (12.0–68.5)	0.139
5-year RFS	52.7% (42.0–66.2%)	62.3% (45.6–85.3%)	47.6% (34.8–65.1%)	–
Median distant metastasis free survival (95% CI)	NR	NR	NR	**0.042**
5-year DMFS	63.0% (51.3–77.3%)	82.6% (66.6–100%)	53.6% (39.6–72.6%)	–
Survival and Follow-up				
Median follow-up upon initial diagnosis (95% CI)	75 months (55.7–94.3 months)	80.0 months (59.0–117.0)	73.0 months (42.0–95.0)	0.176
Median overall survival (95% CI)	222.0 months (89.0-NR)	222.0 months (99-NR)	107.0 months (38-NR)	0.08
5-year OS (95% CI)	72.8% (62.5–84.9%)	87.6% (75.4–100%)	63.4% (48.3–80.5%)	–
Deceased^e^	24/75 (32.0%)	7 (25.9%)	17 (35.4%)	0.398
HNSCC-specific median overall survival	–	66.0 months (7-NR)	19.0 months (13-NR)	0.26
HNSCC-specific death^f^	14/17 (82.4%)	2 (50.0%)	12 (92.3%)	0.052

Cohort description including summary of baseline patient demographics, tumor characteristics, cancer-directed treatments and survival outcomes. Categories are printed in bold. Abbreviations: HNSCC, head-and-neck squamous cell carcinoma; OS, overall survival; RFS, relapse-free survival; DMFS, distant-metastasis-free survival; CTx, chemotherapy; RTx, radiotherapy; CI, confidence interval; NR, not reached.

a Data on p16-status was available in 77/78 patients, while data on HPV-status (as assessed via PCR or ISH) was available in 26/78 patients. Thereof, 15/17 patients were classified as p16-positive cases.

b Information on curative RTx was available in 71 patients (25 nodal negative and 46 nodal positive patients).

c Information on concurrent CTx was available for 54 patients

d Information on tumor recurrence was available for 72 patients (26 nodal negative and 46 nodal positive patients).

e Data on survival status were available for 75 patients.

f Data on HNSCC-specific death were available for 17 out of 24 patients that deceased.

**Table:** KEY RESOURCES TABLE

REAGENT or RESOURCE	SOURCE	IDENTIFIER
Antibodies		
Purified anti-human and anti-mouse antibodies, see Tables S2A–S2C	various	various
Mouse IgG	Sigma	I5381
Rat IgG	Sigma	I4131
Biological samples		
Head-and-neck squamous cell carcinoma tissue blocks	Department of Pathology, Stanford Unversity Medical School	N/A
Head-and-neck squamous cell carcinoma tissue blocks	Department of Pathology, University Medical Center of the Johannes Gutenberg University Mainz	N/A
Benign Lymph nodes from non-cancer patients	Department of Pathology, University Medical Center of the Johannes Gutenberg University Mainz	N/A
Chemicals, peptides, and recombinant proteins		
Xylene	Thermo Fisher Scientific	D159-4
Ethanol (Proof 200)	N/A	N/A
PBS	Thermo Fisher Scientific	14190-250
NaCl	Thermo Fisher Scientific	S271-10
Na_2_HPO_4_	Sigma	S7907
NaH_2_PO_4_	Sigma	S9390
MgCl_2_	Sigma	M2670
NaN_3_	Sigma	S8032
EDTA	Sigma	93302
TCEP	Sigma	C4706
NaOH	Sigma	S8263
BS3	Thermo Fisher Scientific	21580
DMSO	Thermo Fisher Scientific	D128-4
DMSO ampoules	Sigma	D2650
Paraformaldehyde ampoules, 16%	Thermo Fisher Scientific	50-980-487
BSA	Sigma	A3059
Tris 1 M, pH 8.0	Teknova	T1080
Candor PBS antibody stabilizer solution	Candor Biosciences	131–050
Salmon sperm DNA, sheared	Thermo Fisher Scientific	AM9680
Triton^™^ X-100	Sigma	T8787
Ethanol, 100%	Sigma	E7023
Acetone, 100%	Thermo Fisher Scientific	A929-4
Methanol, 100%	Thermo Fisher Scientific	A412-4
Trizma^®^ HCl	Sigma	T3253
Trizma^®^ Base	Sigma	T1503
Bondic polyacrylamide gel	Amazon	B018IBEHQU
Dako target retrieval solution, pH 9.0	DAKO	S2367
TBS IHC wash buffer with Tween^®^ 20	Cell Marque	935B-09
Antibody diluent	DAKO	S0809
Protein block, serum-free	Agilent	X090930-2
Normal Horse Serum, 2.5%	Vector Laboratories	S-2012
Hematoxylin, ready-to-use	Agilent	CS700
Eosin Y solution	Sigma	HT110116
Cytoseal XYL	Thermo Fisher Scientific	8312–4
Hoechst 33342	Thermo Fisher Scientific	62249
DRAQ5	Cell Signaling Technology	4084L
Critical commercial assays		
LTS filter tips, 10 μL	N/A	N/A
LTS filter tips, 200 μL	Rainin	30389239
LTS filter tips, 1000 μL	Rainin	30389212
Amicon^™^ Ultra Centrifugal Filters, 50kDa	Thermo Fisher Scientific	UFC505096
Nalgene^™^ Rapid Flow 500 mL filter, 0.2 μm	Thermo Fisher Scientific	09-740-28C
Glass coverslips, 22 × 22 mm, # 1 1/2	Electron Microscopy Sciences	72204–01
Frosted microscope slides	Thermo Fisher Scientific	12-550-343
Glass coverslip storage box	Qintay	CS-22
22 × 22 mm coverslip mounting gaskets	Qintay	TMG-22
25 × 25 mm coverslip mounting gaskets	Qintay	TMG-25
Wheaton^™^ Coverslip glass jars	Thermo Fisher Scientific	02-912-637
Dumont #5/45 coverslip forceps	Fine Science Tools	11251–33
ST4020 small linear stainer	Leica	14050946425
Lab Vision^™^ PT module	Thermo Fisher Scientific	A80400012
8-strip tubes, 0.2 mL	E&K Scientific	280008
8-strip caps, flat top	E&K Scientific	491008
8-strip caps, dome top	E&K Scientific	491018
Vectabond^™^	Vector Labs	SP-1800
Corning^™^ black 96-well plates	Thermo Fisher Scientific	07-200-762
Axygen aluminum sealing film	VWR Scientific	47734–817
Deposited data		
Single-cell data table for human HNSCC discovery cohort (CODEX)	Mendeley data	https://doi.org/10.17632/t4k5kj3cxr.1
Single-cell data table for human HNSCC validation cohort and benign LN atlas (CODEX)	Mendeley data	https://doi.org/10.17632/t4k5kj3cxr.1
Primary imaging data for the human HNSCC discovery and validation cohort (CODEX and corresponding H&E)	BioImage Archive	https://doi.org/10.6019/S-BIAD1638
Primary imaging data for the human benign non-cancer LN atlas (CODEX and corresponding H&E)	BioImage Archive	https://doi.org/10.6019/S-BIAD1638
Single-cell data table for murine B16-F0 melanoma model (CODEX)	Mendeley data	https://doi.org/10.17632/t4k5kj3cxr.1
Primary imaging data for murine B16-F0 melanoma model (CODEX)	BioImage Archive	https://doi.org/10.6019/S-BIAD1638
Processed spatial transcriptomics data	Mendeley data	https://doi.org/10.17632/t4k5kj3cxr.1
Experimental models: Cell lines		
B16-F0	ATCC	CRL-6322
LN Lines	N/A	Reticker-Flynn et al.^5^
Experimental models: Organisms/strains		
C57BL/6J	Jackson Laboratories	#000664
Oligonucleotides		
CODEX oligonucleotides, as previously described^13^	N/A	N/A
Software and algorithms		
BZ-X viewer	Keyence	N/A
RAPID	https://github.com/nolanlab/RAPID	Lu et al.^14^
CODEX Toolkit, version 1.3.5	https://github.com/nolanlab/CODEX	Black et al.^51^
ImageJ (Fiji version 2.0.0)	https://imagej.net/	N/A
R, version 4.3.1.	https://www.r-project.org	N/A
R studio desktop, version 2023.06.1	https://www.rstudio.com/	N/A
Neighborhood analysis notebooks	https://github.com/nolanlab/NeighborhoodCoordination	Schurch et al.^13^
Statsmodel Python package	https://www.statsmodels.org/	Lee et al.^52^
Scikit learn Python package	https://scikit-learn.org/	Seabold and Perktold^53^
Spatial context maps	https://zenodo.org/records/5494009	Bhate et al.^18^
Other		
BZ-X710 fluorescence microscope	Keyence	N/A
CODEX acrylic plates	Bayview Plastic Solutions	custom made
Vacuum Desiccators, 23L	Thermo Fisher Scientific	08-648-112
Aperio AT2 DX System brightfield scanner	DMI Medical USA	N/A
Phenocycler Fusion	Akoya Biosciences	N/A
CODEX System	Akoya Biosciences	N/A

## Data Availability

No original code was generated for this study. Single cell data tables for the multiplex imaging of the HNSCC and normal LN data, the murine B16-F0 cohort, and ST data are deposited at Mendeley Data (https://doi.org/10.17632/t4k5kj3cxr.1). All pre-processed CODEX imaging data is available at The BioImage Archive (https://doi.org/10.6019/S-BIAD1638). Anonymized clinical metadata are reported in Tables S3, S4, and S5. Any additional information required to reanalyze the data reported in this article is available from the lead contact upon request.

## Highlights

- Spatial multi-omics defines cellular features of LN-mediated metastatic tolerance
- LN-colonization is linked to spatially organized myeloid-CAF interactions
- Intersections of myeloid/CAF niches and LN-follicles are associated with T cell dysfunction
- Spatial signatures extend to adjacent tumor-free LNs, absent in non-cancer patients
