# Supplementary material for: Lymph node colonization induces tissue remodeling via immunosuppressive fibroblast-myeloid cell niches supporting metastatic tolerance
Source: Cancer Cell. Author manuscript; Available in PMC 2026 Jun 23. (PMC13289805; doi:10.1016/j.ccell.2026.01.003)
Supplement: Document S1 [file NIHMS2186495-supplement-Document_S1.pdf]

Cancer Cell, Volume *44*

Supplemental information

Lymph node colonization induces tissue remodeling via immunosuppressive fibroblast-myeloid cell niches supporting metastatic tolerance

Maximilian Haist, Marc-A. Baertsch, Nathan E. Reticker-Flynn, Guolan Lu, Tim N. Kempchen, Pauline Chu, Gustavo Vazquez, Han Chen, John B. Sunwoo, Weiruo Zhang, Eyiwunmi Laseinde, Bonny Adami, Stefanie Zimmer, Justus Kaufman, Quynh Thu Le, Andrew J. Gentles, Christina S. Kong, Sylvia K. Plevritis, Yury Goltsev, John W. Hickey, and Garry P. Nolan

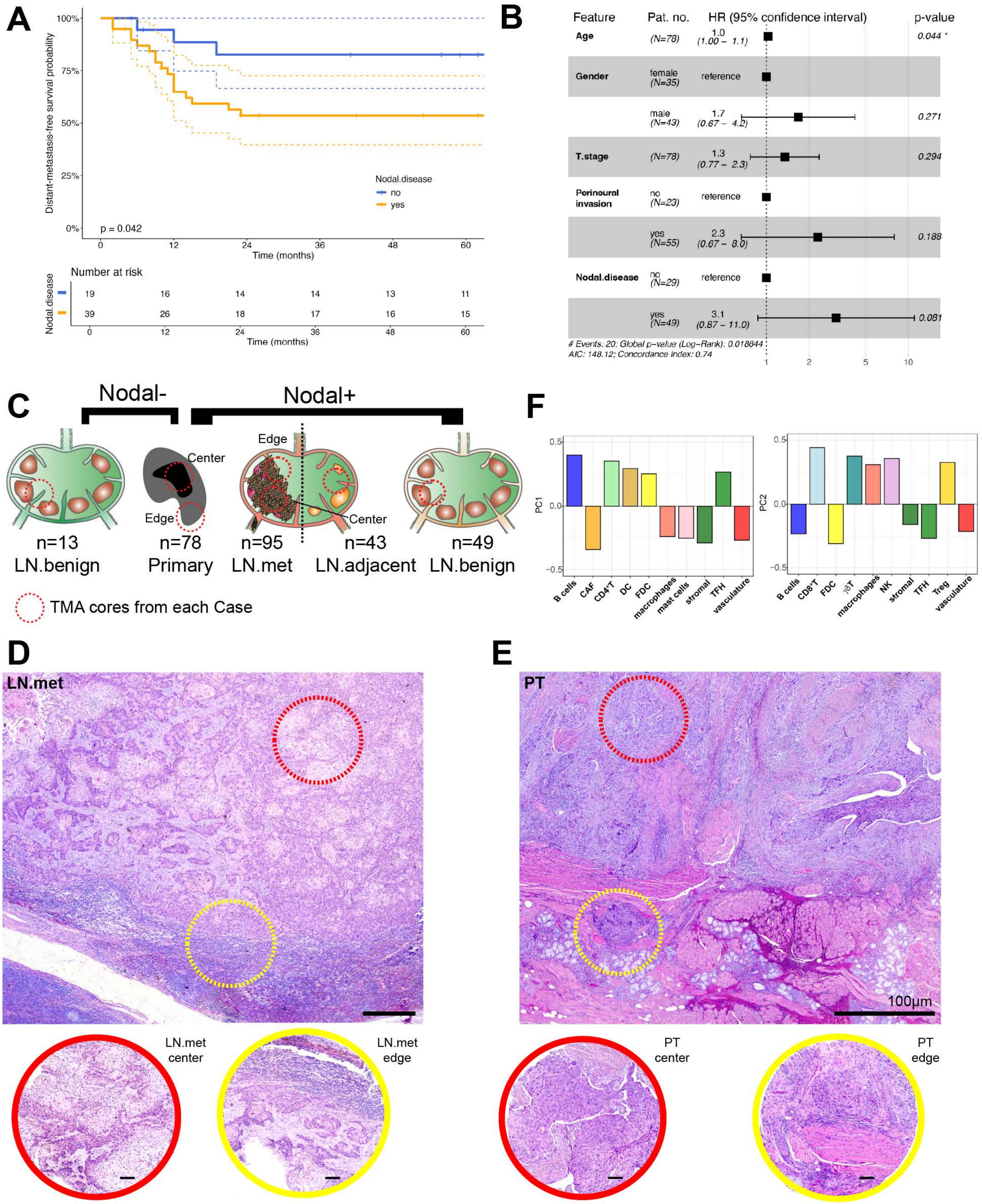
A
Distant-metastasis-free survival probability
100%
75%
50%
25%
0%
p = 0.042
Nodal.disease
no
yes
Time (months)
Number at risk
Nodal.disease
19 16 14 14 13 11
39 26 18 17 16 15
B
Feature
Pat. no.
HR (95% confidence interval)
p-value
Age (N=78) 1.0 (1.00 – 1.1) 0.044 *
Gender female (N=35) reference
male (N=43) 1.7 (0.67 – 4.2) 0.271
T.stage (N=78) 1.3 (0.77 – 2.3) 0.294
Perineural invasion no (N=23) reference
yes (N=55) 2.3 (0.67 – 8.0) 0.188
Nodal.disease no (N=29) reference
yes (N=49) 3.1 (0.87 – 11.0) 0.081
Events: 20; Global p-value (Log-Rank): 0.018644
AIC: 148.12; Concordance Index: 0.74
C
Nodal-
Nodal+
Center
Edge
Edge
Center
n=13 LN.benign
n=78 Primary
n=95 LN.met
n=43 LN.adjacent
n=49 LN.benign
TMA cores from each Case
F
PC1
PC2
B cells
CAF
CD4T
DC
FDC
macrophages
mast cells
stromal
TFH
vasculature
CD8T
γδT
NK
Treg
D
LN.met
E
PT
100µm
LN.met center
LN.met edge
PT center
PT edge

Figure S1: Clinical and molecular characteristics of the HNSCC discovery cohort and ROI selection for construction of the CODEX multiplex imaging TMA. Related to Figure 1.

(A) Kaplan Meier plot depicting distant-metastasis free survival for all HNSCC patients with available information on distant metastasis. Statistical significance was determined using log-rank test.

(B) Forest plot for distant-metastasis-free survival showing results from multivariate cox-regression analysis.

(C) Schematic summarizing tissue regions that have been assembled on the investigated TMA for the HNSCC discovery cohort from nodal-negative and nodal-positive cases (n=3 cases were excluded due to unknown nodal status).

(D) Representative example for the selection of tissue regions from a lymph node metastasis (top) with magnified regions from the invasive edge (bottom right) and tumor center (bottom left).

(E) Representative example for the selection of tissue regions from a primary tumor (top) with magnified regions from the invasive edge (bottom right) and tumor center (bottom left). Scalebar of 100µm applies to (D) and (E).

(F) Barplot summarizing contribution of single cell types to the principal components (PC) shown in Figure 1H

Abbreviations: CAF = cancer-associated fibroblasts; DC = dendritic cells; FDC = follicular dendritic cells; TFH = T-follicular helper cells; PT = primary tumor; LN.met = LN metastasis; LN.adj = adjacent non-tumor infested region of a metastatic LN; LN.benign = non-tumor infested LN; Treg = regulatory T cells

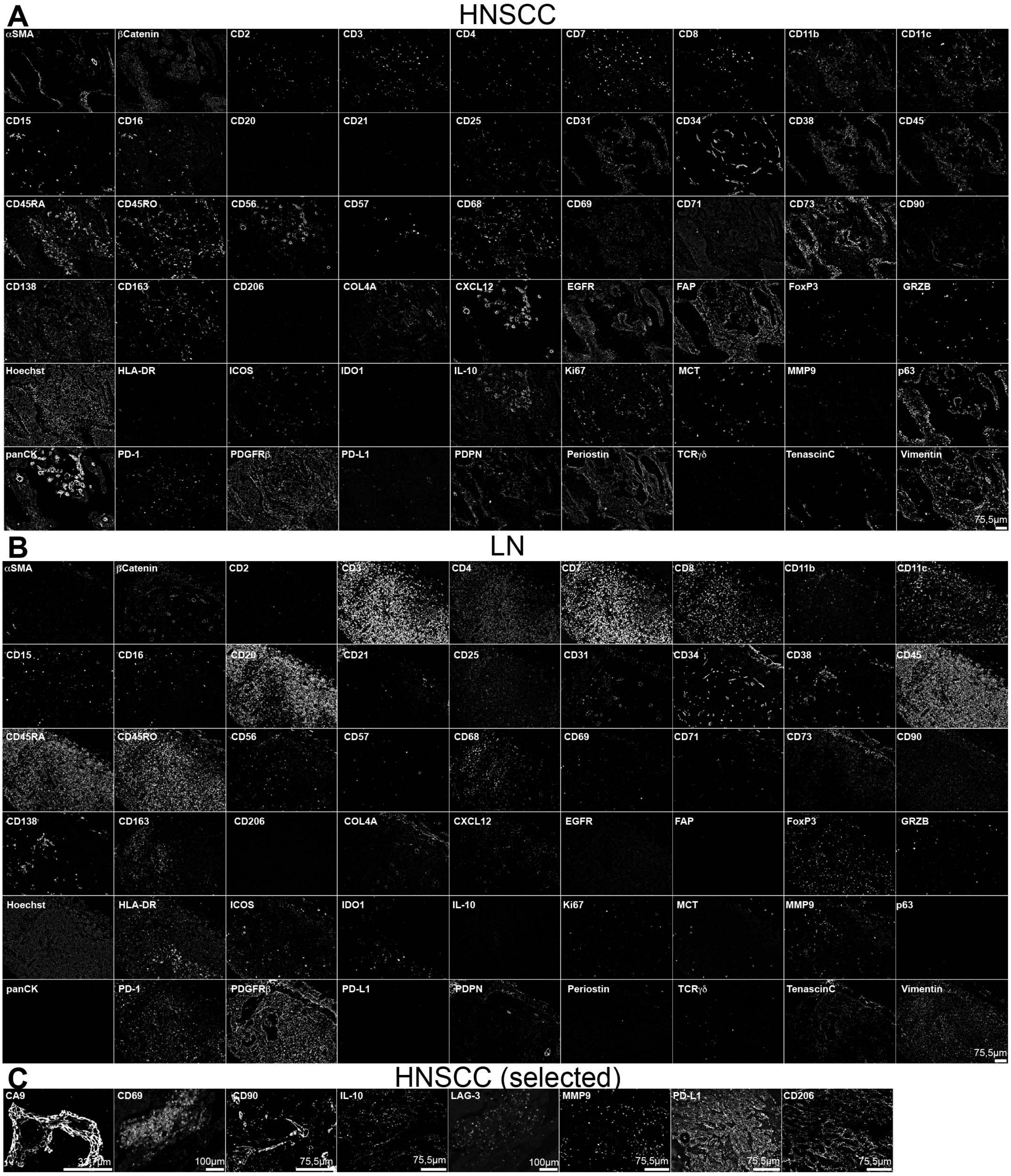
A
HNSCC
αSMA
βCatenin
CD2
CD3
CD4
CD7
CD8
CD11b
CD11c
CD15
CD16
CD20
CD21
CD25
CD31
CD34
CD38
CD45
CD45RA
CD45RO
CD56
CD57
CD68
CD69
CD71
CD73
CD90
CD138
CD163
CD206
COL4A
CXCL12
EGFR
FAP
FoxP3
GRZB
Hoechst
HLA-DR
ICOS
IDO1
IL-10
Ki67
MCT
MMP9
p63
panCK
PD-1
PDGFRβ
PD-L1
PDPN
Periostin
TCRγδ
TenascinC
Vimentin
75,5µm
B
LN
αSMA
βCatenin
CD2
CD3
CD4
CD7
CD8
CD11b
CD11c
CD15
CD16
CD20
CD21
CD25
CD31
CD34
CD38
CD45
CD45RA
CD45RO
CD56
CD57
CD68
CD69
CD71
CD73
CD90
CD138
CD163
CD206
COL4A
CXCL12
EGFR
FAP
FoxP3
GRZB
Hoechst
HLA-DR
ICOS
IDO1
IL-10
Ki67
MCT
MMP9
p63
panCK
PD-1
PDGFRβ
PD-L1
PDPN
Periostin
TCRγδ
TenascinC
Vimentin
75,5µm
C
HNSCC (selected)
CA9
CD69
100µm
CD90
75,5µm
IL-10
75,5µm
LAG-3
100µm
MMP9
75,5µm
PD-L1
75,5µm
CD206
75,5µm

Figure S2: Validation of the CODEX multiplex imaging antibody panel. Related to Figure 1.

(A) Single-plex greyscale images of each marker in the panel in a primary HNSCC tumor sample. Scale bar of 75.5µm applies to all panels.

(B) Single-plex greyscale images of each marker in the panel in a non-tumor infiltrated lymph node. Scale bar of 75.5µm applies to all panels.

(C) Single-plex greyscale images of selected markers not detected in the tissue cores shown in A) or B).

Abbreviations: CA9 = carbonic anhydrase 9; COL4A = Collagen IVA; GRZB = Granzyme B; PDPN = Podoplanin

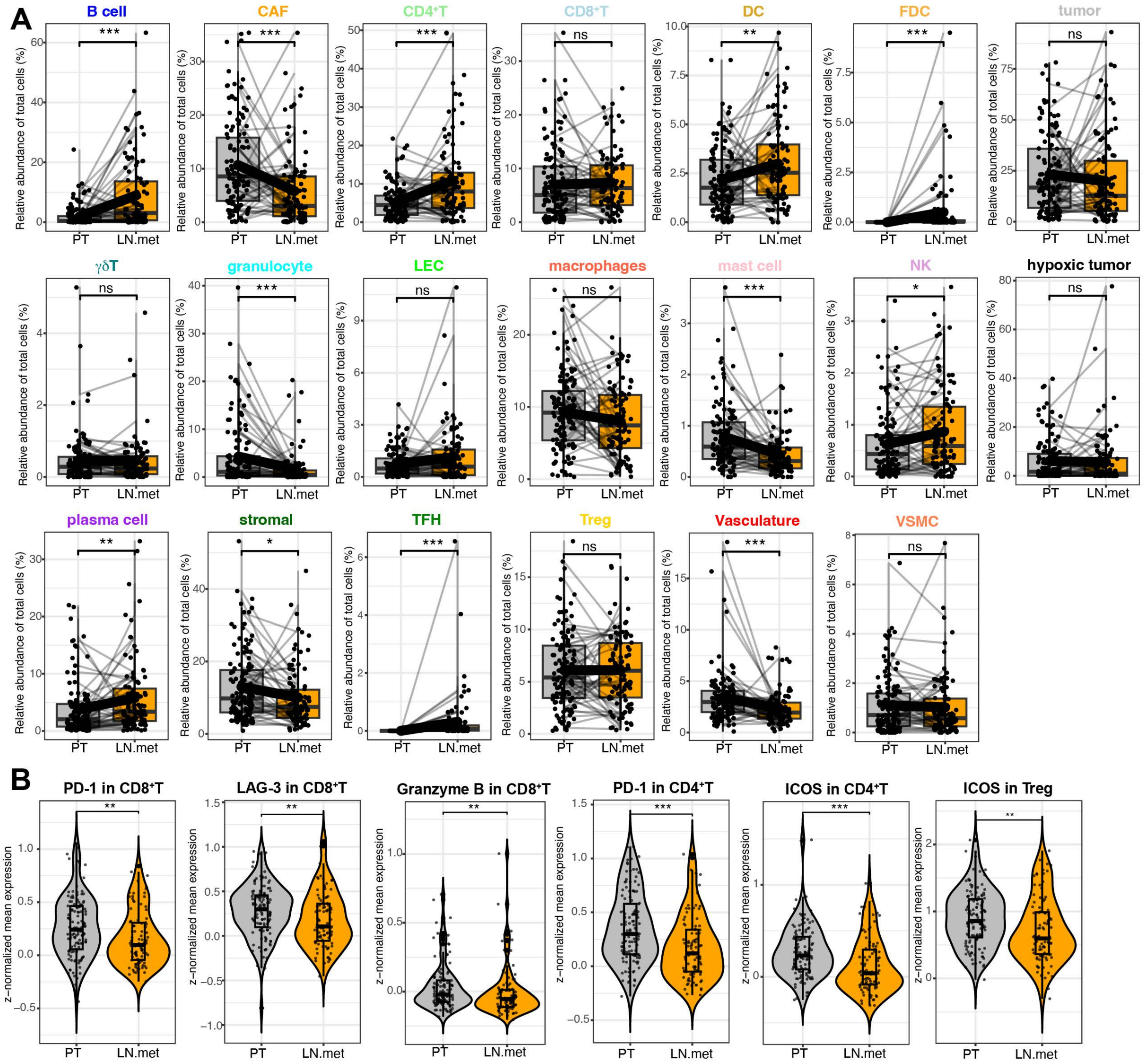
A
B cell
CAF
CD4⁺T
CD8⁺T
DC
FDC
tumor
γδT
granulocyte
LEC
macrophages
mast cell
NK
hypoxic tumor
plasma cell
stromal
TFH
Treg
Vasculature
VSMC
Relative abundance of total cells (%)
PT
LN.met
***
**
*
ns
B
PD-1 in CD8⁺T
LAG-3 in CD8⁺T
Granzyme B in CD8⁺T
PD-1 in CD4⁺T
ICOS in CD4⁺T
ICOS in Treg
z−normalized mean expression

Figure S3: Cellular, phenotypical and spatial organization of primary HNSCC samples and their paired metastatic lymph nodes. Related to Figure 2.

(A) Boxplots summarizing the mean cellular abundances of main cell-types between paired primary tumors and metastatic LN-regions. Thin edges connecting paired primary (n=125) and LN.met samples (n=95), whereas thick edges highlight the overall trend in cellular abundance between primary tumors and metastatic lymph nodes. Statistical significance was determined using two-sided Wilcoxon-rank sum test, adjusted for multiple hypothesis testing (BH). Center line of boxplots depicts median and box-limits the 25 and 75 percentiles of each condition. Whiskers are defined as 1.5x interquartile range with jitter dots depicting data from each individual region.

(B) Combined violin-boxplots depicting the mean expression of selected markers in major cell types between paired primary tumor and metastatic lymph node samples. Statistical significance was determined using two-sided Wilcoxon-rank sum test, adjusted for multiple hypothesis testing (BH). Center line of boxplots depicts median and box-limits the 25 and 75 percentiles of each condition. Whiskers are defined as 1.5x interquartile range with jitter dots depicting data from each individual region.

Asterixis indicate significant results from two-sided Wilcoxon test adjusted for multiple testing, ns = not significant, *p<0.05, **p<0.01, ***p<0.001.

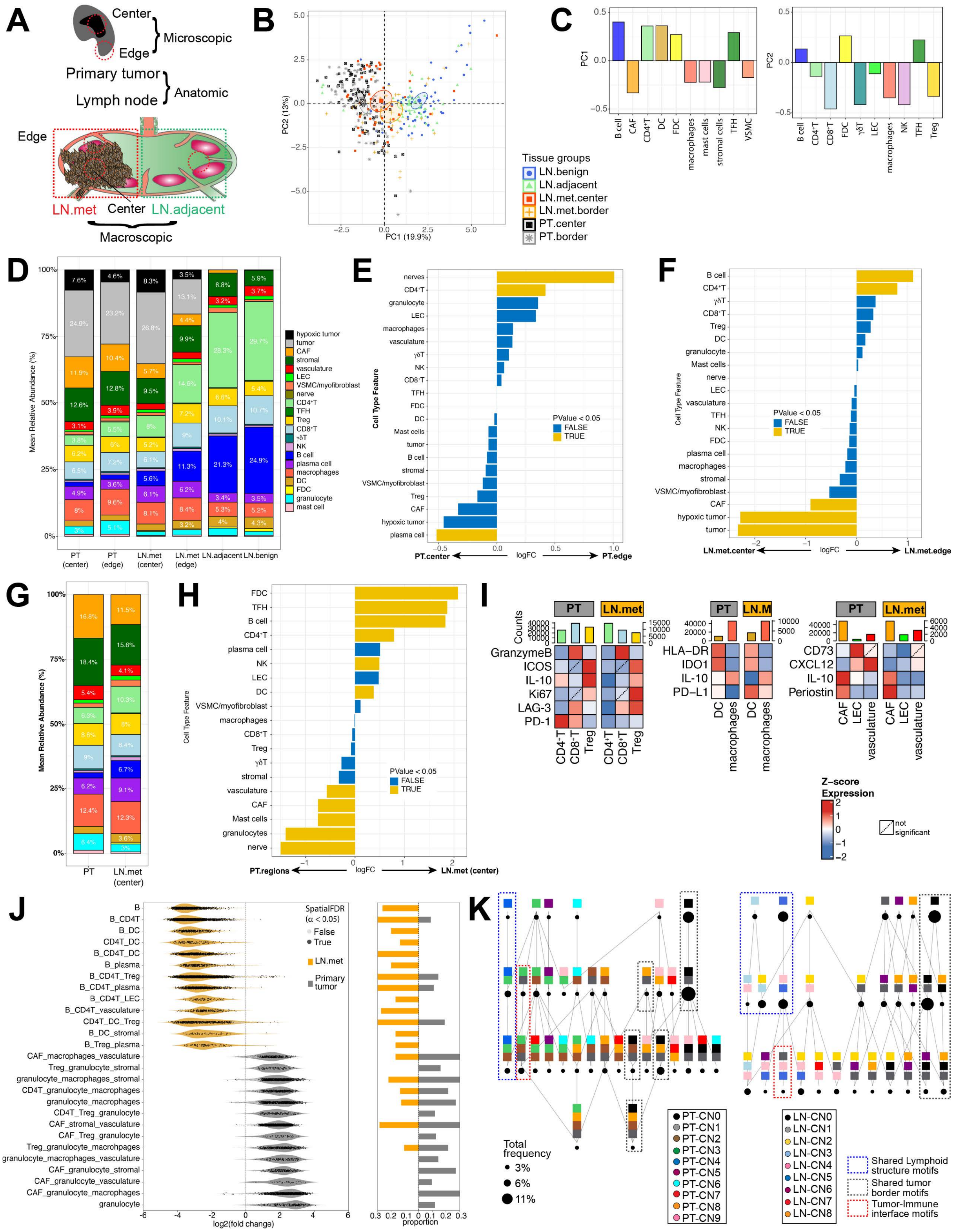
A
Center
Edge
Microscopic
Primary tumor
Lymph node
Anatomic
Edge
LN.met
Center
LN.adjacent
Macroscopic
B
PC1 (19.9%)
PC2 (13%)
Tissue groups
LN.benign
LN.adjacent
LN.met.center
LN.met.border
PT.center
PT.border
C
PC1
PC2
B cell
CAF
CD4⁺T
DC
FDC
macrophages
mast cells
stromal cells
TFH
VSMC
CD8⁺T
γδT
LEC
NK
Treg
D
Mean Relative Abundance (%)
PT (center)
PT (edge)
LN.met (center)
LN.met (edge)
LN.adjacent
LN.benign
hypoxic tumor
tumor
CAF
stromal
vasculature
LEC
VSMC/myofibroblast
nerve
CD4⁺T
TFH
Treg
CD8⁺T
γδT
NK
B cell
plasma cell
macrophages
DC
FDC
granulocyte
mast cell
E
Cell Type Feature
logFC
PT.center
PT.edge
PValue < 0.05
FALSE
TRUE
F
LN.met.center
LN.met.edge
G
Mean Relative Abundance (%)
PT
LN.met (center)
H
PT.regions
LN.met (center)
I
Counts
GranzymeB
ICOS
IL-10
Ki67
LAG-3
PD-1
HLA–DR
IDO1
PD–L1
CD73
CXCL12
Periostin
Z-score Expression
not significant
J
SpatialFDR (α < 0.05)
False
True
LN.met
Primary tumor
log2(fold change)
proportion
K
Total frequency
3%
6%
11%
PT-CN0
PT-CN1
PT-CN2
PT-CN3
PT-CN4
PT-CN5
PT-CN6
PT-CN7
PT-CN8
PT-CN9
LN-CN0
LN-CN1
LN-CN2
LN-CN3
LN-CN4
LN-CN5
LN-CN6
LN-CN7
LN-CN8
Shared Lymphoid structure motifs
Shared Tumor border motifs
Tumor-Immune interface motifs

Figure S4: Compositional and phenotypical characteristics within the primary TME and metastatic lymph node microenvironment stratified by selected microscopic sub-regions. Related to Figure 2.

(A) Schematic depicting the distinct microscopic regions that were taken from the analyzed tissues to investigate both the tumor center and edge regions to capture tissue heterogeneity.

(B) Results from principal-component analysis using major cell type abundances within all investigated tissue regions (n=390) of the CODEX dataset excluding the tumor compartment. Individual samples are colored according to macroscopic region within a given tissue type (PT.center, n=80; PT.edge, n=77; LN.met.center, n=40; LN.met.edge, n=40; LN.adjacent, n=43; LN.benign, n=63) excluding such without specification of the macroscopic region (PT.unspecified, n=32; LN.met.unspecified, n=15). Ellipses represent 95% confidence interval for each macroscopically distinct regin of a given tissue type based on multivariate normal distribution assumption.

(C) Barplot showing individual contributions of single cell types to the principal components (PC) shown in Figure S4B.

(D) Stacked barplots summarizing the mean abundance of all identified cell-types within each investigated tissue region stratified by the investigated microscopic sub-regions.

(E) Waterfall plot depicting the log2-fold change enrichment in major cell types in the primary TME between regions at the tumor core and tumor edge. Statistical significance was determined using two-sided Wilcoxon-rank sum test corrected for multiple hypothesis testing (BH) with significant results printed in yellow.

(F) Waterfall plot depicting the log2-fold change enrichment in major cell types in the metastatic LN microenvironment between regions at the tumor core and tumor edge. Statistical significance was determined using two-sided Wilcoxon-rank sum test corrected for multiple hypothesis testing (BH) with significant results printed in yellow.

(G) Stacked barplot comparing the mean cell type abundances between the aggregated primary tumor regions (core and edge) and the metastatic LN tumor core regions.

(H) Waterfall plot showing the log2-fold change enrichment in major cell types between the aggregated primary tumor microscopic regions (core and edge) and the metastatic LN tumor core regions. Statistical significance was determined using two-sided Wilcoxon-rank sum test corrected for multiple hypothesis testing (BH) with significant results printed in yellow.

(I) Heatmap summarizing the mean normalized expression of selected functional markers in cells of the T cell compartment (top left), myeloid cell compartment (top right) and stromal cells (bottom) for all primary tumor regions and the tumor core regions of metastatic LNs. Comparisons of marker expression levels within selected cell-types between tissues was performed using Wilcoxon-rank sum test adjusted for multiple hypothesis testing (BH).

(J) Violin plots show the top differentially-enriched niche neighborhoods in paired primary tumor samples (grey) or metastatic lymph node samples (orange). Barplots to the right show the proportion of samples with a niche neighborhood in the respective tissue groups.

(K) Spatial context maps depicting unique CN-CN interfaces for paired primary tumors and LN metastases. Color code from corresponding legends (right panel) applies. Spatial contexts encompassing CNs with similar cell type composition between primary tumors and paired LN metastases are highlighted: Those include a Lymphoid structure motif (blue) and a tumor border motif (grey), as well as Tumor Immune Interfaces (red).

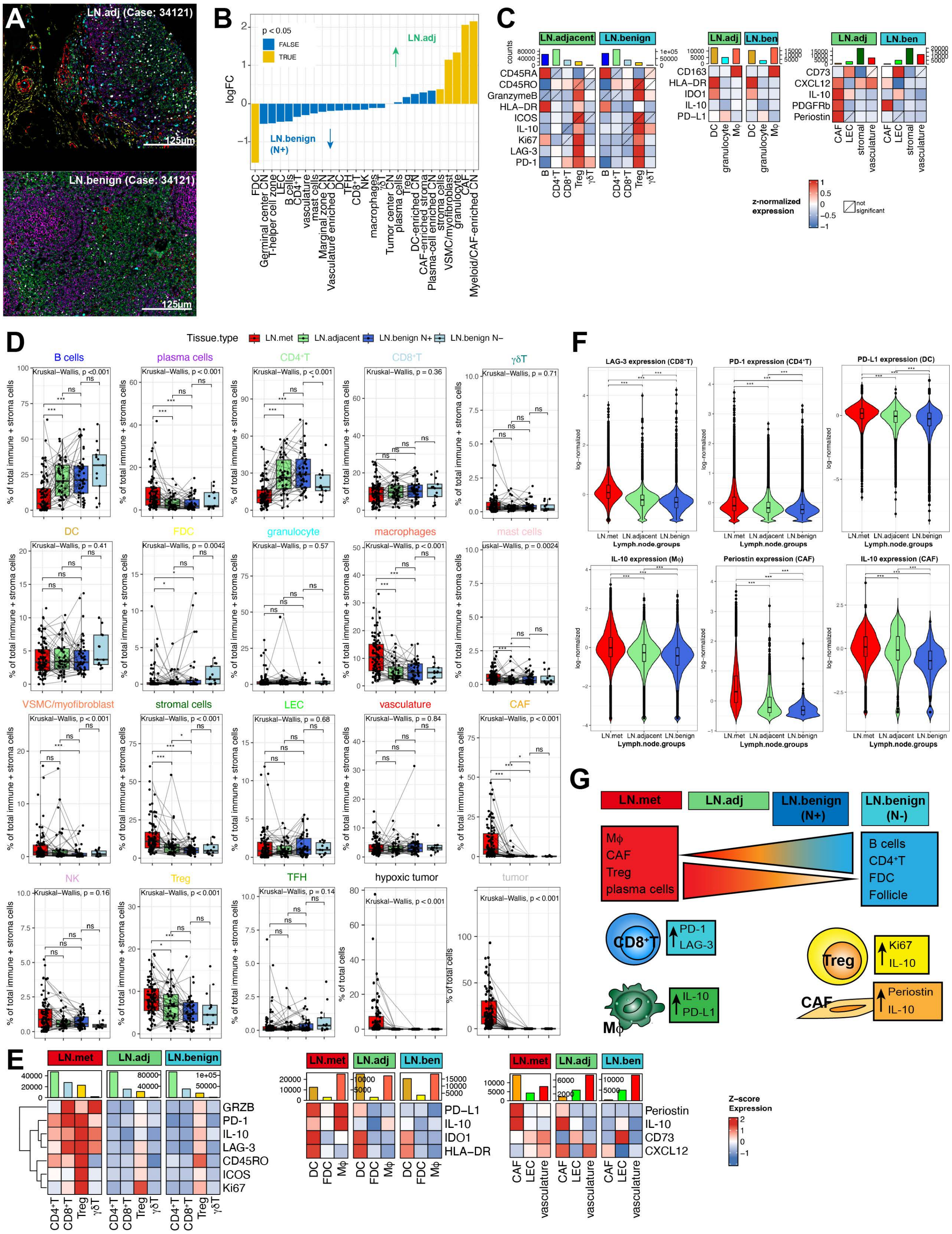
A
LN.adj (Case: 34121)
125um
LN.benign (Case: 34121)
125um
B
p < 0.05
FALSE
TRUE
LN.adj
LN.benign (N+)
logFC
FDC
Germinal center CN
T-helper cell zone
LEC
B cells
CD4+T
vasculature
mast cells
Marginal zone CN
Vasculature enriched CN
DC
TFH
CD8+T
NK
macrophages
γδT
Tumor center CN
plasma cells
Treg
DC-enriched CN
CAF-enriched stroma
Plasma-cell enriched CN
stroma cells
VSMC/myofibroblast
granulocyte
CAF
Myeloid/CAF-enriched CN
C
LN.adjacent
LN.benign
counts
CD45RA
CD45RO
GranzymeB
HLA-DR
ICOS
IL-10
Ki67
LAG-3
PD-1
B
CD4+T
CD8+T
Treg
γδT
LN.adj
LN.ben
CD163
HLA-DR
IDO1
IL-10
PD-L1
DC
granulocyte
Mφ
CD73
CXCL12
IL-10
PDGFRb
Periostin
CAF
LEC
stromal
vasculature
z-normalized expression
not significant
D
Tissue.type
LN.met
LN.adjacent
LN.benign N+
LN.benign N−
B cells
plasma cells
CD4+T
CD8+T
γδT
DC
FDC
granulocyte
macrophages
mast cells
VSMC/myofibroblast
stromal cells
LEC
vasculature
CAF
NK
Treg
TFH
hypoxic tumor
tumor
Kruskal–Wallis, p < 0.001
Kruskal–Wallis, p = 0.36
Kruskal–Wallis, p = 0.71
Kruskal–Wallis, p = 0.41
Kruskal–Wallis, p = 0.0042
Kruskal–Wallis, p = 0.57
Kruskal–Wallis, p = 0.0024
Kruskal–Wallis, p = 0.68
Kruskal–Wallis, p = 0.84
Kruskal–Wallis, p = 0.16
Kruskal–Wallis, p = 0.14
% of total immune + stroma cells
% of total cells
E
LN.met
LN.adj
LN.benign
LN.ben
GRZB
PD-1
IL-10
LAG-3
CD45RO
ICOS
Ki67
PD−L1
IL-10
IDO1
HLA−DR
Periostin
CD73
CXCL12
Z-score Expression
F
LAG-3 expression (CD8+T)
PD-1 expression (CD4+T)
PD-L1 expression (DC)
IL-10 expression (Mφ)
Periostin expression (CAF)
IL-10 expression (CAF)
log−normalized
LN.met
LN.adjacent
LN.benign
Lymph.node.groups
G
LN.met
LN.adj
LN.benign (N+)
LN.benign (N-)
Mφ
CAF
Treg
plasma cells
B cells
CD4+T
FDC
Follicle
CD8+T
PD-1
LAG-3
Treg
Ki67
IL-10
Mφ
IL-10
PD-L1
CAF
Periostin
IL-10

Figure S5: Gradual changes of single-cell features within tissues microenvironments of metastatic LNs and non-tumor-involved LNs as a correlate for distance-driven immune-evasion through LN colonization. Related to Figure 3.

(A) Representative examples of adjacent microscopically tumor-free LN regions (n=43) and non-tumor involved LNs (n=49) from paired nodal-disease positive patients.

(B) Waterfall plot highlighting the relative enrichment of CAF within adjacent microscopically tumor-free LN regions, as opposed to LN.benign.N+. Statistically significant differences between LN groups are color-coded and was determined using Wilcoxon-rank sum test adjusted for multiple hypothesis testing (BH).

(C) Heatmaps showing z-normalized expression of functional markers within cell types of the T cell (left), myeloid cell (right) and stroma compartment (center) between benign and adjacent lymph nodes in nodal-positive cases. Comparisons of marker expression levels within selected cell-types between tissues was performed using Wilcoxon-rank sum test adjusted for multiple hypothesis testing (BH).

(D) Boxplots comparing cellular abundances between LN.met (n=95), their adjacent microscopically non-involved counterparts (n=43), as well as distant benign LNs (n=49) and benign LNs (n=13) from nodal-negative cases. Comparisons between conditions were computed using Wilcoxon-rank sum. Test corrected for multiple hypothesis testing (BH). Center line of boxplots depicts median and box-limits the 25 and 75 percentiles of each condition. Whiskers are defined as 1.5x interquartile range with jitter dots depicting data from each individual region. Asterixis indicate significant results from two-sided Wilcoxon test adjusted for multiple testing, ns = not significant, *p<0.05, **p<0.01, ***p<0.001.

(E) Heatmaps depicting z-normalized expression of functional markers for selected cell types of the T cell, myeloid cell and stroma compartment between paired LN-samples. Comparisons of marker expression levels within selected cell-types between tissues was performed using Wilcoxon-rank sum test adjusted for multiple hypothesis testing (BH).

(F) Combined Violin- and boxplots depicting the mean expression of selected markers in a given cell-type between metastatic LN regions, adjacent non-tumor-infested LN regions and tumor free LNs of nodal positive patients. Statistical significance was determined using paired Wilcoxon-rank sum test adjusted for multiple hypothesis testing. Center line of boxplots depicts median and box-limits the 25 and 75 percentiles of each condition. Whiskers are defined as 1.5x interquartile range.

(G) Schematic summary of cellular and functional changes within selected cell types characteristic to tumor-involved lymph nodes and different types of non-tumor-involved LNs.

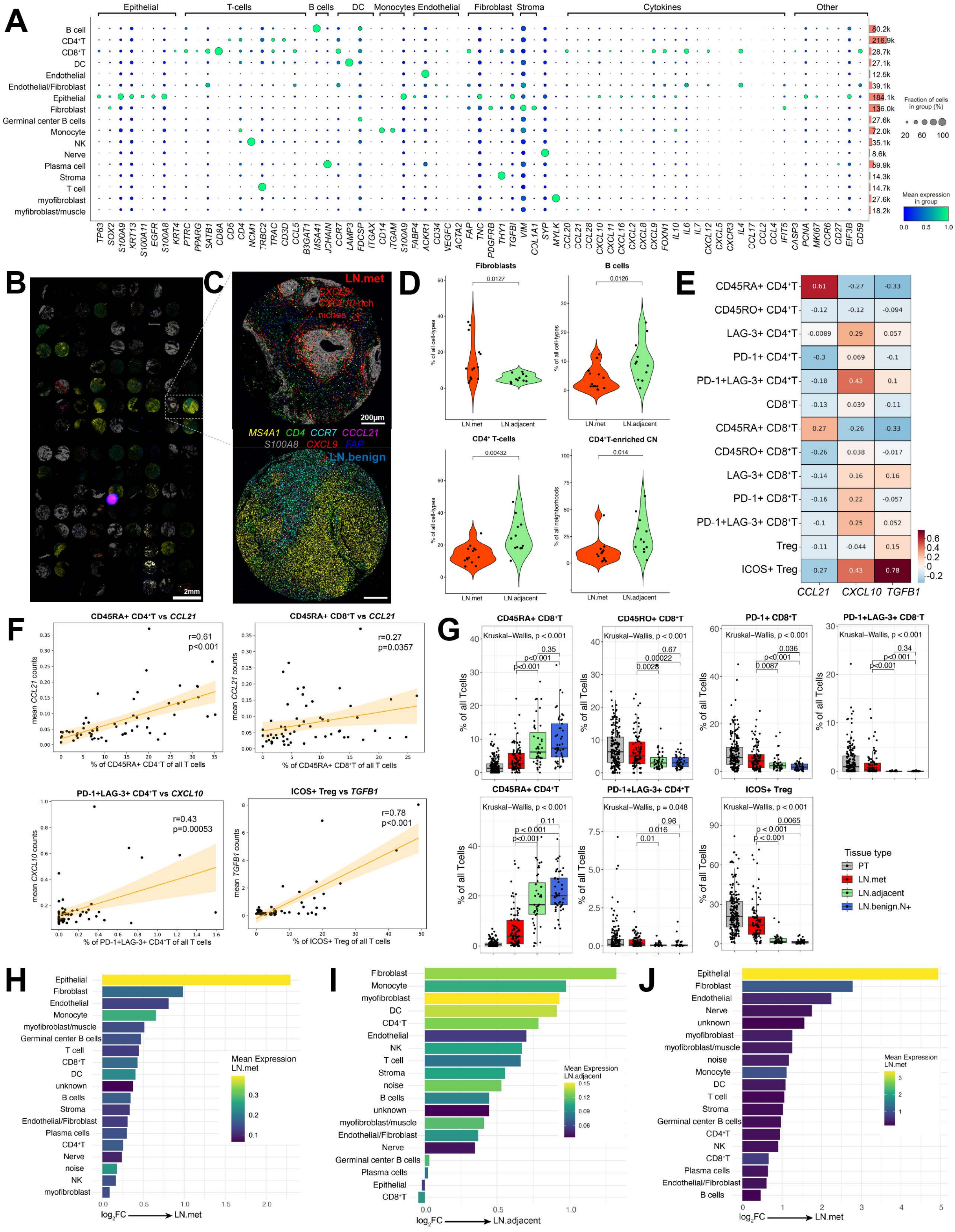

A
Epithelial
T-cells
B cells
DC
Monocytes
Endothelial
Fibroblast
Stroma
Cytokines
Other
B cell
CD4⁺T
CD8⁺T
DC
Endothelial
Endothelial/Fibroblast
Epithelial
Fibroblast
Germinal center B cells
Monocyte
NK
Nerve
Plasma cell
Stroma
T cell
myofibroblast
myfibroblast/muscle
80.2k
216.9k
28.7k
27.1k
12.5k
39.1k
184.1k
136.0k
27.6k
72.0k
35.1k
8.6k
59.9k
14.3k
14.7k
27.6k
18.2k
Fraction of cells in group (%)
20 60 100
Mean expression in group
0.0 0.5 1.0
TP63 SOX2 S100A9 KRT13 S100A11 EGFR S100A8 KRT4 PTRC PPARG SATB1 CD8A CD5 CD4 NCM1 TRBC2 TRAC CD3D CCL5 B3GAT1 MS4A1 JCHAIN CCR7 LAMP3 FDCSP ITGAX CD14 ITGAM S100A9 FABP4 ACKR1 CD34 VEGFC ACTA2 FAP TNC PDGFRB THY1 TGFBI VIM COL1A1 SYP MYLK CCL20 CCL21 CCL28 CXCL10 CXCL11 CXCL16 CXCL2 CXCL8 CXCL9 FOXN1 IL10 IL6 IL7 CXCL12 CXCL5 CXCR3 IL4 CCL17 CCL2 CCL4 IFIT5 CASP3 PCNA MKI67 CCR6 CD27 EIF3B CD59
B
2mm
C
LN.met
CXCL9/CXCL10-rich niches
200µm
MS4A1 CD4 CCR7 CCCL21
S100A8 CXCL9 FAP
LN.benign
D
Fibroblasts
0.0127
B cells
0.0126
CD4⁺ T-cells
0.00432
CD4⁺T-enriched CN
0.014
% of all cell-types
% of all neighborhoods
LN.met
LN.adjacent
E
CD45RA+ CD4⁺T
CD45RO+ CD4⁺T
LAG-3+ CD4⁺T
PD-1+ CD4⁺T
PD-1+LAG-3+ CD4⁺T
CD8⁺T
CD45RA+ CD8⁺T
CD45RO+ CD8⁺T
LAG-3+ CD8⁺T
PD-1+ CD8⁺T
PD-1+LAG-3+ CD8⁺T
Treg
ICOS+ Treg
CCL21
CXCL10
TGFB1
0.61 -0.27 -0.33
-0.12 -0.12 -0.094
-0.0089 0.29 0.057
-0.3 0.069 -0.1
-0.18 0.43 0.1
-0.13 0.039 -0.11
0.27 -0.26 -0.33
-0.26 0.038 -0.017
-0.14 0.16 0.16
-0.16 0.22 -0.057
-0.1 0.25 0.052
-0.11 -0.044 0.15
-0.27 0.43 0.78
F
CD45RA+ CD4⁺T vs CCL21
r=0.61
p<0.001
mean CCL21 counts
% of CD45RA+ CD4⁺T of all T cells
CD45RA+ CD8⁺T vs CCL21
r=0.27
p=0.0357
% of CD45RA+ CD8⁺T of all T cells
PD-1+LAG-3+ CD4⁺T vs CXCL10
r=0.43
p=0.00053
mean CXCL10 counts
% of PD-1+LAG-3+ CD4⁺T of all T cells
ICOS+ Treg vs TGFB1
r=0.78
p<0.001
mean TGFB1 counts
% of ICOS+ Treg of all T cells
G
CD45RA+ CD8⁺T
Kruskal–Wallis, p < 0.001
CD45RO+ CD8⁺T
Kruskal–Wallis, p < 0.001
PD-1+ CD8⁺T
Kruskal–Wallis, p < 0.001
PD-1+LAG-3+ CD8⁺T
Kruskal–Wallis, p < 0.001
CD45RA+ CD4⁺T
Kruskal–Wallis, p < 0.001
PD-1+LAG-3+ CD4⁺T
Kruskal–Wallis, p = 0.048
ICOS+ Treg
Kruskal–Wallis, p < 0.001
% of all Tcells
Tissue type
PT
LN.met
LN.adjacent
LN.benign.N+
H
Mean Expression LN.met
log2FC → LN.met
I
Mean Expression LN.adjacent
log2FC → LN.adjacent
J
Mean Expression LN.met
log2FC → LN.met

Figure S6: Lymph node colonization relates to enhanced *TGFB1* and IFN-γ-induced chemokine signaling via *CXCL9/CXCL10* in proximity to the tumor compartment and differential T cell infiltration. Related to Figure 3.

(A) Dotplot summarizing the mean z-normalized expression values found for within the annotated cell types of the TMA investigated using spatial transcriptomics.

(B) Overview of the spatial transcriptomics TMA. Color-code from (C) applies.

(C) Magnification of selected cores within the spatial transcriptomics dataset stratified by tumor invasion into LN.met (top) and LN.benign (bottom). Scale bars of 200μm apply to both panels.

(D) Violinplots highlighting selected differences in cell-type abundances between metastatic LN (n=29) and their adjacent benign counterparts (n=17). Wilcoxon-rank-sum test corrected for multiple-hypothesis testing (BH) was employed to determine statistical significance.

(E) Heatmap depicting correlations between the mean counts of T cell regulatory cytokines *CCL21*, *CXCL10* and *TGFB1* as assessed through spatial transcriptomics data and the T cell subsets identified through CODEX multiplex imaging within all investigated LNs. *CCL21*, *CXCL10* and *TGFB1* have been selected based on their significant enrichment within LN.met and LN.adjacent. Correlations were calculated using Pearson correlation.

(F) Scatterplots highlighting selected correlations of defined T cell subsets with mean expression levels of *CCL21*, *CXCL10* and *TGFB1* within all investigated LN regions. Statistical significance was determined using Pearson correlation analysis. Each datapoint represents a unique LN region with available data from both spatial transcriptomics and CODEX data.

(G) Boxplots summarizing mean T cell subset abundances normalized to the total T cell compartment between paired primary tumors (PT, n=125), metastatic LNs (n=95), adjacent microscopically tumor-free LN regions (n=43) and benign LNs (n=49) from nodal-positive cases. Statistical significance was determined using paired Wilcoxon-rank sum test adjusted for multiple hypothesis testing (BH) and Kruskal Wallis test. Center line of boxplots depicts median and box-limits the 25 and 75 percentiles of each condition. Whiskers are defined as 1.5x interquartile range with jitter dots depicting data from each individual region. Asterixis indicate significant results from two-sided Wilcoxon test adjusted for multiple testing, ns = not significant, *p<0.05, **p<0.01, ***p<0.001.

(H) Barplot summarizing log2-fold changes in *CXCL10* expression between metastatic LNs and adjacent benign cases. Color-code depicts average expression of the *CXCL10* within metastatic LNs.

(I) Barplot summarizing cell-type-specific log2-fold changes in *CCL21* expression between adjacent benign LNs and LN metastases. Color-code depicts average expression of the *CCL21* within adjacent benign LNs

(J) Barplot summarizing cell-type-specific log2-fold changes in *TGFBI* expression between metastatic LNs and LN.adjacent cases. Color-code depicts average expression of the *TGFBI* within metastatic LNs.

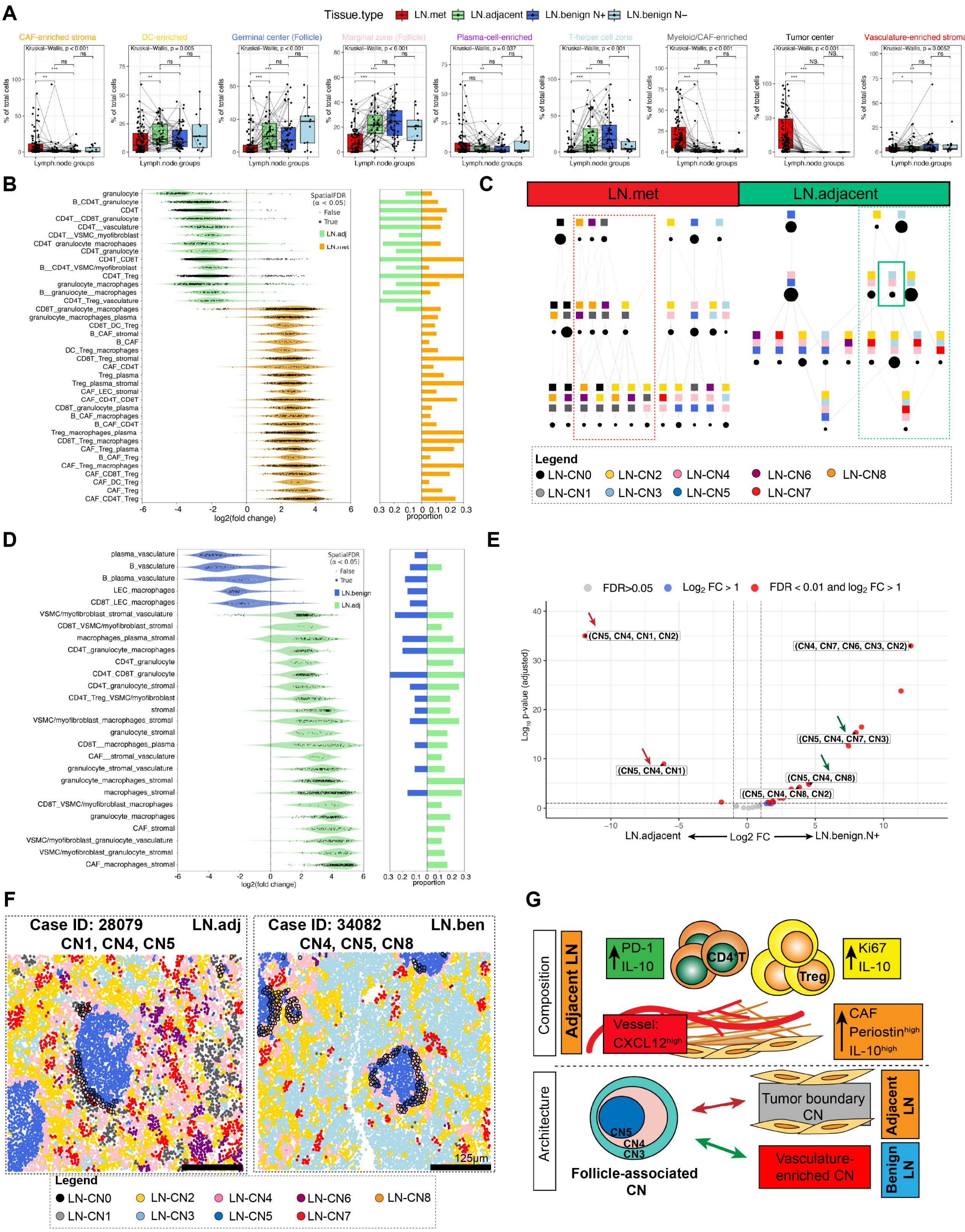
A
Tissue.type
LN.met
LN.adjacent
LN.benign N+
LN.benign N−
CAF-enriched stroma
DC-enriched
Germinal center (Follicle)
Marginal zone (Follicle)
Plasma-cell-enriched
T-helper cell zone
Myeloid/CAF-enriched
Tumor center
Vasculature-enriched stroma
% of total cells
Lymph.node.groups
B
log2(fold change)
proportion
SpatialFDR (α < 0.05)
False
True
LN.adj
LN.met
granulocyte
B_CD4T_granulocyte
CD4T
CD4T__CD8T_granulocyte
CD4T__vasculature
CD4T__VSMC_myofibroblast
CD4T_granulocyte_macrophages
CD4T_granulocyte
CD4T_CD8T
B__CD4T_VSMC/myofibroblast
CD4T_Treg
granulocyte_macrophages
B__granulocyte__macrophages
CD4T_Treg_vasculature
CD8T_granulocyte_macrophages
granulocyte_macrophages_plasma
CD8T_DC_Treg
B_CAF_stromal
B_CAF
DC_Treg_macrophages
CD8T_Treg_stromal
CAF_CD4T
Treg_plasma
Treg_plasma_stromal
CAF_LEC_stromal
CAF_CD4T_CD8T
CD8T_granulocyte_plasma
B_CAF_macrophages
B_CAF_CD4T
Treg_macrophages_plasma
CD8T_Treg_macrophages
CAF_Treg_plasma
B_CAF_Treg
CAF_Treg_macrophages
CAF_CD8T_Treg
CAF_DC_Treg
CAF_Treg
CAF_CD4T_Treg
C
LN.met
LN.adjacent
Legend
LN-CN0
LN-CN1
LN-CN2
LN-CN3
LN-CN4
LN-CN5
LN-CN6
LN-CN7
LN-CN8
D
SpatialFDR (α < 0.05)
False
True
LN.benign
LN.adj
plasma_vasculature
B_vasculature
B_plasma_vasculature
LEC_macrophages
CD8T_LEC_macrophages
VSMC/myofibroblast_stromal_vasculature
CD8T_VSMC/myofibroblast_stromal
macrophages_plasma_stromal
CD4T_granulocyte_macrophages
CD4T_granulocyte
CD4T_CD8T_granulocyte
CD4T_granulocyte_stromal
CD4T_Treg_VSMC/myofibroblast
stromal
VSMC/myofibroblast_macrophages_stromal
granulocyte_stromal
CD8T__macrophages_plasma
CAF__stromal_vasculature
granulocyte_stromal_vasculature
granulocyte_macrophages_stromal
macrophages_stromal
CD8T_VSMC/myofibroblast_macrophages
granulocyte_macrophages
CAF_stromal
VSMC/myofibroblast_granulocyte_vasculature
VSMC/myofibroblast_granulocyte_stromal
CAF_macrophages_stromal
log2(fold change)
proportion
E
FDR>0.05
Log2 FC > 1
FDR < 0.01 and log2 FC > 1
(CN5, CN4, CN1, CN2)
(CN4, CN7, CN6, CN3, CN2)
(CN5, CN4, CN7, CN3)
(CN5, CN4, CN1)
(CN5, CN4, CN8)
(CN5, CN4, CN8, CN2)
Log10 p-value (adjusted)
LN.adjacent
Log2 FC
LN.benign.N+
F
Case ID: 28079
LN.adj
CN1, CN4, CN5
Case ID: 34082
LN.ben
CN4, CN5, CN8
125μm
Legend
LN-CN0
LN-CN1
LN-CN2
LN-CN3
LN-CN4
LN-CN5
LN-CN6
LN-CN7
LN-CN8
G
Composition
Adjacent LN
PD-1
IL-10
CD4 T
Treg
Ki67
IL-10
Vessel:
CXCL12high
CAF
Periostinhigh
IL-10high
Architecture
CN5
CN4
CN3
Follicle-associated CN
Tumor boundary CN
Adjacent LN
Vasculature-enriched CN
Benign LN

Figure S7: Intersection of Follicle neighborhoods with a myeloid-enriched niche represents a distinct spatial feature of gradual re-organization within tumor involved and non-tumor involved LNs. Related to Figure 3.

(A) Boxplots comparing abundances of cellular neighborhoods between LN.met (n=95), their adjacent microscopically non-involved counterparts (n=43), as well as distant benign LNs (n=49) and benign LNs (n=13) from nodal-negative cases. Comparisons between conditions were computed using Wilcoxon-rank sum. Test corrected for multiple hypothesis testing (BH). Center line of boxplots depicts median and box-limits the 25 and 75 percentiles of each condition. Whiskers are defined as 1.5x interquartile range with jitter dots depicting data from each individual region. Asterixis indicate significant results from two-sided Wilcoxon test adjusted for multiple testing, ns = not significant, *p<0.05, **p<0.01, ***p<0.001.

(B) Violin plots show the top differentially-enriched niche neighborhoods within metastatic LN regions (orange) and adjacent non-tumor infested LN regions (green) that were statistically significant enriched (p<0.05) and present in at least 10 LN regions of the CODEX dataset. Barplots show the proportion of these cellular niches within LN.met or LN.adjacent regions.

(C) Spatial context map highlighting unique spatial contexts observed within metastatic LNs and their adjacent counterparts in the CODEX dataset. Highlighted are spatial contexts involving the CAF-enriched CN and myeloid/CAF-enriched CN1 niche. Interfaces including those niches were substantially enriched in metastatic LNs and intersected with follicle- and perifollicular T cell zone-associated niches.

(D) Violin plots show the top differentially-enriched niche neighborhoods in non-tumor infested LN regions of metastatic LNs (green) or benign LN samples (royal blue). Notably, CAFs were significantly more often present in cellular niches of adjacent LN regions, whereas the interaction of vasculature and B cells was more often detected in benign LN regions of nodal-positive patients. Barplots to the right show the proportion of samples with a niche neighborhood in the respective tissue groups

(E) Volcano-plot depicting the log2-fold enrichment of characteristic 2-chain CN-interactions within nodal-positive benign lymph nodes and LN.adjacent. Statistical comparisons were conducted in DESeq2 using all identified spatial contexts in LN.adjacent and LN.benign.N+. Selected spatial contexts highlighted by colored arrows.

(F) Representative example depicting the CN-geography map, where cells within the given spatial context are highlighted by black outline. Scale bar of 125µm applies to both panels.

(G) Schematic summarizing key cellular, functional and architectural features of adjacent lymph nodes compared to their benign counterparts

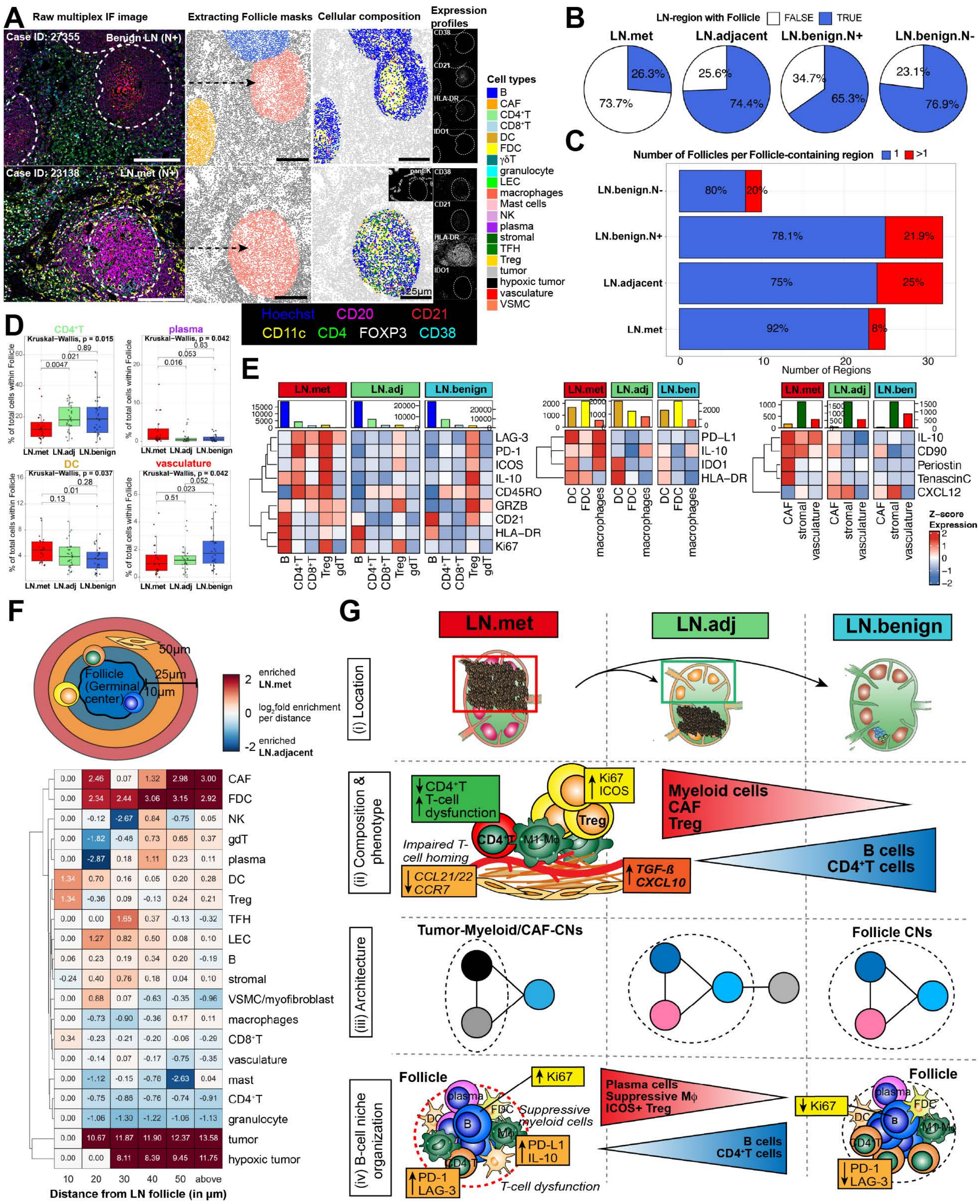

A
Raw multiplex IF image
Extracting Follicle masks
Cellular composition
Expression profiles
Case ID: 27355
Benign LN (N+)
Case ID: 23138
LN.met (N+)
CD38
CD21
HLA-DR
IDO1
panCK
25µm
Hoechst CD20 CD21
CD11c CD4 FOXP3 CD38
Cell types
B
CAF
CD4⁺T
CD8⁺T
DC
FDC
γδT
granulocyte
LEC
macrophages
Mast cells
NK
plasma
stromal
TFH
Treg
tumor
hypoxic tumor
vasculature
VSMC
B
LN-region with Follicle FALSE TRUE
LN.met
26.3%
73.7%
LN.adjacent
25.6%
74.4%
LN.benign.N+
34.7%
65.3%
LN.benign.N-
23.1%
76.9%
C
Number of Follicles per Follicle-containing region 1 >1
LN.benign.N-
80%
20%
LN.benign.N+
78.1%
21.9%
LN.adjacent
75%
25%
LN.met
92%
8%
0
10
20
30
Number of Regions
D
CD4⁺T
Kruskal–Wallis, p = 0.015
plasma
Kruskal–Wallis, p = 0.042
DC
Kruskal–Wallis, p = 0.037
vasculature
Kruskal–Wallis, p = 0.042
% of total cells within Follicle
LN.met LN.adj LN.benign
E
LN.met
LN.adj
LN.benign
LAG-3
PD-1
ICOS
IL-10
CD45RO
GRZB
CD21
HLA–DR
Ki67
B
CD4⁺T
CD8⁺T
Treg
gdT
LN.met
LN.adj
LN.ben
PD–L1
IL-10
IDO1
HLA–DR
DC
FDC
macrophages
IL-10
CD90
Periostin
TenascinC
CXCL12
CAF
stromal
vasculature
Z–score Expression
F
50µm
25µm
10µm
Follicle (Germinal center)
enriched LN.met
log₂fold enrichment per distance
enriched LN.adjacent
CAF 0.00 2.46 0.07 1.32 2.98 3.00
FDC 0.00 2.34 2.44 3.06 3.15 2.92
NK 0.00 -0.12 -2.67 0.84 -0.75 0.05
gdT 0.00 -1.82 -0.48 0.73 0.65 0.37
plasma 0.00 -2.87 0.18 1.11 0.23 0.11
DC 1.34 0.70 0.16 0.05 0.20 0.28
Treg 1.34 -0.36 0.09 -0.13 0.24 0.21
TFH 0.00 0.00 1.65 0.37 -0.13 -0.32
LEC 0.00 1.27 0.82 0.50 0.08 0.10
B 0.06 0.23 0.19 0.34 0.20 -0.19
stromal -0.24 0.40 0.76 0.18 0.04 0.10
VSMC/myofibroblast 0.00 0.88 0.07 -0.63 -0.35 -0.96
macrophages 0.00 -0.73 -0.90 -0.36 0.17 0.11
CD8⁺T 0.34 -0.23 -0.21 -0.20 -0.06 -0.29
vasculature 0.00 -0.14 0.07 -0.17 -0.75 -0.35
mast 0.00 -1.12 -0.15 -0.78 -2.63 0.04
CD4⁺T 0.00 -0.75 -0.88 -0.76 -0.74 -0.91
granulocyte 0.00 -1.06 -1.30 -1.22 -1.06 -1.13
tumor 0.00 10.67 11.87 11.90 12.37 13.58
hypoxic tumor 0.00 0.00 8.11 8.39 9.45 11.75
10 20 30 40 50 above
Distance from LN follicle (in µm)
G
LN.met
LN.adj
LN.benign
(i) Location
(ii) Composition & phenotype
CD4⁺T
T-cell dysfunction
Ki67
ICOS
Treg
CD4⁺T
M1-Mϕ
Impaired T-cell homing
CCL21/22
CCR7
TGF-ß
CXCL10
Myeloid cells
CAF
Treg
B cells
CD4⁺T cells
(iii) Architecture
Tumor-Myeloid/CAF-CNs
Follicle CNs
(iv) B-cell niche organization
Follicle
Ki67
plasma
FDC
Suppressive myeloid cells
PD-L1
IL-10
PD-1
LAG-3
T-cell dysfunction
Plasma cells
Suppressive Mϕ
ICOS+ Treg
B cells
CD4⁺T cells
Ki67
Follicle
plasma
FDC
DC
B
CD4⁺T
M1-Mϕ
PD-1
LAG-3

Figure S8: Distance-driven immunomodulation of LN-microenvironments is reflected by gradual changes in cellular composition and interaction within LN-follicles. Related to Figure 4.

(A) Analytical steps to extract and dissect all lymph node follicles within the HNSCC dataset, including

(i) extracting the follicle mask in ImageJ,

(ii) assessing the cellular composition and

(iii) functional marker expression therein and in its proximity. Scale bars of 125μm apply to all panels.

(B) Piecharts depicting whether the investigated LN region contained at least one lymph node follicle. Results are stratified by LN categories.

(C) Stacked barplots depicting the number of LN follicles per investigated LN region amongst all regions that contained at least one LN follicle.

(D) Selected boxplots comparing cell type abundance across LN.met (n=95), LN.adjacent (n=43) and LN.benign (n=49) within patients with nodal metastasis. Statistical significance between groups was determined using Wilcoxon-rank sum test corrected for multiple hypothesis testing (BH). Center line of boxplots depicts median and box-limits the 25 and 75 percentiles of each condition. Whiskers are defined as 1.5x interquartile range with jitter dots depicting data from each individual region. Asterixis indicate significant results from two-sided Wilcoxon test adjusted for multiple testing, ns = not significant, *p<0.05, **p<0.01, ***p<0.001.

(E) Marker expression within distinct cell types between LN-sites of nodal-positive patients. Comparisons of marker expression levels within selected cell-types between tissues was performed using Wilcoxon-rank sum test adjusted for multiple hypothesis testing (BH).

(F) Schematic summarizing the analytical approach to calculate the cell-type enrichment in the vicinity of the LN follicle (top) with Heatmap comparing the log2-fold change in cell-type enrichment between metastatic and adjacent LN-regions of nodal-positive patients clustered by cell-types.

(G) Proposed model on the re-organization of cellular (ii) and architectural features (iii) that occur within and across LN.met regions, adjacent microscopically non-involved regions of LN-regions and the non-tumor-involved LNs of nodal-positive patients. In addition to global changes within the TME, we also detected distinct compositional and phenotypical changes within B cell-enriched structures (iv) between nodal-positive and nodal-negative patients, including an enrichment of dysfunctional T cells, suppressive myeloid cells and a paradoxical higher proliferation of B cells within Follicles of nodal-positive patients.

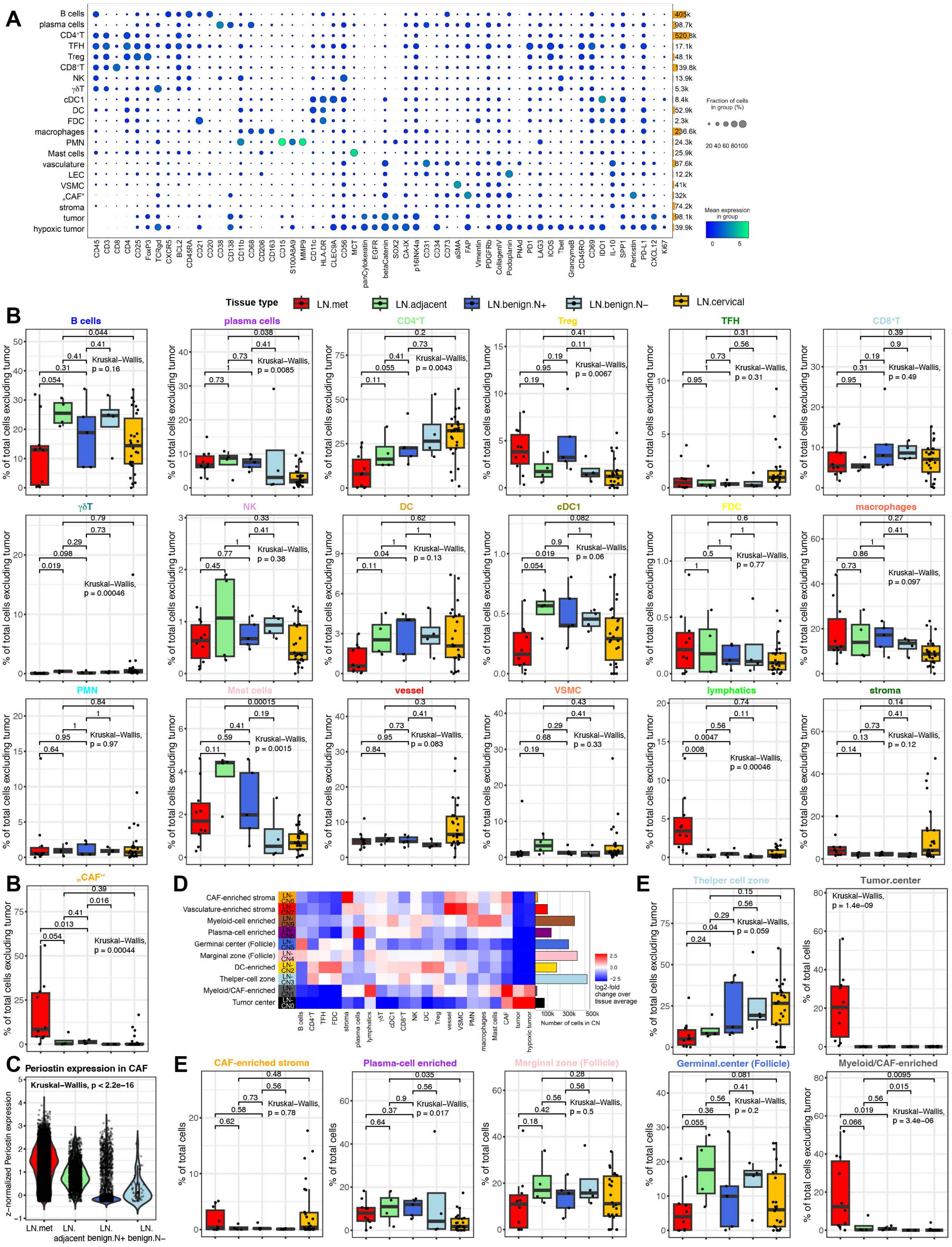

Figure S9: Compositional and architectural differences between tumor-infested, non-tumor involved and benign LNs assessed by CODEX multiplex imaging. Related to Figure 5.

(A) Dotplot summarizing mean normalized marker expression amongst all major cell types identified within the HNSCC and normal LN validation cohort.

(B) Boxplots summarizing the mean cellular abundances outside of the tumor compartment within each LN region (LN.met, n=10; LN.adjacent, n=4; LN.benign.N+, n=5; LN.benign.N-, n=4 and LN.cervical, n=26) stratified by all investigated LN types. Statistical significance was determined using pairwise Wilcoxon-rank sum test adjusted for multiple hypothesis testing (BH) and Kruskal-Wallis rank sum testing. Center line of boxplots depicts median and box-limits the 25 and 75 percentiles of each condition. Whiskers are defined as 1.5x interquartile range with jitter dots depicting data from each individual region.

(C) Violinplot comparing the mean expression of periostin amongst all CAFs stratified by the different regions of cancer patients. Statistical significance was determined using Kruskal Wallis rank sum test.

(D) Identification of 10 unique cellular neighborhoods in all investigated LN-samples based on the k-nearest neighbors =15 of all major cell types with corresponding total cell abundances depicted as color-coded barcharts.

(E) Boxplots summarizing the mean CN abundances within each LN region stratified by all investigated LN types. Statistical significance was determined using pairwise Wilcoxon-rank sum test adjusted for multiple hypothesis testing (BH) and Kruskal-Wallis rank sum testing. Center line of boxplots depicts median and box-limits the 25 and 75 percentiles of each condition. Whiskers are defined as 1.5x interquartile range with jitter dots depicting data from each individual region.

Asterixis indicate significant results from two-sided Wilcoxon test adjusted for multiple testing, ns = not significant, *p<0.05, **p<0.01, ***p<0.001.

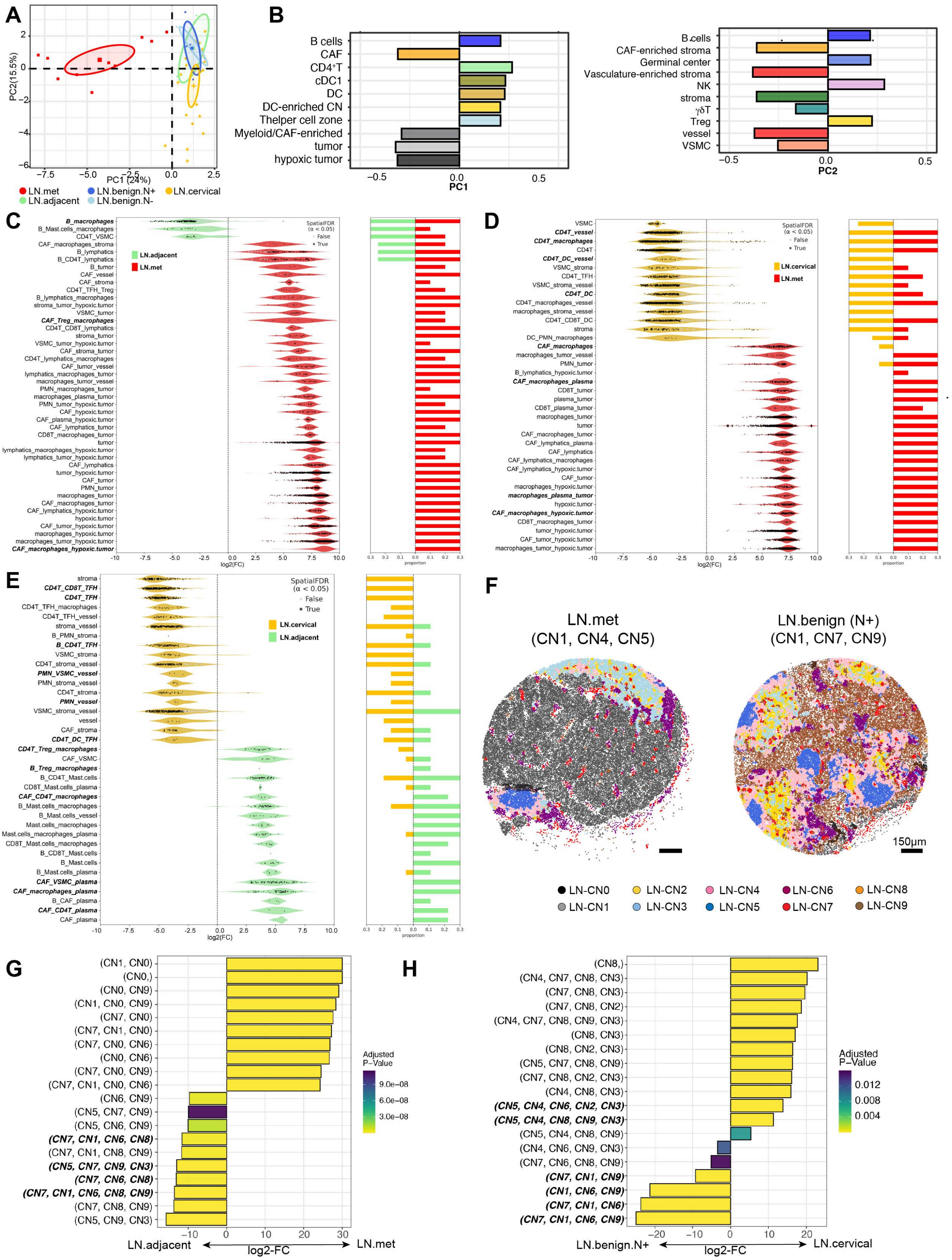

A
PC1 (24%)
PC2(15.5%)
LN.met
LN.benign.N+
LN.cervical
LN.adjacent
LN.benign.N-
B
B cells
CAF
CD4+T
cDC1
DC
DC-enriched CN
Thelper cell zone
Myeloid/CAF-enriched
tumor
hypoxic tumor
PC1
B cells
CAF-enriched stroma
Germinal center
Vasculature-enriched stroma
NK
stroma
γδT
Treg
vessel
VSMC
PC2
C
SpatialFDR (α < 0.05)
False
True
LN.adjacent
LN.met
log2(FC)
proportion
D
LN.cervical
LN.met
E
LN.cervical
LN.adjacent
F
LN.met (CN1, CN4, CN5)
LN.benign (N+) (CN1, CN7, CN9)
150μm
LN-CN0
LN-CN1
LN-CN2
LN-CN3
LN-CN4
LN-CN5
LN-CN6
LN-CN7
LN-CN8
LN-CN9
G
(CN1, CN0)
(CN0,)
(CN0, CN9)
(CN1, CN0, CN9)
(CN7, CN0)
(CN7, CN1, CN0)
(CN7, CN0, CN6)
(CN0, CN6)
(CN7, CN0, CN9)
(CN7, CN1, CN0, CN6)
(CN6, CN9)
(CN5, CN7, CN9)
(CN5, CN6, CN9)
(CN7, CN1, CN6, CN8)
(CN7, CN1, CN8, CN9)
(CN5, CN7, CN9, CN3)
(CN7, CN6, CN8)
(CN7, CN1, CN6, CN8, CN9)
(CN7, CN8, CN9)
(CN5, CN9, CN3)
Adjusted P-Value
9.0e−08
6.0e−08
3.0e−08
LN.adjacent
log2-FC
LN.met
H
(CN8,)
(CN4, CN7, CN8, CN3)
(CN7, CN8, CN3)
(CN7, CN8, CN2)
(CN4, CN7, CN8, CN9, CN3)
(CN8, CN3)
(CN8, CN2, CN3)
(CN5, CN7, CN8, CN9)
(CN7, CN8, CN2, CN3)
(CN4, CN8, CN3)
(CN5, CN4, CN6, CN2, CN3)
(CN5, CN4, CN8, CN9, CN3)
(CN5, CN4, CN8, CN9)
(CN4, CN6, CN9, CN3)
(CN7, CN6, CN8, CN9)
(CN7, CN1, CN9)
(CN1, CN6, CN9)
(CN7, CN1, CN6)
(CN7, CN1, CN6, CN9)
Adjusted P-Value
0.012
0.008
0.004
LN.benign.N+
log2-FC
LN.cervical

Figure S10: Cellular and organizational differences between tumor-infested, non-tumor involved and benign LNs assessed by CODEX multiplex imaging. Related to Figure 5.

(A) Results from principal-component analysis using major cell type abundances within all investigated tissue regions of the HNSCC CODEX validation dataset. Individual samples are colored according to tissue type. Ellipses represent 95% confidence interval for each tissue type based on multivariate normal distribution assumption.

(B) Barplot depicting the relative contribution of features to PC1 and PC2 shown in (A).

(C) Violin plots showing the top differentially-enriched cellular niche neighborhoods in LN.met (red) and LN.adjacent (light green). Barplots to the right show the proportion of samples with a niche neighborhood in the respective tissue groups. Selected cellular niche networks involving CAF-macrophage interactions highlighted in bold.

(D) Violin plots showing the top differentially-enriched cellular niche neighborhoods in LN.met (red) and benign cervical LNs of non-cancer patients (orange). Barplots to the right show the proportion of samples with a niche neighborhood in the respective tissue groups. Selected cellular niche networks involving CAF-macrophage interactions highlighted in bold.

(E) Violin plots showing the top differentially-enriched cellular niche neighborhoods in non-tumor-infested adjacent LN regions (light green) and benign cervical LNs of non-cancer patients (orange). Barplots to the right show the proportion of samples with a niche neighborhood in the respective tissue groups. Selected cellular niche networks involving CAF-macrophage interactions highlighted in bold.

(F) Cellular neighborhood geography map depicting the spatial distribution of cells and their corresponding CNs within a metastatic LN (top) and its paired LN.benign. Spatial contexts involving the intersection of CN1, CN4 and CN5 (LN.met; top) and CN1, CN7, CN9 (LN.benign.N+) are highlighted by black boundaries. Scale bar of 150µm applies to both panels.

(G) Barplot comparing the log2-fold change of the top 10 spatial contexts found in metastatic LNs compared to non-tumor infested adjacent LN regions color-coded by significance. Statistical significance was determined using differential abundance testing in DESeq2 package.

(H) Barplot comparing the log2-fold changes of the top spatial contexts found in benign LNs of nodal-positive patients compared to benign LNs of non-cancer patients color-coded by significance. Statistical significance was determined using differential abundance testing in DESeq2 package.

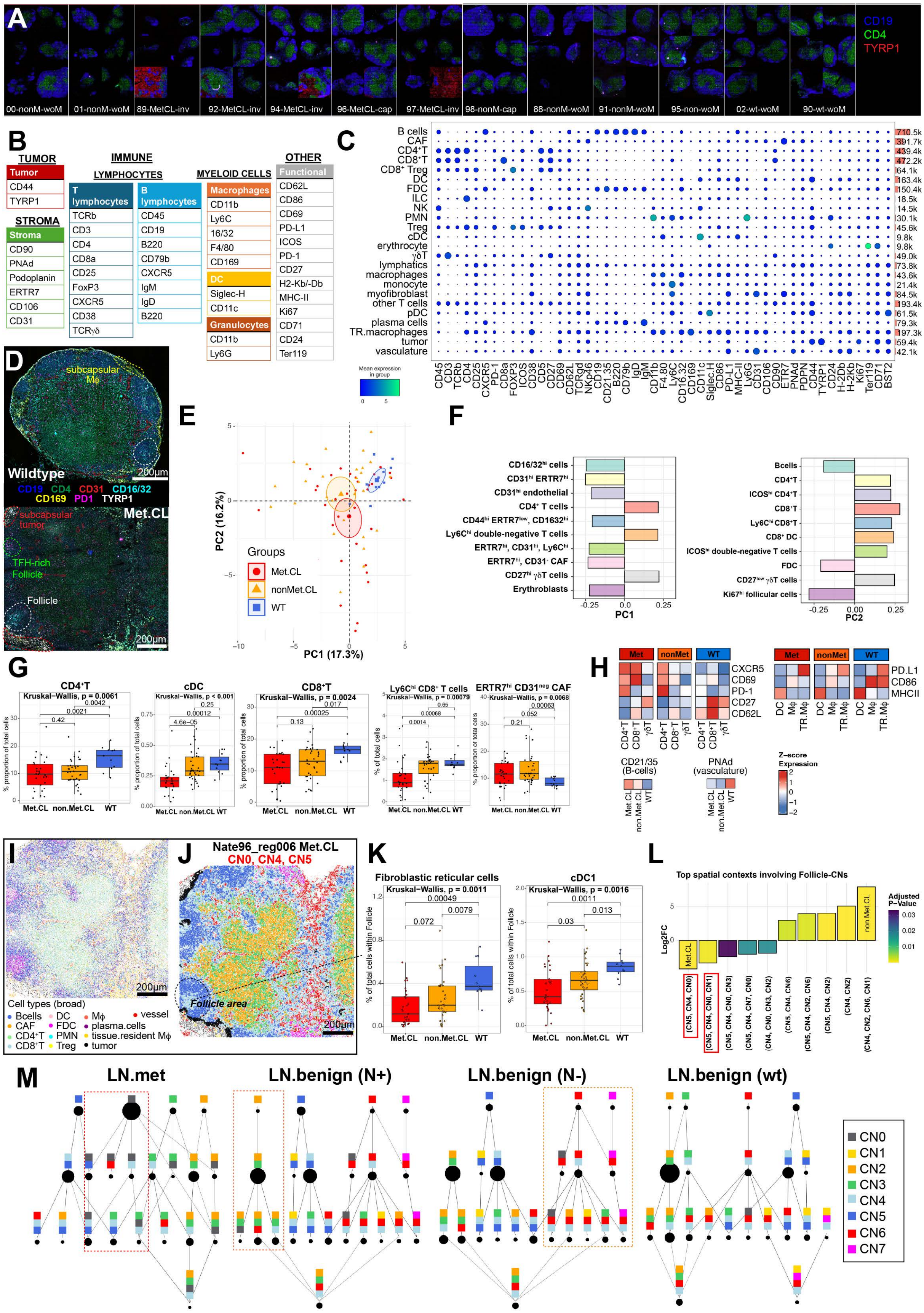
A
CD19
CD4
TYRP1
00-nonM-woM
01-nonM-woM
89-MetCL-inv
92-MetCL-inv
94-MetCL-inv
96-MetCL-cap
97-MetCL-inv
98-nonM-cap
88-nonM-woM
91-nonM-woM
95-non-woM
02-wt-woM
90-wt-woM
B
TUMOR
Tumor
CD44
TYRP1
STROMA
Stroma
CD90
PNAd
Podoplanin
ERTR7
CD106
CD31
IMMUNE
LYMPHOCYTES
T lymphocytes
TCRb
CD3
CD4
CD8a
CD25
FoxP3
CXCR5
CD38
TCRγδ
B lymphocytes
CD45
CD19
B220
CD79b
CXCR5
IgM
IgD
B220
MYELOID CELLS
Macrophages
CD11b
Ly6C
16/32
F4/80
CD169
DC
Siglec-H
CD11c
Granulocytes
CD11b
Ly6G
OTHER
Functional
CD62L
CD86
CD69
PD-L1
ICOS
PD-1
CD27
H2-Kb/-Db
MHC-II
Ki67
CD71
CD24
Ter119
C
B cells
CAF
CD4⁺T
CD8⁺T
CD8⁺ Treg
DC
FDC
ILC
NK
PMN
Treg
cDC
erythrocyte
γδT
lymphatics
macrophages
monocyte
myofibroblast
other T cells
pDC
plasma cells
TR.macrophages
tumor
vasculature
710.5k
391.7k
439.4k
472.2k
64.1k
163.4k
150.4k
18.5k
14.5k
30.1k
45.6k
9.8k
9.8k
49.0k
73.8k
43.6k
21.4k
84.5k
193.4k
61.5k
79.3k
197.3k
59.4k
42.1k
Mean expression in group
CD45 CD3 TCRb CD4 CD25 CXCR5 PD1 CD8a FOXP3 ICOS CD38 CD27 CD69 CD62L TCRgd NK46 CD19 CD21.35 B220 CD79b IgD IgM CD11b F4.80 Ly6C CD16.32 CD169 CD11c Siglec.H CD86 PD-L1 MHC-II Ly6G CD31 CD106 CD90 ERTR7 PNAd PDPN CD44 TYRP1 CD24 H-2Db H-2Kb Ki67 Ter119 CD71 BST2
D
subcapsular Mφ
Wildtype
200µm
CD19 CD4 CD31 CD16/32
CD169 PD1 TYRP1
subcapsular tumor
Met.CL
TFH-rich Follicle
Follicle
200µm
E
PC2 (16.2%)
PC1 (17.3%)
Groups
Met.CL
nonMet.CL
WT
F
CD16/32hi cells
CD31hi ERTR7hi
CD31hi endothelial
CD4⁺ T cells
CD44hi ERTR7low, CD1632hi
Ly6Chi double-negative T cells
ERTR7hi, CD31hi, Ly6Chi
ERTR7hi, CD31⁻ CAF
CD27hi γδT cells
Erythroblasts
PC1
Bcells
CD4⁺T
ICOShi CD4⁺T
CD8⁺T
Ly6Chi CD8⁺T
CD8⁺ DC
ICOShi double-negative T cells
FDC
CD27low γδT cells
Ki67hi follicular cells
PC2
G
CD4⁺T
Kruskal–Wallis, p = 0.0061
cDC
Kruskal–Wallis, p < 0.001
CD8⁺T
Kruskal–Wallis, p = 0.0024
Ly6Chi CD8⁺ T cells
Kruskal–Wallis, p = 0.00079
ERTR7hi CD31neg CAF
Kruskal–Wallis, p = 0.0068
% proportion of total cells
Met.CL non.Met.CL WT
H
Met nonMet WT
CXCR5
CD69
PD-1
CD27
CD62L
CD4⁺T CD8⁺T γδT
PD.L1
CD86
MHCII
DC Mφ TR.Mφ
CD21/35 (B-cells)
PNAd (vasculature)
Z-score Expression
I
200µm
Cell types (broad)
Bcells
CAF
CD4⁺T
CD8⁺T
DC
FDC
PMN
Treg
Mφ
plasma.cells
tissue.resident Mφ
tumor
vessel
J
Nate96_reg006 Met.CL
CN0, CN4, CN5
Follicle area
200µm
K
Fibroblastic reticular cells
Kruskal–Wallis, p = 0.0011
cDC1
Kruskal–Wallis, p = 0.0016
% of total cells within Follicle
Met.CL non.Met.CL WT
L
Top spatial contexts involving Follicle-CNs
Log2FC
Adjusted P-Value
Met.CL
nonMet.CL
(CN5, CN4, CN0)
(CN5, CN4, CN0, CN1)
(CN5, CN4, CN0, CN3)
(CN5, CN4, CN7, CN0)
(CN4, CN0, CN3, CN2)
(CN5, CN4, CN6)
(CN5, CN4, CN2, CN6)
(CN5, CN4, CN2)
(CN4, CN2)
(CN4, CN2, CN6, CN1)
M
LN.met
LN.benign (N+)
LN.benign (N-)
LN.benign (wt)
CN0
CN1
CN2
CN3
CN4
CN5
CN6
CN7

Figure S11: A murine model of lymph-node metastatic B16F0-melanoma reveals cellular and architectural re-organization of LNs following tumor-inoculation. Related to Figure 5.

(A) Overview of the lymph nodes investigated in the B16F0-melanoma mouse model, highlighting the location of lymph node metastasis within TDLN through raw immunofluorescence imaging.

(B) Summary of the marker panel used for multiplex imaging-based investigation of the lymph node microenvironment between the mouse groups.

(C) Dotplot summarizing mean expression of markers in the broad cell types investigated in this study

(D) Representative images of a benign lymph node in wildtype-mice and a lymph node with subcapsular tumor infiltrate from a metastatic B16F0-cell line (bottom).

(E) Results from principal-component analysis using major cell type abundances within all investigated regions of the *in-vivo* LN metastasis experiment. Individual samples are colored according to mouse group (met.CL, n=29 samples; nonMet.CL, n=34 samples; WT, n=11 samples). Ellipses represent 95% confidence interval for each mouse group based on multivariate normal distribution assumption.

(F) Bar plot showing contribution of single cell types to the PCs shown in (E).

(G) Boxplots comparing cellular abundance of cell types significantly associated with a distinct Cell-line group. Statistical significance was determined using Wilcoxon-rank sum test corrected for multiplex hypothesis testing (BH). Center line of boxplots depicts median and box-limits the 25 and 75 percentiles of each condition. Whiskers are defined as 1.5x interquartile range with jitter dots depicting data from each individual region.

(H) Heatmaps summarizing z-normalized expression within selected cell types between the mouse groups. Comparisons of marker expression levels within selected cell-types between tissues was performed using Wilcoxon-rank sum test adjusted for multiple hypothesis testing (BH).

(I) Cell type geography map showing broad cell types within the metastatic lymph node shown in (D, bottom).

(J) Cellular neighborhood geography map for the lymph node shown in (I) while highlighting the spatial intersection of CN0, CN4 and CN5 by black outlines. Color code defined in (M) applies.

(K) Boxplots revealing a significant depletion of $CD8^+$ T cells and $Ly6C^+$ memory T cells in particular within Follicle-associated CNs (CN4 and CN5) of mice inoculated with the metastatic B16-F0 melanoma cell line. Alongside, a relative depletion of fibroblastic reticular cells within LN-metastatic mice was being observed (right). Center line of boxplots depicts median and box-limits the 25 and 75 percentiles of each condition. Whiskers are defined as 1.5x interquartile range with jitter dots depicting data from each individual region.

(L) Barplots depicting the enrichment of the top 10 distinct spatial contexts by log2-fold change between samples from mice inoculated with the metastatic cell line (n=29) and samples from mice inoculated with non-metastatic B16F0 melanoma cells (n=34) with statistical significance provided as color-code. Statistical comparisons were performed using differential enrichment analysis in DESeq2.

(M) Spatial context maps depicting the top CN-CN interfaces detected in metastatic LN regions (n=5) (left), non tumor-involved LNs of nodal-positive mice (n=4) (center left), non-tumor involved LNs of nodal-negative mice (n=28) (center right) and benign LN (n=11) (right). Color code to the right applies.

Abbreviations: AL = axillary left; AR = axillary right; BL = brachial left; CL = Cell-line.

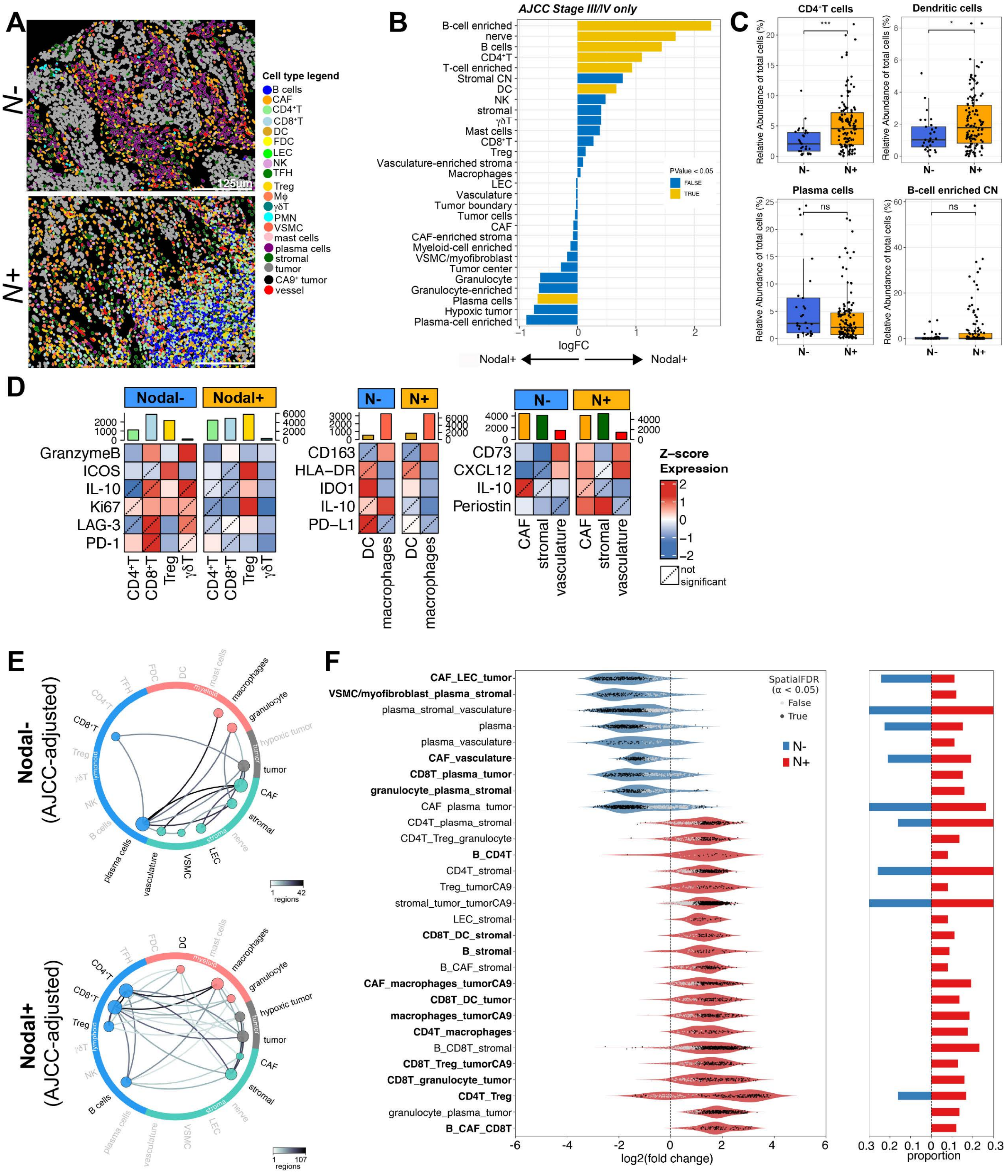

A
N-
N+
Cell type legend
B cells
CAF
CD4⁺T
CD8⁺T
DC
FDC
LEC
NK
TFH
Treg
Mφ
γδT
PMN
VSMC
mast cells
plasma cells
stromal
tumor
CA9⁺ tumor
vessel
B
AJCC Stage III/IV only
logFC
Nodal+
Nodal+
PValue < 0.05
FALSE
TRUE
C
CD4⁺T cells
Dendritic cells
Plasma cells
B-cell enriched CN
Relative Abundance of total cells (%)
D
Nodal-
Nodal+
N-
N+
Z-score Expression
not significant
E
Nodal- (AJCC-adjusted)
Nodal+ (AJCC-adjusted)
regions
F
SpatialFDR (α < 0.05)
log2(fold change)
proportion

Figure S12: Validation of cellular, phenotypical and spatial features within the primary TME linked to nodal disease corrected for tumor stage. Related to Figure 7.

(A) Representative cell-type geography maps highlighting the differences in cellular composition of the primary TME in patients with or without nodal disease and ≥T3 HNSCC.

(B) Waterfall plot showing the log2-fold change enrichment in major cell types and cellular neighborhoods in the primary tumor regions of patients with AJCC stage III/IV tumors stratified by nodal disease status (N+, n=122; N-, n=29). Statistical significance was determined using two-sided Wilcoxon-rank sum test corrected for multiple hypothesis testing (BH) with significant results printed in yellow.

(C) Boxplots highlighting the differences in relative abundances of selected cell-types and cellular neighborhoods in patients with AJCC stage III/IV tumors stratified by nodal disease status. Statistical significance was determined using Wilcoxon rank sum test adjusted for multiple hypothesis testing (BH). Center line of boxplots depicts median and box-limits the 25 and 75 percentiles of each condition. Whiskers are defined as 1.5x interquartile range with jitter dots depicting data from each individual region. Asterixis indicate significant results from two-sided Wilcoxon test adjusted for multiple testing, ns = not significant, *p<0.05, **p<0.01, ***p<0.001.

(D) Heatmap summarizing the mean normalized expression of selected functional markers in cells of the T cell compartment (left), myeloid cell compartment (center) and stromal cells (right) for patients with AJCC stage III/IV disease stratified by nodal disease status. Barplots depict the total cell counts of the corresponding cell-types in the nodal disease condition. Comparisons of marker expression levels within selected cell-types between tissues was performed using Wilcoxon-rank sum test adjusted for multiple hypothesis testing (BH).

(E) Circos plot depicting characteristic cell-cell interactions in primary tumor regions of patients with (n=125) or without (n=62) nodal disease adjusted for AJCC stage. Nodes represent cell types within cancer border-associated niches, and edge weights correspond to the number of unique regions with the corresponding interaction. Node size is proportional to connectedness, as measured by eigenvector centrality.

(F) Violin plots show the top differentially-enriched niche neighborhoods within in nodal-negative (blue) and nodal-positive patients adjusted for AJCC-stage with p<0.05 (left). Bold cellular niches indicate local niches enriched in cells that were significantly enriched in either nodal disease condition (i.e. niches involving plasma cells in nodal-negative patients or macrophages, $CD4^{+}T$ and CAFs in nodal-positive patients). Barplots show the proportion of these cellular niches within the primary TME of nodal-negative and nodal-positive patient regions.

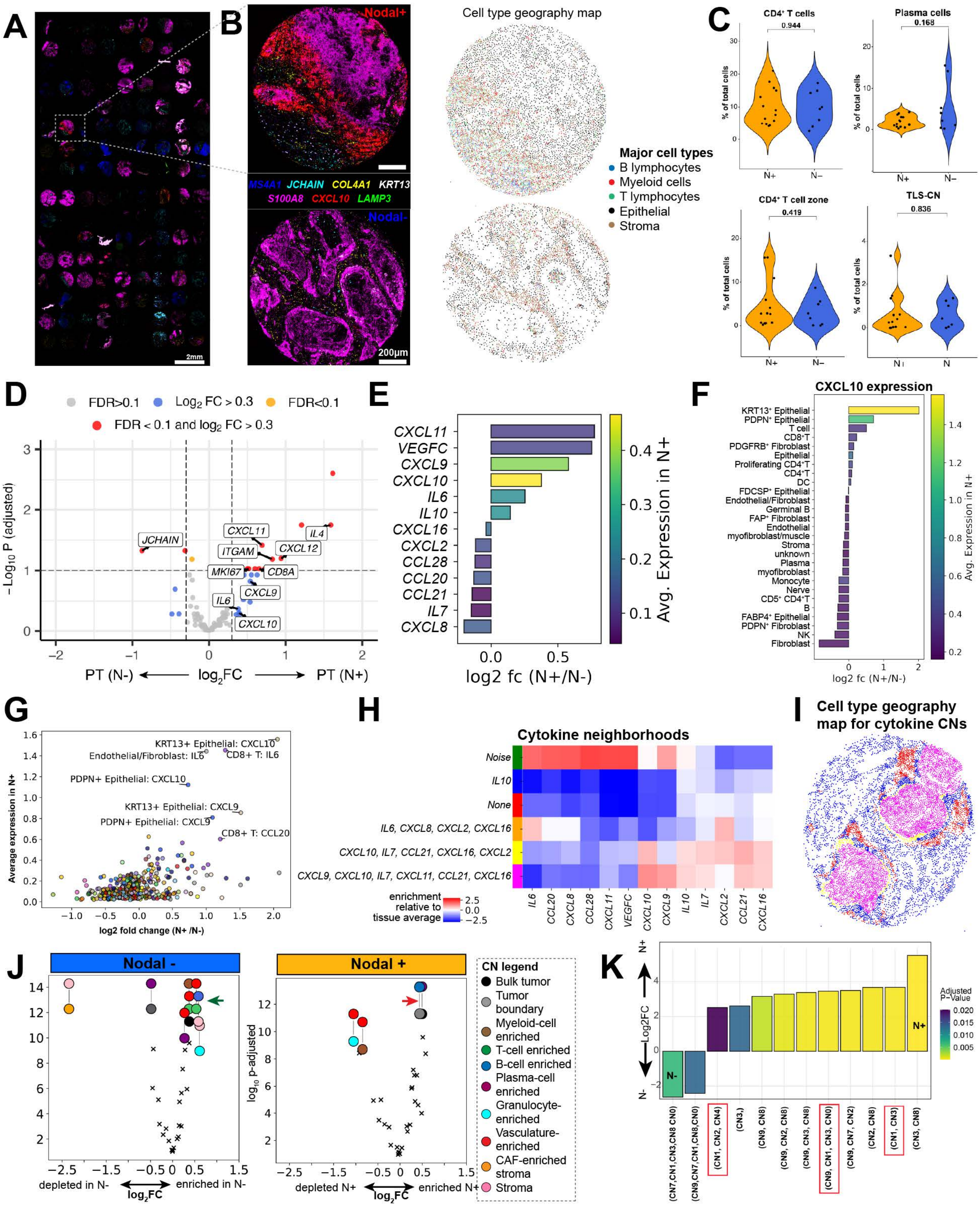

A
B
Nodal+
Nodal-
MS4A1 JCHAIN COL4A1 KRT13
S100A8 CXCL10 LAMP3
2mm
200µm
Cell type geography map
Major cell types
B lymphocytes
Myeloid cells
T lymphocytes
Epithelial
Stroma
C
CD4+ T cells
Plasma cells
CD4+ T cell zone
TLS-CN
% of total cells
D
FDR>0.1
Log2 FC > 0.3
FDR<0.1
FDR < 0.1 and log2 FC > 0.3
−Log10 P (adjusted)
PT (N-)
log2FC
PT (N+)
E
log2 fc (N+/N-)
Avg. Expression in N+
F
CXCL10 expression
log2 fc (N+/N-)
Avg. Expression in N+
G
Average expression in N+
log2 fold change (N+ /N-)
H
Cytokine neighborhoods
enrichment relative to tissue average
I
Cell type geography map for cytokine CNs
J
Nodal -
Nodal +
depleted in N-
enriched in N-
depleted N+
enriched N+
log2FC
log10 p-adjusted
CN legend
Bulk tumor
Tumor boundary
Myeloid-cell enriched
T-cell enriched
B-cell enriched
Plasma-cell enriched
Granulocyte-enriched
Vasculature-enriched
CAF-enriched stroma
Stroma
K
Log2FC
Adjusted P-Value

Figure S13: Enrichment of IFN-γ induced chemokines within primary tumors of nodal-disease positive cases associates with T cell recruitment and enhanced inflammatory signaling. Related to Figure 7.

(A) Overview of the investigated spatial transcriptomics TMA. Color-code from (B) apply.

(B) Magnification of selected cores within the spatial transcriptomics dataset stratified by nodal disease (left) with corresponding cell type geography maps for major cell types identified within the TMA cores. Scale bars of 200µm apply to both subpanels.

(C) Boxplots depicting cell type (top) and cell-neighborhood proportions (bottom) between nodal+ and nodal-negative cases within the ST dataset

(D) Volcano-plot showing results from differential enrichment analysis using pseudo bulk data from the orthogonal spatial transcriptomics dataset. Selected genes implicated in immune-cell recruitment and interferon-γ-response genes are highlighted.

(E) Barplot summarizing log2-fold changes in cytokine/chemokine expression between PT-regions of nodal-positive (n=38) and nodal-negative cases (n=18). Color-code depicts average expression of the corresponding cytokine in nodal-positive cases.

(F) Barplot summarizing log2-fold changes in cell-type specific *CXCL10*-expression between PT-regions of nodal-positive and nodal-negative cases. Color-code depicts average expression of the corresponding cytokine in nodal-positive cases.

(G) Volcano plot depicting the enrichment of cell-type specific cytokines between nodal-positive and nodal-negative cases assessed in PT-regions.

(H) Heatmap highlighting the relative expression of cytokine and chemokines within a given cytokine neighborhood.

(I) Cytokine neighborhood map, showcasing the spatial distribution of cytokine neighborhoods. Color-code from (H) apply.

(J) Volcano-plots depicting the enrichment of characteristic 2-chain CN-interactions within nodal-negative (n=62) and nodal-positive primary tumors (n=125) as compared to a random null-distribution. Interaction of T cell enriched-CN3 with the vasculature-enriched CN7 highlighted for nodal-negative patients (left, green arrow). Interaction of B cell enriched-CN4 with the tumor boundary CN1 highlighted (right, red arrow).

(K) Barplot depicting top 10 spatial contexts distinct to nodal-positive (n=125) and nodal-negative primary tumor samples (n=62) with corresponding significance levels. Statistical comparisons were conducted using differential enrichment analysis in DESeq2. Selected CN-CN spatial contexts highlighted in red.

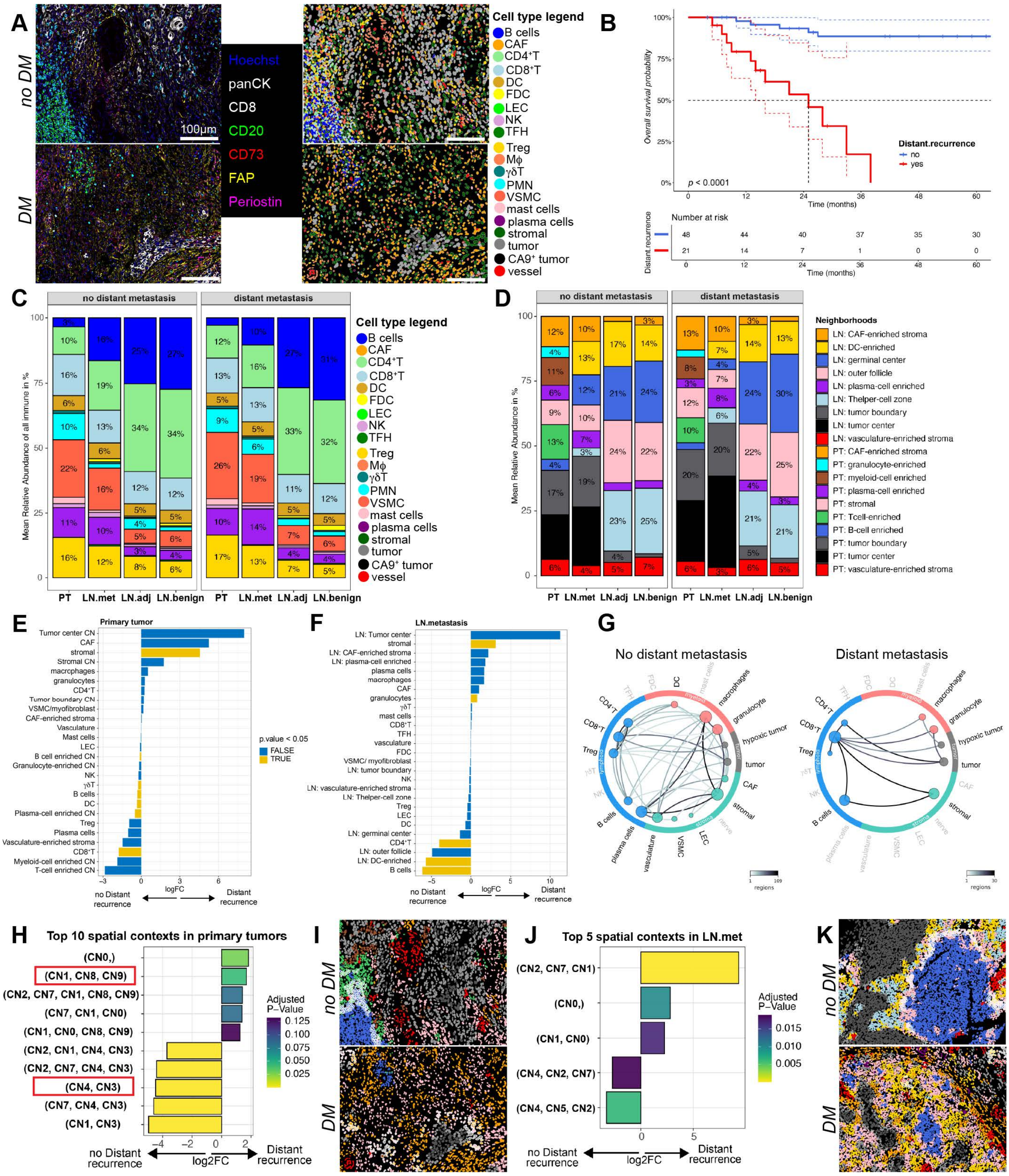

A
no DM
DM
100µm
Hoechst
panCK
CD8
CD20
CD73
FAP
Periostin
Cell type legend
B cells
CAF
CD4+T
CD8+T
DC
FDC
LEC
NK
TFH
Treg
Mϕ
γδT
PMN
VSMC
mast cells
plasma cells
stromal
tumor
CA9+ tumor
vessel
B
Overall survival probability
p < 0.0001
Distant.recurrence
no
yes
Time (months)
Number at risk
Distant.recurrence
48 44 40 37 35 30
21 14 7 1 0 0
C
no distant metastasis
distant metastasis
Mean Relative Abundance of all immune in %
PT
LN.met
LN.adj
LN.benign
D
Mean Relative Abundance in %
Neighborhoods
LN: CAF-enriched stroma
LN: DC-enriched
LN: germinal center
LN: outer follicle
LN: plasma-cell enriched
LN: Thelper-cell zone
LN: tumor boundary
LN: tumor center
LN: vasculature-enriched stroma
PT: CAF-enriched stroma
PT: granulocyte-enriched
PT: myeloid-cell enriched
PT: plasma-cell enriched
PT: stromal
PT: Tcell-enriched
PT: B-cell enriched
PT: tumor boundary
PT: tumor center
PT: vasculature-enriched stroma
E
Primary tumor
Tumor center CN
CAF
stromal
Stromal CN
macrcphages
granulocytes
CD4+T
Tumor boundary CN
VSMC/myofibroblast
CAF-enriched stroma
Vasculature
Mast cells
LEC
B cell enriched CN
Granulocyte-enriched CN
NK
γδT
B cells
DC
Plasma-cell enriched CN
Treg
Plasma cells
Vasculature-enriched stroma
CD8+T
Myeloid-cell enriched CN
T-cell enriched CN
p.value < 0.05
FALSE
TRUE
logFC
no Distant recurrence
Distant recurrence
F
LN.metastasis
LN: Tumor center
stromal
LN: CAF-enriched stroma
LN: plasma-cell enriched
plasma cells
macrophages
CAF
granulocytes
γδT
mast cells
CD8+T
TFH
vasculature
FDC
VSMC/ myofibroblast
LN: tumor boundary
NK
LN: vasculature-enriched stroma
LN: Thelper-cell zone
Treg
LEC
DC
LN: germinal center
CD4+T
LN: outer follicle
LN: DC-enriched
B cells
logFC
no Distant recurrence
Distant recurrence
G
No distant metastasis
Distant metastasis
regions
H
Top 10 spatial contexts in primary tumors
(CN0,)
(CN1, CN8, CN9)
(CN2, CN7, CN1, CN8, CN9)
(CN7, CN1, CN0)
(CN1, CN0, CN8, CN9)
(CN2, CN1, CN4, CN3)
(CN2, CN7, CN4, CN3)
(CN4, CN3)
(CN7, CN4, CN3)
(CN1, CN3)
Adjusted P–Value
log2FC
no Distant recurrence
Distant recurrence
I
no DM
DM
J
Top 5 spatial contexts in LN.met
(CN2, CN7, CN1)
(CN0,)
(CN1, CN0)
(CN4, CN2, CN7)
(CN4, CN5, CN2)
Adjusted P–Value
log2FC
no Distant recurrence
Distant recurrence
K
no DM
DM

Figure S14: Cellular and organizational features within the primary TME and LN metastasis environment linked to distant recurrence. Related to Figure 7.

(A) Representative examples of raw multiplex immunofluorescence images (left) and corresponding cell-type geography maps (right) for features within the primary TME characterizing patients with (bottom) or without distant recurrence (top). Scale bar of 100µm applies to all panels.

(B) Kaplan-Meier survival plot depicting the overall survival probability stratified between patients with or without distant recurrence and corresponding number at risk. Significance was determined using log-rank test.

(C) Stacked barplots summarizing the mean abundance of major cell types within each investigated tissue in patients without (PT, n=118; LN.met, n=52; LN.adjacent, n=26; LN.benign, n=37) or with distant metastasis (PT, n=55; LN.met, n=37; LN.adjacent, n=15; LN.benign, n=20).

(D) Stacked barplots summarizing the mean abundance of all identified cellular neighborhoods within each investigated tissue in patients without (left) or with distant metastasis (right).

(E) Waterfall plot showing the log2-fold change enrichment in major cell types and cellular neighborhoods in PT-regions of cases with (n=55) or without distant recurrence (n=118). Statistical significance was determined using two-sided Wilcoxon-rank sum test corrected for multiple hypothesis testing (BH) with significant results printed in yellow.

(F) Waterfall plot showing the log2-fold change enrichment in major cell types and cellular neighborhoods in the LN metastasis microenvironment between cases with (n=37) or without distant recurrence (n=52). Statistical significance was determined using two-sided Wilcoxon-rank sum test corrected for multiple hypothesis testing (BH) with significant results printed in yellow.

(G) Circos plot depicting characteristic cellular niche networks in PT-regions of cases without (left) or with distant recurrence. Edge color indicates the abundance of these interactions measured as number of regions. Size of the nodes indicates the abundance of the corresponding cell-type to be involved in these cellular niche networks.

(H) Waterfall plot summarizing log2-fold enrichment of neighborhood-neighborhood interactions (spatial contexts) in the primary TME of patients with or without distant recurrence. Color codes indicate adjusted p-values. Statistical significance was determined using DESeq2.

(I) Neighborhood geography map of two representative examples for spatial contexts found in patients without or with distant recurrence. Color codes from D apply. Cells with white outline show the distinct spatial context characteristic for patients without distant recurrence (CN3, CN4) or with distant recurrence (CN1, CN8, CN9). CN legend shown in (D).

(J) Waterfall plot summarizing log2-fold enrichment of neighborhood-neighborhood interactions (spatial contexts) in the metastatic LN microenvironment of patients with or without distant recurrence. Color codes indicate adjusted p-values. Statistical significance was determined using DESeq2.

(K) Neighborhood geography map of two representative examples for spatial contexts found in patients without or with distant recurrence. Color codes from D apply. Cells with white outline show the distinct spatial context characteristic for patients without distant recurrence (CN2, CN4, CN5) or with distant recurrence (CN1, CN2, CN7). CN legend shown in (D).

Abbreviations: DM = distant metastasis; LN.met = LN metastasis; LN.adj = adjacent non-tumor infested region of LN.met; PT = primary tumor

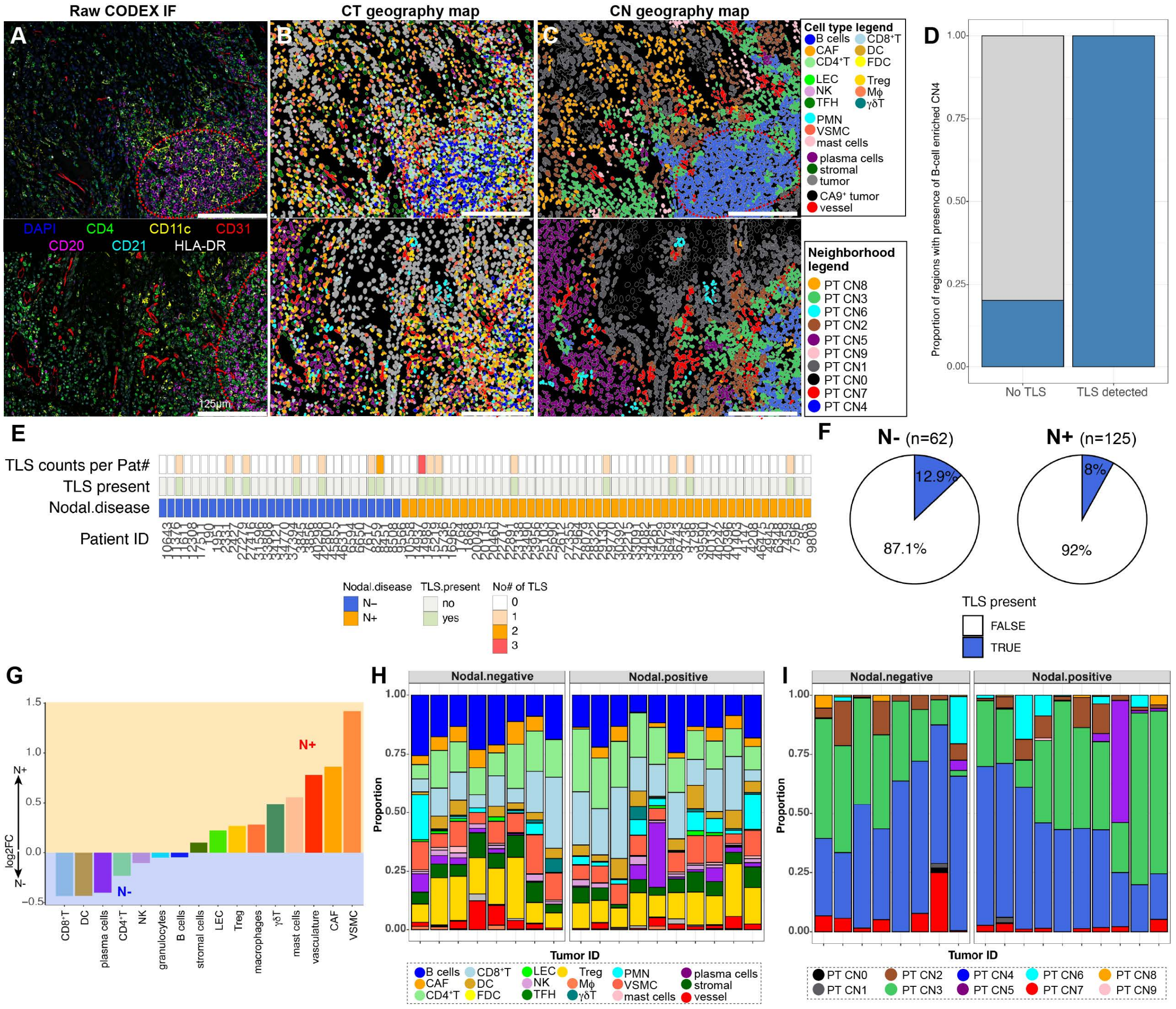

Raw CODEX IF
CT geography map
CN geography map
A
B
C
D
DAPI CD4 CD11c CD31
CD20 CD21 HLA-DR
125μm
Cell type legend
B cells CD8⁺T
CAF DC
CD4⁺T FDC
LEC Treg
NK Mϕ
TFH γδT
PMN
VSMC
mast cells
plasma cells
stromal
tumor
CA9⁺ tumor
vessel
Neighborhood legend
PT CN8
PT CN3
PT CN6
PT CN2
PT CN5
PT CN9
PT CN1
PT CN0
PT CN7
PT CN4
Proportion of regions with presence of B-cell enriched CN4
No TLS
TLS detected
E
TLS counts per Pat#
TLS present
Nodal.disease
Patient ID
Nodal.disease
N−
N+
TLS.present
no
yes
No# of TLS
0
1
2
3
F
N- (n=62)
N+ (n=125)
12.9%
87.1%
8%
92%
TLS present
FALSE
TRUE
G
log2FC
N+
N-
CD8⁺T
DC
plasma cells
CD4⁺T
NK
granulocytes
B cells
stromal cells
LEC
Treg
macrophages
γδT
mast cells
vasculature
CAF
VSMC
H
Nodal.negative
Nodal.positive
Proportion
Tumor ID
B cells CD8⁺T LEC Treg PMN plasma cells
CAF DC NK Mϕ VSMC stromal
CD4⁺T FDC TFH γδT mast cells vessel
I
Nodal.negative
Nodal.positive
Proportion
Tumor ID
PT CN0 PT CN2 PT CN4 PT CN6 PT CN8
PT CN1 PT CN3 PT CN5 PT CN7 PT CN9

Figure S15: Comparison of the cellular and architectural differences observed between the B cell enriched CN4 and manually annotated tertiary lymphoid structures. Related to Figure 7.

(A-C) Representative examples of two tertiary lymphoid structures (TLS; red dotted line) depicted as a raw CODEX image (A), corresponding cell-type geography map (B) and the neighborhood geography map (C). Legend to the right applies. Scale bar of 125µm applies to all panels.

(D) Stacked barplot summarizing the presence of the B cell enriched neighborhood in regions containing manually annotated TLS.

(E) Matrix plot summarizing the presence and absolute counts of manually annotated tertiary-lymphoid structures per patient in the investigated HNSCC discovery cohort grouped by nodal disease status.

(F) Pie chart quantifying the absolute and relative abundances of manually identified TLS within the entire patient cohort stratified by nodal status.

(G) Waterfall plot exhibiting log2-fold changes in cell type composition between manually annotated TLS in nodal-negative (bottom) vs nodal-positive regions (top).

(H) Stacked barplots summarizing the relative cell type abundances in each TLS structure stratified by nodal disease status. Cell-type color legend from (A-C) applies.

(I) Stacked barplots summarizing the relative neighborhood abundances in each TLS structure stratified by nodal disease status. Neighborhood color legend from (A-C) applies.

Table S1: Baseline patient characteristics of the HNSCC validation cohort. Related to Figure 5.

**Clinicopathological features**	**Number of patients (%)**
Total number of patients	6
**Baseline patient demographics**	
Median age at diagnosis (range)	62.0 yrs (60-63)
Sex<br>- Male	 6
Primary tumor location<br>- Oral tongue<br>- Retromolar trigonum<br>- Buccal mucosa	 3<br>2<br>1
p16-status[a]<br>- Negative	 5
T-stage<br>- T3<br>- T4a	 5<br>1
N-stage<br>- N0<br>- N1<br>- N2a<br>- N2b<br>- N2c<br>- N3	 2<br>0<br>0<br>2<br>1<br>1
AJCC 7 staging system<br>- III<br>- IVA<br>- IVB<br>- IVC	 2<br>3<br>1<br>0
Perineural invasion	1/6
Lymphovascular invasion	2/6
Former smoker	4/6
Current smoker	3/6
**Treatments**	
Surgical removal	6/6
Neck dissection	6/6
Adjuvant RTx	5/6
Concurrent adjuvant CTx and RTx	4/6
**Survival outcomes**	
Recurrence<br>- Local<br>- Locoregional<br>- Distant	3/6 (50%)<br>2/6 (33%)<br>0/6<br>2/6 (33%)
Median relapse-free survival (95% CI)	15.0 months (12-NR)
Median distant metastasis free survival (95% CI)	NR
Median follow-up upon initial diagnosis (95% CI)	83 months (48 months - NR)
Median overall survival (95% CI)	34.0 months (12.0-NR)
Deceased	3/6 (50%)

Table S1. Description of the HNSCC validation cohort including baseline patient demographics, tumor characteristics, treatments and survival outcomes.

[a] Data on p16-status was available in 5/6 patients. *Abbreviations: HNSCC = head-and-neck squamous cell carcinoma; OS = overall survival; RFS = relapse-free survival; DMFS = distant-metastasis free survival; CTx = chemotherapy; RTx = radiotherapy; CI = confidence interval*
